# Supplementary material for: Genome-wide identification, characterization and gene expression of BES1 transcription factor family in grapevine (Vitis vinifera L.)
Source: Sci Rep. 2023 Jan 5;13:240. doi: 10.1038/s41598-022-24407-y (PMC9816167; doi:10.1038/s41598-022-24407-y)
Supplement: Supplementary file 3 — Supplementary Information. [file 41598_2022_24407_MOESM3_ESM.zip › Vvi_Atr/Vitis_vinifera.PN40024.v4.dna_sm.toplevel.fa.vs.Amborella_trichopoda.AMTR1.0.dna_sm.toplevel.fa.html/Vvi-5.html]

|  |  |  |  |  |  |  |  |  |  |  |  |  |  |
| --- | --- | --- | --- | --- | --- | --- | --- | --- | --- | --- | --- | --- | --- |
| Duplication depth | Reference chromosome | Collinear blocks | | | | | | | | | | | |
| 1 | Vvi-Vitvi05g00001\_t002 |  | Atr-ERN02251 |  |  |  |  |  |  |  |  |  |  |
| 1 | Vvi-Vitvi05g04000\_t001 |  | \| \| |  |  |  |  |  |  |  |  |  |  |
| 1 | Vvi-Vitvi05g01734\_t001 |  | Atr-ERN02252 |  |  |  |  |  |  |  |  |  |  |
| 1 | Vvi-Vitvi05g00005\_t001 |  | Atr-ERN02253 |  |  |  |  |  |  |  |  |  |  |
| 1 | Vvi-Vitvi05g00006\_t002 |  | Atr-ERN02258 |  |  |  |  |  |  |  |  |  |  |
| 1 | Vvi-Vitvi05g00007\_t001 |  | Atr-ERN02265 |  |  |  |  |  |  |  |  |  |  |
| 1 | Vvi-Vitvi05g00008\_t001 |  | Atr-ERN02266 |  |  |  |  |  |  |  |  |  |  |
| 0 | Vvi-Vitvi05g00009\_t001 |  |  |  |  |  |  |  |  |  |  |  |  |
| 0 | Vvi-Vitvi05g00010\_t001 |  |  |  |  |  |  |  |  |  |  |  |  |
| 0 | Vvi-Vitvi05g00011\_t001 |  |  |  |  |  |  |  |  |  |  |  |  |
| 0 | Vvi-Vitvi05g00012\_t001 |  |  |  |  |  |  |  |  |  |  |  |  |
| 0 | Vvi-Vitvi05g04001\_t001 |  |  |  |  |  |  |  |  |  |  |  |  |
| 0 | Vvi-Vitvi05g04002\_t001 |  |  |  |  |  |  |  |  |  |  |  |  |
| 0 | Vvi-Vitvi05g04003\_t001 |  |  |  |  |  |  |  |  |  |  |  |  |
| 0 | Vvi-Vitvi05g00015\_t001 |  |  |  |  |  |  |  |  |  |  |  |  |
| 0 | Vvi-Vitvi05g04004\_t001 |  |  |  |  |  |  |  |  |  |  |  |  |
| 0 | Vvi-Vitvi05g00016\_t001 |  |  |  |  |  |  |  |  |  |  |  |  |
| 0 | Vvi-Vitvi05g04005\_t001 |  |  |  |  |  |  |  |  |  |  |  |  |
| 0 | Vvi-Vitvi05g01740\_t001 |  |  |  |  |  |  |  |  |  |  |  |  |
| 0 | Vvi-Vitvi05g01741\_t001 |  |  |  |  |  |  |  |  |  |  |  |  |
| 0 | Vvi-Vitvi05g04006\_t001 |  |  |  |  |  |  |  |  |  |  |  |  |
| 0 | Vvi-Vitvi05g00018\_t001 |  |  |  |  |  |  |  |  |  |  |  |  |
| 0 | Vvi-Vitvi05g00020\_t002 |  |  |  |  |  |  |  |  |  |  |  |  |
| 0 | Vvi-Vitvi05g04007\_t001 |  |  |  |  |  |  |  |  |  |  |  |  |
| 0 | Vvi-Vitvi05g04008\_t001 |  |  |  |  |  |  |  |  |  |  |  |  |
| 0 | Vvi-Vitvi05g00026\_t001 |  |  |  |  |  |  |  |  |  |  |  |  |
| 0 | Vvi-Vitvi05g00027\_t001 |  |  |  |  |  |  |  |  |  |  |  |  |
| 0 | Vvi-Vitvi05g00028\_t001 |  |  |  |  |  |  |  |  |  |  |  |  |
| 0 | Vvi-Vitvi05g04009\_t001 |  |  |  |  |  |  |  |  |  |  |  |  |
| 0 | Vvi-Vitvi05g00031\_t001 |  |  |  |  |  |  |  |  |  |  |  |  |
| 0 | Vvi-Vitvi05g00032\_t001 |  |  |  |  |  |  |  |  |  |  |  |  |
| 0 | Vvi-Vitvi05g00033\_t001 |  |  |  |  |  |  |  |  |  |  |  |  |
| 0 | Vvi-Vitvi05g00034\_t001 |  |  |  |  |  |  |  |  |  |  |  |  |
| 0 | Vvi-Vitvi05g00035\_t001 |  |  |  |  |  |  |  |  |  |  |  |  |
| 0 | Vvi-Vitvi05g01742\_t002 |  |  |  |  |  |  |  |  |  |  |  |  |
| 0 | Vvi-Vitvi05g00038\_t001 |  |  |  |  |  |  |  |  |  |  |  |  |
| 0 | Vvi-Vitvi05g00039\_t001 |  |  |  |  |  |  |  |  |  |  |  |  |
| 0 | Vvi-Vitvi05g00040\_t001 |  |  |  |  |  |  |  |  |  |  |  |  |
| 0 | Vvi-Vitvi05g04010\_t001 |  |  |  |  |  |  |  |  |  |  |  |  |
| 0 | Vvi-Vitvi05g01743\_t001 |  |  |  |  |  |  |  |  |  |  |  |  |
| 0 | Vvi-Vitvi05g00041\_t001 |  |  |  |  |  |  |  |  |  |  |  |  |
| 0 | Vvi-Vitvi05g00042\_t001 |  |  |  |  |  |  |  |  |  |  |  |  |
| 0 | Vvi-Vitvi05g00043\_t001 |  |  |  |  |  |  |  |  |  |  |  |  |
| 1 | Vvi-Vitvi05g00044\_t001 |  | Atr-ERN03166 |  |  |  |  |  |  |  |  |  |  |
| 1 | Vvi-Vitvi05g00045\_t001 |  | \| \| |  |  |  |  |  |  |  |  |  |  |
| 1 | Vvi-Vitvi05g04011\_t001 |  | \| \| |  |  |  |  |  |  |  |  |  |  |
| 1 | Vvi-Vitvi05g04012\_t001 |  | \| \| |  |  |  |  |  |  |  |  |  |  |
| 1 | Vvi-Vitvi05g04013\_t001 |  | \| \| |  |  |  |  |  |  |  |  |  |  |
| 1 | Vvi-Vitvi05g00047\_t001 |  | \| \| |  |  |  |  |  |  |  |  |  |  |
| 1 | Vvi-Vitvi05g00048\_t001 |  | \| \| |  |  |  |  |  |  |  |  |  |  |
| 1 | Vvi-Vitvi05g00049\_t001 |  | \| \| |  |  |  |  |  |  |  |  |  |  |
| 1 | Vvi-Vitvi05g00050\_t001 |  | \| \| |  |  |  |  |  |  |  |  |  |  |
| 1 | Vvi-Vitvi05g01724\_t001 |  | Atr-ERN03179 |  |  |  |  |  |  |  |  |  |  |
| 1 | Vvi-Vitvi05g01745\_t001 |  | \| \| |  |  |  |  |  |  |  |  |  |  |
| 1 | Vvi-Vitvi05g00053\_t001 |  | \| \| |  |  |  |  |  |  |  |  |  |  |
| 1 | Vvi-Vitvi05g00054\_t001 |  | \| \| |  |  |  |  |  |  |  |  |  |  |
| 1 | Vvi-Vitvi05g00055\_t001 |  | \| \| |  |  |  |  |  |  |  |  |  |  |
| 1 | Vvi-Vitvi05g00056\_t001 |  | \| \| |  |  |  |  |  |  |  |  |  |  |
| 1 | Vvi-Vitvi05g00057\_t001.1.6037826e |  | \| \| |  |  |  |  |  |  |  |  |  |  |
| 1 | Vvi-Vitvi05g01746\_t001 |  | \| \| |  |  |  |  |  |  |  |  |  |  |
| 1 | Vvi-Vitvi05g01747\_t001 |  | \| \| |  |  |  |  |  |  |  |  |  |  |
| 1 | Vvi-Vitvi05g00060\_t001 |  | Atr-ERN03186 |  |  |  |  |  |  |  |  |  |  |
| 1 | Vvi-Vitvi05g00061\_t001 |  | Atr-ERN03187 |  |  |  |  |  |  |  |  |  |  |
| 1 | Vvi-Vitvi05g04014\_t001 |  | \| \| |  |  |  |  |  |  |  |  |  |  |
| 1 | Vvi-Vitvi05g00062\_t003 |  | \| \| |  |  |  |  |  |  |  |  |  |  |
| 1 | Vvi-Vitvi05g00063\_t006 |  | Atr-ERN03194 |  |  |  |  |  |  |  |  |  |  |
| 1 | Vvi-Vitvi05g00064\_t001 |  | Atr-ERN03195 |  |  |  |  |  |  |  |  |  |  |
| 1 | Vvi-Vitvi05g00065\_t001 |  | Atr-ERN03196 |  |  |  |  |  |  |  |  |  |  |
| 0 | Vvi-Vitvi05g00066\_t001 |  |  |  |  |  |  |  |  |  |  |  |  |
| 0 | Vvi-Vitvi05g00067\_t001 |  |  |  |  |  |  |  |  |  |  |  |  |
| 0 | Vvi-Vitvi05g00068\_t001 |  |  |  |  |  |  |  |  |  |  |  |  |
| 0 | Vvi-Vitvi05g01752\_t001 |  |  |  |  |  |  |  |  |  |  |  |  |
| 0 | Vvi-Vitvi05g00069\_t001 |  |  |  |  |  |  |  |  |  |  |  |  |
| 0 | Vvi-Vitvi05g01753\_t001 |  |  |  |  |  |  |  |  |  |  |  |  |
| 0 | Vvi-Vitvi05g01754\_t001 |  |  |  |  |  |  |  |  |  |  |  |  |
| 0 | Vvi-Vitvi05g04015\_t001 |  |  |  |  |  |  |  |  |  |  |  |  |
| 0 | Vvi-Vitvi05g00071\_t001 |  |  |  |  |  |  |  |  |  |  |  |  |
| 0 | Vvi-Vitvi05g01757\_t001 |  |  |  |  |  |  |  |  |  |  |  |  |
| 0 | Vvi-Vitvi05g01759\_t003 |  |  |  |  |  |  |  |  |  |  |  |  |
| 0 | Vvi-Vitvi05g00072\_t001 |  |  |  |  |  |  |  |  |  |  |  |  |
| 0 | Vvi-Vitvi05g01760\_t001 |  |  |  |  |  |  |  |  |  |  |  |  |
| 0 | Vvi-Vitvi05g01761\_t001 |  |  |  |  |  |  |  |  |  |  |  |  |
| 0 | Vvi-Vitvi05g00073\_t002 |  |  |  |  |  |  |  |  |  |  |  |  |
| 1 | Vvi-Vitvi05g01762\_t001 |  | Atr-ERN16823 |  |  |  |  |  |  |  |  |  |  |
| 1 | Vvi-Vitvi05g04016\_t001 |  | Atr-ERN16821 |  |  |  |  |  |  |  |  |  |  |
| 1 | Vvi-Vitvi05g00075\_t001 |  | Atr-ERN16820 |  |  |  |  |  |  |  |  |  |  |
| 1 | Vvi-Vitvi05g00076\_t001 |  | \| \| |  |  |  |  |  |  |  |  |  |  |
| 1 | Vvi-Vitvi05g01763\_t001 |  | \| \| |  |  |  |  |  |  |  |  |  |  |
| 1 | Vvi-Vitvi05g00077\_t001 |  | \| \| |  |  |  |  |  |  |  |  |  |  |
| 1 | Vvi-Vitvi05g04017\_t001 |  | \| \| |  |  |  |  |  |  |  |  |  |  |
| 1 | Vvi-Vitvi05g01765\_t001 |  | \| \| |  |  |  |  |  |  |  |  |  |  |
| 1 | Vvi-Vitvi05g00079\_t001 |  | Atr-ERN16805 |  |  |  |  |  |  |  |  |  |  |
| 1 | Vvi-Vitvi05g00080\_t001 |  | \| \| |  |  |  |  |  |  |  |  |  |  |
| 1 | Vvi-Vitvi05g00081\_t002 |  | \| \| |  |  |  |  |  |  |  |  |  |  |
| 1 | Vvi-Vitvi05g00082\_t001 |  | Atr-ERN16794 |  |  |  |  |  |  |  |  |  |  |
| 1 | Vvi-Vitvi05g01766\_t001 |  | Atr-ERN16791 |  |  |  |  |  |  |  |  |  |  |
| 1 | Vvi-Vitvi05g04018\_t001 |  | \| \| |  |  |  |  |  |  |  |  |  |  |
| 1 | Vvi-Vitvi05g00083\_t001 |  | Atr-ERN16783 |  |  |  |  |  |  |  |  |  |  |
| 1 | Vvi-Vitvi05g00084\_t001 |  | \| \| |  |  |  |  |  |  |  |  |  |  |
| 1 | Vvi-Vitvi05g00085\_t001 |  | Atr-ERN16776 |  |  |  |  |  |  |  |  |  |  |
| 1 | Vvi-Vitvi05g00086\_t001 |  | \| \| |  |  |  |  |  |  |  |  |  |  |
| 1 | Vvi-Vitvi05g01767\_t001 |  | Atr-ERN16774 |  |  |  |  |  |  |  |  |  |  |
| 1 | Vvi-Vitvi05g00087\_t001 |  | \| \| |  |  |  |  |  |  |  |  |  |  |
| 1 | Vvi-Vitvi05g01768\_t001 |  | Atr-ERN16765 |  |  |  |  |  |  |  |  |  |  |
| 1 | Vvi-Vitvi05g01769\_t001 |  | \| \| |  |  |  |  |  |  |  |  |  |  |
| 1 | Vvi-Vitvi05g04019\_t001 |  | Atr-ERN16762 |  |  |  |  |  |  |  |  |  |  |
| 1 | Vvi-Vitvi05g00089\_t001 |  | Atr-ERN16761 |  |  |  |  |  |  |  |  |  |  |
| 1 | Vvi-Vitvi05g00092\_t001 |  | Atr-ERN16760 |  |  |  |  |  |  |  |  |  |  |
| 1 | Vvi-Vitvi05g00094\_t001 |  | \| \| |  |  |  |  |  |  |  |  |  |  |
| 1 | Vvi-Vitvi05g00096\_t001 |  | Atr-ERN16758 |  |  |  |  |  |  |  |  |  |  |
| 1 | Vvi-Vitvi05g00097\_t001 |  | \| \| |  |  |  |  |  |  |  |  |  |  |
| 1 | Vvi-Vitvi05g00098\_t001 |  | \| \| |  |  |  |  |  |  |  |  |  |  |
| 1 | Vvi-Vitvi05g00100\_t001 |  | \| \| |  |  |  |  |  |  |  |  |  |  |
| 2 | Vvi-Vitvi05g00101\_t002 |  | \| \| |  | Atr-ERN03083 |  |  |  |  |  |  |  |  |
| 2 | Vvi-Vitvi05g00102\_t001 |  | \| \| |  | \| \| |  |  |  |  |  |  |  |  |
| 2 | Vvi-Vitvi05g00104\_t001 |  | \| \| |  | \| \| |  |  |  |  |  |  |  |  |
| 2 | Vvi-Vitvi05g00105\_t001 |  | \| \| |  | \| \| |  |  |  |  |  |  |  |  |
| 2 | Vvi-Vitvi05g00106\_t001 |  | Atr-ERN16745 |  | \| \| |  |  |  |  |  |  |  |  |
| 1 | Vvi-Vitvi05g00107\_t001 |  |  |  | \| \| |  |  |  |  |  |  |  |  |
| 1 | Vvi-Vitvi05g04020\_t001 |  |  |  | \| \| |  |  |  |  |  |  |  |  |
| 2 | Vvi-Vitvi05g00108\_t001 |  | Atr-ERN02129 |  | \| \| |  |  |  |  |  |  |  |  |
| 2 | Vvi-Vitvi05g00109\_t001 |  | \| \| |  | \| \| |  |  |  |  |  |  |  |  |
| 2 | Vvi-Vitvi05g00110\_t001 |  | Atr-ERN02114 |  | \| \| |  |  |  |  |  |  |  |  |
| 2 | Vvi-Vitvi05g04021\_t001 |  | \| \| |  | \| \| |  |  |  |  |  |  |  |  |
| 2 | Vvi-Vitvi05g04022\_t001 |  | \| \| |  | \| \| |  |  |  |  |  |  |  |  |
| 2 | Vvi-Vitvi05g04023\_t001 |  | \| \| |  | \| \| |  |  |  |  |  |  |  |  |
| 2 | Vvi-Vitvi05g00112\_t001 |  | Atr-ERN02113 |  | \| \| |  |  |  |  |  |  |  |  |
| 2 | Vvi-Vitvi05g00113\_t001 |  | Atr-ERN02108 |  | \| \| |  |  |  |  |  |  |  |  |
| 2 | Vvi-Vitvi05g00114\_t001 |  | Atr-ERN02107 |  | \| \| |  |  |  |  |  |  |  |  |
| 2 | Vvi-Vitvi05g01772\_t001 |  | Atr-ERN02102 |  | \| \| |  |  |  |  |  |  |  |  |
| 2 | Vvi-Vitvi05g00115\_t001 |  | Atr-ERN02101 |  | Atr-ERN03090 |  |  |  |  |  |  |  |  |
| 2 | Vvi-Vitvi05g00116\_t001 |  | \| \| |  | \| \| |  |  |  |  |  |  |  |  |
| 2 | Vvi-Vitvi05g00117\_t001 |  | \| \| |  | \| \| |  |  |  |  |  |  |  |  |
| 2 | Vvi-Vitvi05g00118\_t001 |  | \| \| |  | \| \| |  |  |  |  |  |  |  |  |
| 2 | Vvi-Vitvi05g00120\_t001 |  | \| \| |  | \| \| |  |  |  |  |  |  |  |  |
| 2 | Vvi-Vitvi05g00122\_t001 |  | \| \| |  | \| \| |  |  |  |  |  |  |  |  |
| 2 | Vvi-Vitvi05g00123\_t001 |  | \| \| |  | \| \| |  |  |  |  |  |  |  |  |
| 2 | Vvi-Vitvi05g00124\_t001 |  | \| \| |  | Atr-ERN03107 |  |  |  |  |  |  |  |  |
| 2 | Vvi-Vitvi05g00125\_t001 |  | \| \| |  | Atr-ERN03108 |  |  |  |  |  |  |  |  |
| 2 | Vvi-Vitvi05g01773\_t001 |  | \| \| |  | \| \| |  |  |  |  |  |  |  |  |
| 2 | Vvi-Vitvi05g00126\_t001 |  | \| \| |  | Atr-ERN03113 |  |  |  |  |  |  |  |  |
| 2 | Vvi-Vitvi05g00127\_t001 |  | \| \| |  | \| \| |  |  |  |  |  |  |  |  |
| 2 | Vvi-Vitvi05g00128\_t001 |  | \| \| |  | \| \| |  |  |  |  |  |  |  |  |
| 2 | Vvi-Vitvi05g00129\_t002 |  | \| \| |  | Atr-ERN03122 |  |  |  |  |  |  |  |  |
| 2 | Vvi-Vitvi05g00130\_t001 |  | Atr-ERN02086 |  | Atr-ERN03123 |  |  |  |  |  |  |  |  |
| 1 | Vvi-Vitvi05g00132\_t001 |  |  |  | Atr-ERN03125 |  |  |  |  |  |  |  |  |
| 1 | Vvi-Vitvi05g01774\_t001 |  |  |  | Atr-ERN03126 |  |  |  |  |  |  |  |  |
| 1 | Vvi-Vitvi05g01775\_t001 |  |  |  | \| \| |  |  |  |  |  |  |  |  |
| 1 | Vvi-Vitvi05g00134\_t001 |  |  |  | \| \| |  |  |  |  |  |  |  |  |
| 1 | Vvi-Vitvi05g00135\_t001 |  |  |  | \| \| |  |  |  |  |  |  |  |  |
| 1 | Vvi-Vitvi05g04024\_t001 |  |  |  | \| \| |  |  |  |  |  |  |  |  |
| 1 | Vvi-Vitvi05g00136\_t001 |  |  |  | \| \| |  |  |  |  |  |  |  |  |
| 1 | Vvi-Vitvi05g00137\_t001 |  |  |  | Atr-ERN03135 |  |  |  |  |  |  |  |  |
| 1 | Vvi-Vitvi05g00138\_t001 |  |  |  | \| \| |  |  |  |  |  |  |  |  |
| 1 | Vvi-Vitvi05g00139\_t001 |  |  |  | \| \| |  |  |  |  |  |  |  |  |
| 1 | Vvi-Vitvi05g00141\_t001 |  |  |  | Atr-ERN03141 |  |  |  |  |  |  |  |  |
| 1 | Vvi-Vitvi05g00142\_t001 |  |  |  | Atr-ERN03143 |  |  |  |  |  |  |  |  |
| 1 | Vvi-Vitvi05g01776\_t001 |  | Atr-ERN03297 |  |  |  |  |  |  |  |  |  |  |
| 1 | Vvi-Vitvi05g01777\_t001 |  | \| \| |  |  |  |  |  |  |  |  |  |  |
| 1 | Vvi-Vitvi05g01778\_t001 |  | \| \| |  |  |  |  |  |  |  |  |  |  |
| 1 | Vvi-Vitvi05g01780\_t001 |  | \| \| |  |  |  |  |  |  |  |  |  |  |
| 1 | Vvi-Vitvi05g04025\_t001 |  | \| \| |  |  |  |  |  |  |  |  |  |  |
| 1 | Vvi-Vitvi05g01782\_t001 |  | \| \| |  |  |  |  |  |  |  |  |  |  |
| 1 | Vvi-Vitvi05g00143\_t001 |  | Atr-ERN03301 |  |  |  |  |  |  |  |  |  |  |
| 1 | Vvi-Vitvi05g01783\_t001 |  | Atr-ERN03302 |  |  |  |  |  |  |  |  |  |  |
| 1 | Vvi-Vitvi05g00144\_t001 |  | Atr-ERN03303 |  |  |  |  |  |  |  |  |  |  |
| 1 | Vvi-Vitvi05g00145\_t001 |  | Atr-ERN03304 |  |  |  |  |  |  |  |  |  |  |
| 1 | Vvi-Vitvi05g00146\_t001 |  | \| \| |  |  |  |  |  |  |  |  |  |  |
| 1 | Vvi-Vitvi05g00147\_t001 |  | \| \| |  |  |  |  |  |  |  |  |  |  |
| 2 | Vvi-Vitvi05g01784\_t001 |  | \| \| |  | Atr-ERN03315 |  |  |  |  |  |  |  |  |
| 2 | Vvi-Vitvi05g00149\_t001 |  | \| \| |  | Atr-ERN03312 |  |  |  |  |  |  |  |  |
| 2 | Vvi-Vitvi05g00150\_t001 |  | Atr-ERN03309 |  | \| \| |  |  |  |  |  |  |  |  |
| 2 | Vvi-Vitvi05g01786\_t001 |  | \| \| |  | \| \| |  |  |  |  |  |  |  |  |
| 2 | Vvi-Vitvi05g01787\_t001 |  | \| \| |  | \| \| |  |  |  |  |  |  |  |  |
| 2 | Vvi-Vitvi05g01788\_t001 |  | \| \| |  | \| \| |  |  |  |  |  |  |  |  |
| 2 | Vvi-Vitvi05g01789\_t001 |  | \| \| |  | \| \| |  |  |  |  |  |  |  |  |
| 2 | Vvi-Vitvi05g04026\_t001 |  | \| \| |  | \| \| |  |  |  |  |  |  |  |  |
| 2 | Vvi-Vitvi05g00151\_t001 |  | \| \| |  | \| \| |  |  |  |  |  |  |  |  |
| 2 | Vvi-Vitvi05g04027\_t001 |  | \| \| |  | \| \| |  |  |  |  |  |  |  |  |
| 2 | Vvi-Vitvi05g01790\_t003 |  | \| \| |  | \| \| |  |  |  |  |  |  |  |  |
| 2 | Vvi-Vitvi05g00153\_t001 |  | Atr-ERN03320 |  | \| \| |  |  |  |  |  |  |  |  |
| 1 | Vvi-Vitvi05g04028\_t001 |  |  |  | \| \| |  |  |  |  |  |  |  |  |
| 1 | Vvi-Vitvi05g00156\_t001 |  |  |  | \| \| |  |  |  |  |  |  |  |  |
| 1 | Vvi-Vitvi05g00157\_t001 |  |  |  | \| \| |  |  |  |  |  |  |  |  |
| 1 | Vvi-Vitvi05g00158\_t002 |  |  |  | \| \| |  |  |  |  |  |  |  |  |
| 1 | Vvi-Vitvi05g04029\_t001 |  |  |  | \| \| |  |  |  |  |  |  |  |  |
| 1 | Vvi-Vitvi05g00159\_t001 |  |  |  | \| \| |  |  |  |  |  |  |  |  |
| 1 | Vvi-Vitvi05g00160\_t001 |  |  |  | \| \| |  |  |  |  |  |  |  |  |
| 1 | Vvi-Vitvi05g00161\_t001 |  |  |  | Atr-ERN03294 |  |  |  |  |  |  |  |  |
| 1 | Vvi-Vitvi05g04030\_t001 |  |  |  | \| \| |  |  |  |  |  |  |  |  |
| 1 | Vvi-Vitvi05g00163\_t001 |  |  |  | Atr-ERN03292 |  |  |  |  |  |  |  |  |
| 1 | Vvi-Vitvi05g00164\_t001 |  |  |  | Atr-ERN03290 |  |  |  |  |  |  |  |  |
| 1 | Vvi-Vitvi05g00166\_t001 |  |  |  | Atr-ERN03288 |  |  |  |  |  |  |  |  |
| 1 | Vvi-Vitvi05g00167\_t001 |  |  |  | Atr-ERN03285 |  |  |  |  |  |  |  |  |
| 1 | Vvi-Vitvi05g00168\_t001 |  |  |  | Atr-ERN03282 |  |  |  |  |  |  |  |  |
| 1 | Vvi-Vitvi05g00169\_t001 |  |  |  | Atr-ERN03281 |  |  |  |  |  |  |  |  |
| 1 | Vvi-Vitvi05g00170\_t001 |  |  |  | Atr-ERN03279 |  |  |  |  |  |  |  |  |
| 1 | Vvi-Vitvi05g04031\_t001 |  |  |  | Atr-ERN03278 |  |  |  |  |  |  |  |  |
| 0 | Vvi-Vitvi05g04032\_t002 |  |  |  |  |  |  |  |  |  |  |  |  |
| 0 | Vvi-Vitvi05g04033\_t001 |  |  |  |  |  |  |  |  |  |  |  |  |
| 0 | Vvi-Vitvi05g04036\_t001 |  |  |  |  |  |  |  |  |  |  |  |  |
| 0 | Vvi-Vitvi05g04037\_t001 |  |  |  |  |  |  |  |  |  |  |  |  |
| 0 | Vvi-Vitvi05g04038\_t001 |  |  |  |  |  |  |  |  |  |  |  |  |
| 0 | Vvi-Vitvi05g01801\_t001 |  |  |  |  |  |  |  |  |  |  |  |  |
| 0 | Vvi-Vitvi05g00177\_t001 |  |  |  |  |  |  |  |  |  |  |  |  |
| 0 | Vvi-Vitvi05g01805\_t001 |  |  |  |  |  |  |  |  |  |  |  |  |
| 0 | Vvi-Vitvi05g04039\_t001 |  |  |  |  |  |  |  |  |  |  |  |  |
| 0 | Vvi-Vitvi05g01808\_t001 |  |  |  |  |  |  |  |  |  |  |  |  |
| 0 | Vvi-Vitvi05g00179\_t001 |  |  |  |  |  |  |  |  |  |  |  |  |
| 0 | Vvi-Vitvi05g04040\_t001 |  |  |  |  |  |  |  |  |  |  |  |  |
| 0 | Vvi-Vitvi05g04041\_t001 |  |  |  |  |  |  |  |  |  |  |  |  |
| 0 | Vvi-Vitvi05g00181\_t001 |  |  |  |  |  |  |  |  |  |  |  |  |
| 0 | Vvi-Vitvi05g04042\_t001 |  |  |  |  |  |  |  |  |  |  |  |  |
| 0 | Vvi-Vitvi05g01811\_t001 |  |  |  |  |  |  |  |  |  |  |  |  |
| 0 | Vvi-Vitvi05g04043\_t001 |  |  |  |  |  |  |  |  |  |  |  |  |
| 0 | Vvi-Vitvi05g04044\_t001 |  |  |  |  |  |  |  |  |  |  |  |  |
| 0 | Vvi-Vitvi05g04045\_t001 |  |  |  |  |  |  |  |  |  |  |  |  |
| 0 | Vvi-Vitvi05g00231\_t001 |  |  |  |  |  |  |  |  |  |  |  |  |
| 0 | Vvi-Vitvi05g00229\_t001 |  |  |  |  |  |  |  |  |  |  |  |  |
| 0 | Vvi-Vitvi05g00186\_t002 |  |  |  |  |  |  |  |  |  |  |  |  |
| 0 | Vvi-Vitvi05g00227\_t001 |  |  |  |  |  |  |  |  |  |  |  |  |
| 0 | Vvi-Vitvi05g00225\_t001 |  |  |  |  |  |  |  |  |  |  |  |  |
| 0 | Vvi-Vitvi05g00223\_t001 |  |  |  |  |  |  |  |  |  |  |  |  |
| 0 | Vvi-Vitvi05g04046\_t001 |  |  |  |  |  |  |  |  |  |  |  |  |
| 1 | Vvi-Vitvi05g04047\_t001 |  | Atr-ERN03275 |  |  |  |  |  |  |  |  |  |  |
| 1 | Vvi-Vitvi05g00218\_t001 |  | Atr-ERN03273 |  |  |  |  |  |  |  |  |  |  |
| 1 | Vvi-Vitvi05g00217\_t004 |  | \| \| |  |  |  |  |  |  |  |  |  |  |
| 1 | Vvi-Vitvi05g01830\_t001 |  | \| \| |  |  |  |  |  |  |  |  |  |  |
| 1 | Vvi-Vitvi05g00215\_t001 |  | Atr-ERN03270 |  |  |  |  |  |  |  |  |  |  |
| 1 | Vvi-Vitvi05g00214\_t001 |  | Atr-ERN03269 |  |  |  |  |  |  |  |  |  |  |
| 1 | Vvi-Vitvi05g00213\_t001 |  | \| \| |  |  |  |  |  |  |  |  |  |  |
| 1 | Vvi-Vitvi05g00212\_t001 |  | Atr-ERN03258 |  |  |  |  |  |  |  |  |  |  |
| 1 | Vvi-Vitvi05g00210\_t001 |  | \| \| |  |  |  |  |  |  |  |  |  |  |
| 1 | Vvi-Vitvi05g04048\_t001 |  | \| \| |  |  |  |  |  |  |  |  |  |  |
| 1 | Vvi-Vitvi05g00209\_t001 |  | Atr-ERN03256 |  |  |  |  |  |  |  |  |  |  |
| 1 | Vvi-Vitvi05g00208\_t001 |  | Atr-ERN03254 |  |  |  |  |  |  |  |  |  |  |
| 1 | Vvi-Vitvi05g00207\_t001 |  | \| \| |  |  |  |  |  |  |  |  |  |  |
| 1 | Vvi-Vitvi05g00206\_t001 |  | \| \| |  |  |  |  |  |  |  |  |  |  |
| 1 | Vvi-Vitvi05g00205\_t001 |  | \| \| |  |  |  |  |  |  |  |  |  |  |
| 1 | Vvi-Vitvi05g04049\_t001 |  | \| \| |  |  |  |  |  |  |  |  |  |  |
| 1 | Vvi-Vitvi05g01828\_t001 |  | \| \| |  |  |  |  |  |  |  |  |  |  |
| 1 | Vvi-Vitvi05g00204\_t001 |  | \| \| |  |  |  |  |  |  |  |  |  |  |
| 1 | Vvi-Vitvi05g04050\_t001 |  | \| \| |  |  |  |  |  |  |  |  |  |  |
| 1 | Vvi-Vitvi05g04051\_t001 |  | \| \| |  |  |  |  |  |  |  |  |  |  |
| 1 | Vvi-Vitvi05g04052\_t001 |  | \| \| |  |  |  |  |  |  |  |  |  |  |
| 1 | Vvi-Vitvi05g04053\_t001 |  | \| \| |  |  |  |  |  |  |  |  |  |  |
| 1 | Vvi-Vitvi05g04054\_t001 |  | \| \| |  |  |  |  |  |  |  |  |  |  |
| 1 | Vvi-Vitvi05g00198\_t001 |  | \| \| |  |  |  |  |  |  |  |  |  |  |
| 1 | Vvi-Vitvi05g04055\_t001 |  | \| \| |  |  |  |  |  |  |  |  |  |  |
| 1 | Vvi-Vitvi05g00197\_t001 |  | \| \| |  |  |  |  |  |  |  |  |  |  |
| 1 | Vvi-Vitvi05g04056\_t001 |  | \| \| |  |  |  |  |  |  |  |  |  |  |
| 1 | Vvi-Vitvi05g04057\_t001 |  | \| \| |  |  |  |  |  |  |  |  |  |  |
| 1 | Vvi-Vitvi05g04058\_t001 |  | \| \| |  |  |  |  |  |  |  |  |  |  |
| 1 | Vvi-Vitvi05g04059\_t001 |  | Atr-ERN03251 |  |  |  |  |  |  |  |  |  |  |
| 1 | Vvi-Vitvi05g00196\_t001 |  | \| \| |  |  |  |  |  |  |  |  |  |  |
| 1 | Vvi-Vitvi05g04060\_t001 |  | \| \| |  |  |  |  |  |  |  |  |  |  |
| 1 | Vvi-Vitvi05g04061\_t001 |  | \| \| |  |  |  |  |  |  |  |  |  |  |
| 1 | Vvi-Vitvi05g00195\_t001 |  | \| \| |  |  |  |  |  |  |  |  |  |  |
| 1 | Vvi-Vitvi05g04062\_t001 |  | \| \| |  |  |  |  |  |  |  |  |  |  |
| 1 | Vvi-Vitvi05g04063\_t001 |  | \| \| |  |  |  |  |  |  |  |  |  |  |
| 1 | Vvi-Vitvi05g04064\_t001 |  | \| \| |  |  |  |  |  |  |  |  |  |  |
| 1 | Vvi-Vitvi05g04065\_t001 |  | \| \| |  |  |  |  |  |  |  |  |  |  |
| 1 | Vvi-Vitvi05g04066\_t001 |  | \| \| |  |  |  |  |  |  |  |  |  |  |
| 1 | Vvi-Vitvi05g00191\_t001 |  | \| \| |  |  |  |  |  |  |  |  |  |  |
| 1 | Vvi-Vitvi05g00190\_t001 |  | \| \| |  |  |  |  |  |  |  |  |  |  |
| 1 | Vvi-Vitvi05g04067\_t001 |  | \| \| |  |  |  |  |  |  |  |  |  |  |
| 1 | Vvi-Vitvi05g04068\_t001 |  | \| \| |  |  |  |  |  |  |  |  |  |  |
| 1 | Vvi-Vitvi05g01819\_t001 |  | \| \| |  |  |  |  |  |  |  |  |  |  |
| 1 | Vvi-Vitvi05g01815\_t001 |  | \| \| |  |  |  |  |  |  |  |  |  |  |
| 1 | Vvi-Vitvi05g04069\_t001 |  | \| \| |  |  |  |  |  |  |  |  |  |  |
| 1 | Vvi-Vitvi05g04070\_t001 |  | Atr-ERN03237 |  |  |  |  |  |  |  |  |  |  |
| 1 | Vvi-Vitvi05g00183\_t001 |  | \| \| |  |  |  |  |  |  |  |  |  |  |
| 1 | Vvi-Vitvi05g04071\_t001 |  | \| \| |  |  |  |  |  |  |  |  |  |  |
| 2 | Vvi-Vitvi05g04072\_t001 |  | \| \| |  | Atr-ERN01965 |  |  |  |  |  |  |  |  |
| 2 | Vvi-Vitvi05g04073\_t001 |  | \| \| |  | \| \| |  |  |  |  |  |  |  |  |
| 3 | Vvi-Vitvi05g00235\_t001 |  | \| \| |  | \| \| |  | Atr-ERN01958 |  |  |  |  |  |  |
| 3 | Vvi-Vitvi05g00236\_t001 |  | \| \| |  | \| \| |  | Atr-ERN01957 |  |  |  |  |  |  |
| 3 | Vvi-Vitvi05g00237\_t001 |  | \| \| |  | \| \| |  | Atr-ERN01956 |  |  |  |  |  |  |
| 3 | Vvi-Vitvi05g00239\_t003 |  | \| \| |  | \| \| |  | Atr-ERN01955 |  |  |  |  |  |  |
| 3 | Vvi-Vitvi05g00241\_t001 |  | \| \| |  | \| \| |  | Atr-ERN01954 |  |  |  |  |  |  |
| 2 | Vvi-Vitvi05g00242\_t001 |  | \| \| |  | Atr-ERN01983 |  |  |  |  |  |  |  |  |
| 2 | Vvi-Vitvi05g00243\_t002 |  | \| \| |  | \| \| |  |  |  |  |  |  |  |  |
| 2 | Vvi-Vitvi05g00244\_t001 |  | \| \| |  | Atr-ERN01986 |  |  |  |  |  |  |  |  |
| 2 | Vvi-Vitvi05g00245\_t001 |  | \| \| |  | Atr-ERN01987 |  |  |  |  |  |  |  |  |
| 2 | Vvi-Vitvi05g00246\_t003 |  | \| \| |  | Atr-ERN01990 |  |  |  |  |  |  |  |  |
| 2 | Vvi-Vitvi05g00247\_t001 |  | \| \| |  | Atr-ERN01991 |  |  |  |  |  |  |  |  |
| 2 | Vvi-Vitvi05g00248\_t001 |  | \| \| |  | \| \| |  |  |  |  |  |  |  |  |
| 2 | Vvi-Vitvi05g00249\_t001 |  | \| \| |  | Atr-ERN01995 |  |  |  |  |  |  |  |  |
| 2 | Vvi-Vitvi05g04074\_t001 |  | \| \| |  | \| \| |  |  |  |  |  |  |  |  |
| 2 | Vvi-Vitvi05g00250\_t001 |  | \| \| |  | Atr-ERN01997 |  |  |  |  |  |  |  |  |
| 2 | Vvi-Vitvi05g04075\_t001 |  | \| \| |  | \| \| |  |  |  |  |  |  |  |  |
| 2 | Vvi-Vitvi05g00252\_t001 |  | \| \| |  | \| \| |  |  |  |  |  |  |  |  |
| 2 | Vvi-Vitvi05g00253\_t001 |  | \| \| |  | \| \| |  |  |  |  |  |  |  |  |
| 2 | Vvi-Vitvi05g01832\_t001 |  | \| \| |  | Atr-ERN02010 |  |  |  |  |  |  |  |  |
| 2 | Vvi-Vitvi05g00254\_t001 |  | Atr-ERN03218 |  | Atr-ERN02011 |  |  |  |  |  |  |  |  |
| 1 | Vvi-Vitvi05g00255\_t001 |  |  |  | Atr-ERN02012 |  |  |  |  |  |  |  |  |
| 1 | Vvi-Vitvi05g00256\_t001 |  |  |  | Atr-ERN02014 |  |  |  |  |  |  |  |  |
| 1 | Vvi-Vitvi05g01833\_t001 |  |  |  | \| \| |  |  |  |  |  |  |  |  |
| 1 | Vvi-Vitvi05g00258\_t001 |  |  |  | Atr-ERN02015 |  |  |  |  |  |  |  |  |
| 1 | Vvi-Vitvi05g00259\_t001 |  |  |  | Atr-ERN02018 |  |  |  |  |  |  |  |  |
| 1 | Vvi-Vitvi05g00260\_t001 |  |  |  | Atr-ERN02022 |  |  |  |  |  |  |  |  |
| 1 | Vvi-Vitvi05g00261\_t001 |  |  |  | Atr-ERN02024 |  |  |  |  |  |  |  |  |
| 1 | Vvi-Vitvi05g00262\_t001 |  |  |  | \| \| |  |  |  |  |  |  |  |  |
| 1 | Vvi-Vitvi05g01834\_t001 |  |  |  | \| \| |  |  |  |  |  |  |  |  |
| 1 | Vvi-Vitvi05g00264\_t001 |  |  |  | Atr-ERN02030 |  |  |  |  |  |  |  |  |
| 1 | Vvi-Vitvi05g01835\_t001 |  |  |  | Atr-ERN02032 |  |  |  |  |  |  |  |  |
| 1 | Vvi-Vitvi05g00265\_t001 |  |  |  | Atr-ERN02039 |  |  |  |  |  |  |  |  |
| 1 | Vvi-Vitvi05g00266\_t001 |  |  |  | Atr-ERN02040 |  |  |  |  |  |  |  |  |
| 1 | Vvi-Vitvi05g01836\_t001 |  |  |  | Atr-ERN02050 |  |  |  |  |  |  |  |  |
| 1 | Vvi-Vitvi05g00267\_t001 |  |  |  | Atr-ERN02051 |  |  |  |  |  |  |  |  |
| 1 | Vvi-Vitvi05g01837\_t001 |  |  |  | \| \| |  |  |  |  |  |  |  |  |
| 1 | Vvi-Vitvi05g04076\_t001 |  |  |  | \| \| |  |  |  |  |  |  |  |  |
| 1 | Vvi-Vitvi05g04077\_t001 |  |  |  | \| \| |  |  |  |  |  |  |  |  |
| 1 | Vvi-Vitvi05g04078\_t001 |  |  |  | \| \| |  |  |  |  |  |  |  |  |
| 1 | Vvi-Vitvi05g04079\_t001 |  |  |  | \| \| |  |  |  |  |  |  |  |  |
| 1 | Vvi-Vitvi05g01842\_t001 |  |  |  | \| \| |  |  |  |  |  |  |  |  |
| 1 | Vvi-Vitvi05g01843\_t001 |  |  |  | Atr-ERN02054 |  |  |  |  |  |  |  |  |
| 1 | Vvi-Vitvi05g01726\_t001 |  |  |  | \| \| |  |  |  |  |  |  |  |  |
| 1 | Vvi-Vitvi05g04080\_t001 |  |  |  | \| \| |  |  |  |  |  |  |  |  |
| 1 | Vvi-Vitvi05g04081\_t001 |  |  |  | \| \| |  |  |  |  |  |  |  |  |
| 1 | Vvi-Vitvi05g01728\_t001 |  |  |  | \| \| |  |  |  |  |  |  |  |  |
| 1 | Vvi-Vitvi05g01729\_t001 |  |  |  | \| \| |  |  |  |  |  |  |  |  |
| 1 | Vvi-Vitvi05g01727\_t001 |  |  |  | \| \| |  |  |  |  |  |  |  |  |
| 1 | Vvi-Vitvi05g00270\_t001 |  |  |  | Atr-ERN02063 |  |  |  |  |  |  |  |  |
| 1 | Vvi-Vitvi05g00271\_t001 |  |  |  | \| \| |  |  |  |  |  |  |  |  |
| 1 | Vvi-Vitvi05g00272\_t001 |  |  |  | \| \| |  |  |  |  |  |  |  |  |
| 1 | Vvi-Vitvi05g00273\_t001 |  |  |  | \| \| |  |  |  |  |  |  |  |  |
| 1 | Vvi-Vitvi05g00274\_t001 |  |  |  | Atr-ERN02074 |  |  |  |  |  |  |  |  |
| 1 | Vvi-Vitvi05g00275\_t001 |  |  |  | Atr-ERN02076 |  |  |  |  |  |  |  |  |
| 1 | Vvi-Vitvi05g00276\_t001 |  |  |  | Atr-ERN02080 |  |  |  |  |  |  |  |  |
| 1 | Vvi-Vitvi05g00277\_t001 |  |  |  | \| \| |  |  |  |  |  |  |  |  |
| 1 | Vvi-Vitvi05g00278\_t001 |  |  |  | Atr-ERN02082 |  |  |  |  |  |  |  |  |
| 0 | Vvi-Vitvi05g00279\_t001 |  |  |  |  |  |  |  |  |  |  |  |  |
| 0 | Vvi-Vitvi05g00280\_t001 |  |  |  |  |  |  |  |  |  |  |  |  |
| 1 | Vvi-Vitvi05g00281\_t001 |  | Atr-ERN02249 |  |  |  |  |  |  |  |  |  |  |
| 1 | Vvi-Vitvi05g00282\_t001 |  | Atr-ERN02241 |  |  |  |  |  |  |  |  |  |  |
| 1 | Vvi-Vitvi05g00284\_t001 |  | Atr-ERN02240 |  |  |  |  |  |  |  |  |  |  |
| 1 | Vvi-Vitvi05g00285\_t001 |  | \| \| |  |  |  |  |  |  |  |  |  |  |
| 1 | Vvi-Vitvi05g00286\_t001 |  | Atr-ERN02239 |  |  |  |  |  |  |  |  |  |  |
| 1 | Vvi-Vitvi05g00287\_t001.1.6037826e |  | Atr-ERN02236 |  |  |  |  |  |  |  |  |  |  |
| 1 | Vvi-Vitvi05g00288\_t001 |  | Atr-ERN02234 |  |  |  |  |  |  |  |  |  |  |
| 1 | Vvi-Vitvi05g00289\_t001 |  | \| \| |  |  |  |  |  |  |  |  |  |  |
| 1 | Vvi-Vitvi05g00290\_t001 |  | \| \| |  |  |  |  |  |  |  |  |  |  |
| 1 | Vvi-Vitvi05g00291\_t001 |  | \| \| |  |  |  |  |  |  |  |  |  |  |
| 1 | Vvi-Vitvi05g00292\_t001 |  | Atr-ERN02216 |  |  |  |  |  |  |  |  |  |  |
| 1 | Vvi-Vitvi05g00293\_t001 |  | Atr-ERN02208 |  |  |  |  |  |  |  |  |  |  |
| 1 | Vvi-Vitvi05g00294\_t001 |  | Atr-ERN02206 |  |  |  |  |  |  |  |  |  |  |
| 2 | Vvi-Vitvi05g00295\_t001 |  | \| \| |  | Atr-ERN16958 |  |  |  |  |  |  |  |  |
| 2 | Vvi-Vitvi05g00296\_t001 |  | \| \| |  | \| \| |  |  |  |  |  |  |  |  |
| 2 | Vvi-Vitvi05g04082\_t001 |  | \| \| |  | \| \| |  |  |  |  |  |  |  |  |
| 2 | Vvi-Vitvi05g00299\_t001 |  | \| \| |  | Atr-ERN16970 |  |  |  |  |  |  |  |  |
| 2 | Vvi-Vitvi05g00300\_t001 |  | \| \| |  | \| \| |  |  |  |  |  |  |  |  |
| 2 | Vvi-Vitvi05g00301\_t001 |  | \| \| |  | Atr-ERN16975 |  |  |  |  |  |  |  |  |
| 3 | Vvi-Vitvi05g00302\_t001 |  | \| \| |  | Atr-ERN16979 |  | Atr-ERN16979 |  |  |  |  |  |  |
| 3 | Vvi-Vitvi05g00303\_t001 |  | \| \| |  | Atr-ERN16985 |  | \| \| |  |  |  |  |  |  |
| 3 | Vvi-Vitvi05g00305\_t001 |  | \| \| |  | Atr-ERN16986 |  | \| \| |  |  |  |  |  |  |
| 3 | Vvi-Vitvi05g04083\_t001 |  | \| \| |  | \| \| |  | \| \| |  |  |  |  |  |  |
| 3 | Vvi-Vitvi05g00309\_t001 |  | \| \| |  | Atr-ERN16990 |  | \| \| |  |  |  |  |  |  |
| 3 | Vvi-Vitvi05g01845\_t001 |  | \| \| |  | Atr-ERN17006 |  | \| \| |  |  |  |  |  |  |
| 3 | Vvi-Vitvi05g00310\_t001 |  | \| \| |  | Atr-ERN17009 |  | \| \| |  |  |  |  |  |  |
| 3 | Vvi-Vitvi05g00311\_t001 |  | \| \| |  | Atr-ERN17011 |  | \| \| |  |  |  |  |  |  |
| 3 | Vvi-Vitvi05g00312\_t001 |  | \| \| |  | Atr-ERN17013 |  | \| \| |  |  |  |  |  |  |
| 3 | Vvi-Vitvi05g00313\_t001 |  | \| \| |  | Atr-ERN02173 |  | \| \| |  |  |  |  |  |  |
| 3 | Vvi-Vitvi05g00314\_t001 |  | \| \| |  | Atr-ERN02174 |  | \| \| |  |  |  |  |  |  |
| 3 | Vvi-Vitvi05g00315\_t001 |  | \| \| |  | Atr-ERN02175 |  | \| \| |  |  |  |  |  |  |
| 3 | Vvi-Vitvi05g04084\_t001 |  | \| \| |  | Atr-ERN02180 |  | \| \| |  |  |  |  |  |  |
| 3 | Vvi-Vitvi05g00319\_t001 |  | Atr-ERN02187 |  | Atr-ERN02187 |  | \| \| |  |  |  |  |  |  |
| 2 | Vvi-Vitvi05g00320\_t001 |  |  |  | \| \| |  | \| \| |  |  |  |  |  |  |
| 2 | Vvi-Vitvi05g00321\_t001 |  |  |  | \| \| |  | \| \| |  |  |  |  |  |  |
| 2 | Vvi-Vitvi05g00322\_t001 |  |  |  | Atr-ERN02190 |  | \| \| |  |  |  |  |  |  |
| 2 | Vvi-Vitvi05g00323\_t001 |  |  |  | Atr-ERN02193 |  | \| \| |  |  |  |  |  |  |
| 1 | Vvi-Vitvi05g00325\_t001 |  |  |  |  |  | Atr-ERN16964 |  |  |  |  |  |  |
| 1 | Vvi-Vitvi05g04085\_t001 |  |  |  |  |  | \| \| |  |  |  |  |  |  |
| 1 | Vvi-Vitvi05g00326\_t001 |  |  |  |  |  | Atr-ERN16955 |  |  |  |  |  |  |
| 1 | Vvi-Vitvi05g04086\_t001 |  |  |  |  |  | \| \| |  |  |  |  |  |  |
| 1 | Vvi-Vitvi05g00328\_t001 |  |  |  |  |  | Atr-ERN16954 |  |  |  |  |  |  |
| 1 | Vvi-Vitvi05g01846\_t001 |  |  |  |  |  | \| \| |  |  |  |  |  |  |
| 1 | Vvi-Vitvi05g00329\_t001 |  |  |  |  |  | Atr-ERN16950 |  |  |  |  |  |  |
| 1 | Vvi-Vitvi05g04087\_t001 |  |  |  |  |  | \| \| |  |  |  |  |  |  |
| 1 | Vvi-Vitvi05g00330\_t001 |  |  |  |  |  | \| \| |  |  |  |  |  |  |
| 1 | Vvi-Vitvi05g01847\_t001 |  |  |  |  |  | \| \| |  |  |  |  |  |  |
| 1 | Vvi-Vitvi05g04088\_t001 |  |  |  |  |  | \| \| |  |  |  |  |  |  |
| 1 | Vvi-Vitvi05g01849\_t001 |  |  |  |  |  | \| \| |  |  |  |  |  |  |
| 1 | Vvi-Vitvi05g01850\_t001 |  |  |  |  |  | \| \| |  |  |  |  |  |  |
| 1 | Vvi-Vitvi05g01851\_t001 |  |  |  |  |  | \| \| |  |  |  |  |  |  |
| 1 | Vvi-Vitvi05g01852\_t001 |  |  |  |  |  | \| \| |  |  |  |  |  |  |
| 1 | Vvi-Vitvi05g00333\_t001 |  |  |  |  |  | \| \| |  |  |  |  |  |  |
| 1 | Vvi-Vitvi05g00334\_t001 |  |  |  |  |  | Atr-ERN16947 |  |  |  |  |  |  |
| 1 | Vvi-Vitvi05g00335\_t001 |  |  |  |  |  | Atr-ERN16939 |  |  |  |  |  |  |
| 1 | Vvi-Vitvi05g00337\_t001 |  |  |  |  |  | Atr-ERN16937 |  |  |  |  |  |  |
| 1 | Vvi-Vitvi05g00338\_t001 |  |  |  |  |  | Atr-ERN16934 |  |  |  |  |  |  |
| 1 | Vvi-Vitvi05g04089\_t001 |  |  |  |  |  | \| \| |  |  |  |  |  |  |
| 1 | Vvi-Vitvi05g00339\_t001 |  |  |  |  |  | Atr-ERN16933 |  |  |  |  |  |  |
| 1 | Vvi-Vitvi05g00340\_t001 |  |  |  |  |  | \| \| |  |  |  |  |  |  |
| 1 | Vvi-Vitvi05g00341\_t001 |  |  |  |  |  | Atr-ERN16928 |  |  |  |  |  |  |
| 1 | Vvi-Vitvi05g00342\_t001 |  |  |  |  |  | \| \| |  |  |  |  |  |  |
| 1 | Vvi-Vitvi05g00343\_t001 |  |  |  |  |  | Atr-ERN16924 |  |  |  |  |  |  |
| 1 | Vvi-Vitvi05g00344\_t001 |  |  |  |  |  | \| \| |  |  |  |  |  |  |
| 1 | Vvi-Vitvi05g00345\_t001 |  |  |  |  |  | \| \| |  |  |  |  |  |  |
| 1 | Vvi-Vitvi05g00346\_t001 |  |  |  |  |  | Atr-ERN16918 |  |  |  |  |  |  |
| 1 | Vvi-Vitvi05g01856\_t001 |  |  |  |  |  | \| \| |  |  |  |  |  |  |
| 1 | Vvi-Vitvi05g00347\_t004 |  |  |  |  |  | \| \| |  |  |  |  |  |  |
| 1 | Vvi-Vitvi05g00348\_t001 |  |  |  |  |  | Atr-ERN16909 |  |  |  |  |  |  |
| 2 | Vvi-Vitvi05g01857\_t001 |  | Atr-ERN16881 |  |  |  | \| \| |  |  |  |  |  |  |
| 2 | Vvi-Vitvi05g00349\_t001 |  | Atr-ERN16883 |  |  |  | \| \| |  |  |  |  |  |  |
| 2 | Vvi-Vitvi05g01858\_t001 |  | \| \| |  |  |  | \| \| |  |  |  |  |  |  |
| 2 | Vvi-Vitvi05g00350\_t001 |  | Atr-ERN16902 |  |  |  | Atr-ERN16902 |  |  |  |  |  |  |
| 2 | Vvi-Vitvi05g00351\_t001 |  | Atr-ERN16913 |  |  |  | \| \| |  |  |  |  |  |  |
| 2 | Vvi-Vitvi05g00352\_t001 |  | Atr-ERN16929 |  |  |  | \| \| |  |  |  |  |  |  |
| 2 | Vvi-Vitvi05g00353\_t001 |  | \| \| |  |  |  | \| \| |  |  |  |  |  |  |
| 2 | Vvi-Vitvi05g04090\_t001 |  | \| \| |  |  |  | \| \| |  |  |  |  |  |  |
| 2 | Vvi-Vitvi05g04091\_t001 |  | \| \| |  |  |  | \| \| |  |  |  |  |  |  |
| 2 | Vvi-Vitvi05g00354\_t001 |  | Atr-ERN16930 |  |  |  | \| \| |  |  |  |  |  |  |
| 2 | Vvi-Vitvi05g00355\_t001 |  | Atr-ERN16931 |  |  |  | \| \| |  |  |  |  |  |  |
| 1 | Vvi-Vitvi05g01860\_t001 |  |  |  |  |  | \| \| |  |  |  |  |  |  |
| 1 | Vvi-Vitvi05g00357\_t001 |  |  |  |  |  | Atr-ERN16877 |  |  |  |  |  |  |
| 1 | Vvi-Vitvi05g01861\_t001 |  |  |  |  |  | \| \| |  |  |  |  |  |  |
| 1 | Vvi-Vitvi05g01862\_t002 |  |  |  |  |  | Atr-ERN16876 |  |  |  |  |  |  |
| 1 | Vvi-Vitvi05g00358\_t001 |  |  |  |  |  | Atr-ERN16875 |  |  |  |  |  |  |
| 1 | Vvi-Vitvi05g00359\_t001 |  |  |  |  |  | Atr-ERN16872 |  |  |  |  |  |  |
| 1 | Vvi-Vitvi05g00360\_t003 |  |  |  |  |  | Atr-ERN16870 |  |  |  |  |  |  |
| 1 | Vvi-Vitvi05g00362\_t001 |  |  |  |  |  | Atr-ERN16869 |  |  |  |  |  |  |
| 1 | Vvi-Vitvi05g04092\_t001 |  |  |  |  |  | \| \| |  |  |  |  |  |  |
| 1 | Vvi-Vitvi05g00363\_t001 |  |  |  |  |  | Atr-ERN16867 |  |  |  |  |  |  |
| 1 | Vvi-Vitvi05g01864\_t003 |  |  |  |  |  | \| \| |  |  |  |  |  |  |
| 1 | Vvi-Vitvi05g00364\_t001 |  |  |  |  |  | Atr-ERN16857 |  |  |  |  |  |  |
| 1 | Vvi-Vitvi05g00365\_t001 |  |  |  |  |  | Atr-ERN16855 |  |  |  |  |  |  |
| 1 | Vvi-Vitvi05g04093\_t001 |  |  |  |  |  | \| \| |  |  |  |  |  |  |
| 1 | Vvi-Vitvi05g00366\_t001 |  |  |  |  |  | Atr-ERN16853 |  |  |  |  |  |  |
| 1 | Vvi-Vitvi05g01865\_t001 |  |  |  |  |  | \| \| |  |  |  |  |  |  |
| 1 | Vvi-Vitvi05g00367\_t001 |  |  |  |  |  | Atr-ERN16852 |  |  |  |  |  |  |
| 1 | Vvi-Vitvi05g00368\_t002 |  |  |  |  |  | \| \| |  |  |  |  |  |  |
| 1 | Vvi-Vitvi05g00369\_t001 |  |  |  |  |  | \| \| |  |  |  |  |  |  |
| 1 | Vvi-Vitvi05g04094\_t001 |  |  |  |  |  | \| \| |  |  |  |  |  |  |
| 1 | Vvi-Vitvi05g00370\_t001 |  |  |  |  |  | Atr-ERN16844 |  |  |  |  |  |  |
| 1 | Vvi-Vitvi05g00371\_t001 |  |  |  |  |  | \| \| |  |  |  |  |  |  |
| 1 | Vvi-Vitvi05g01867\_t001 |  |  |  |  |  | \| \| |  |  |  |  |  |  |
| 1 | Vvi-Vitvi05g00372\_t001 |  |  |  |  |  | Atr-ERN16837 |  |  |  |  |  |  |
| 1 | Vvi-Vitvi05g04095\_t001 |  |  |  |  |  | \| \| |  |  |  |  |  |  |
| 1 | Vvi-Vitvi05g00374\_t002 |  |  |  |  |  | \| \| |  |  |  |  |  |  |
| 1 | Vvi-Vitvi05g00376\_t001 |  |  |  |  |  | \| \| |  |  |  |  |  |  |
| 1 | Vvi-Vitvi05g00377\_t001 |  |  |  |  |  | \| \| |  |  |  |  |  |  |
| 1 | Vvi-Vitvi05g04096\_t001 |  |  |  |  |  | \| \| |  |  |  |  |  |  |
| 1 | Vvi-Vitvi05g00380\_t001 |  |  |  |  |  | \| \| |  |  |  |  |  |  |
| 1 | Vvi-Vitvi05g00381\_t003 |  |  |  |  |  | \| \| |  |  |  |  |  |  |
| 1 | Vvi-Vitvi05g00382\_t001 |  |  |  |  |  | \| \| |  |  |  |  |  |  |
| 2 | Vvi-Vitvi05g00383\_t001 |  | Atr-ERN07494 |  |  |  | \| \| |  |  |  |  |  |  |
| 2 | Vvi-Vitvi05g00384\_t001 |  | Atr-ERN07492 |  |  |  | \| \| |  |  |  |  |  |  |
| 2 | Vvi-Vitvi05g01868\_t001 |  | Atr-ERN07488 |  |  |  | \| \| |  |  |  |  |  |  |
| 2 | Vvi-Vitvi05g01869\_t001 |  | Atr-ERN07481 |  |  |  | \| \| |  |  |  |  |  |  |
| 2 | Vvi-Vitvi05g01870\_t001 |  | \| \| |  |  |  | \| \| |  |  |  |  |  |  |
| 2 | Vvi-Vitvi05g01871\_t001 |  | Atr-ERN07479 |  |  |  | \| \| |  |  |  |  |  |  |
| 2 | Vvi-Vitvi05g00385\_t001 |  | Atr-ERN07475 |  |  |  | \| \| |  |  |  |  |  |  |
| 2 | Vvi-Vitvi05g00386\_t001 |  | Atr-ERN07471 |  |  |  | \| \| |  |  |  |  |  |  |
| 2 | Vvi-Vitvi05g01872\_t001 |  | Atr-ERN07468 |  |  |  | \| \| |  |  |  |  |  |  |
| 2 | Vvi-Vitvi05g00387\_t001 |  | Atr-ERN12449 |  |  |  | \| \| |  |  |  |  |  |  |
| 2 | Vvi-Vitvi05g00388\_t002 |  | Atr-ERN12448 |  |  |  | \| \| |  |  |  |  |  |  |
| 2 | Vvi-Vitvi05g00389\_t001 |  | Atr-ERN12440 |  |  |  | \| \| |  |  |  |  |  |  |
| 2 | Vvi-Vitvi05g00390\_t001 |  | Atr-ERN12438 |  |  |  | \| \| |  |  |  |  |  |  |
| 2 | Vvi-Vitvi05g00391\_t001 |  | Atr-ERN12434 |  |  |  | \| \| |  |  |  |  |  |  |
| 2 | Vvi-Vitvi05g04097\_t001 |  | \| \| |  |  |  | \| \| |  |  |  |  |  |  |
| 2 | Vvi-Vitvi05g00392\_t001 |  | Atr-ERN12433 |  |  |  | Atr-ERN16831 |  |  |  |  |  |  |
| 2 | Vvi-Vitvi05g04098\_t001 |  | \| \| |  |  |  | \| \| |  |  |  |  |  |  |
| 2 | Vvi-Vitvi05g00393\_t001 |  | Atr-ERN12431 |  |  |  | \| \| |  |  |  |  |  |  |
| 2 | Vvi-Vitvi05g00394\_t001 |  | Atr-ERN12427 |  |  |  | Atr-ERN16830 |  |  |  |  |  |  |
| 1 | Vvi-Vitvi05g00395\_t001 |  | Atr-ERN12424 |  |  |  |  |  |  |  |  |  |  |
| 1 | Vvi-Vitvi05g00396\_t001 |  | \| \| |  |  |  |  |  |  |  |  |  |  |
| 1 | Vvi-Vitvi05g00397\_t001 |  | \| \| |  |  |  |  |  |  |  |  |  |  |
| 1 | Vvi-Vitvi05g00398\_t001 |  | Atr-ERN12418 |  |  |  |  |  |  |  |  |  |  |
| 1 | Vvi-Vitvi05g00399\_t001 |  | \| \| |  |  |  |  |  |  |  |  |  |  |
| 1 | Vvi-Vitvi05g04099\_t001 |  | \| \| |  |  |  |  |  |  |  |  |  |  |
| 1 | Vvi-Vitvi05g00400\_t001 |  | \| \| |  |  |  |  |  |  |  |  |  |  |
| 1 | Vvi-Vitvi05g01873\_t001 |  | \| \| |  |  |  |  |  |  |  |  |  |  |
| 1 | Vvi-Vitvi05g00401\_t001 |  | Atr-ERN12399 |  |  |  |  |  |  |  |  |  |  |
| 1 | Vvi-Vitvi05g00402\_t001 |  | Atr-ERN12398 |  |  |  |  |  |  |  |  |  |  |
| 1 | Vvi-Vitvi05g00403\_t001 |  | Atr-ERN12396 |  |  |  |  |  |  |  |  |  |  |
| 1 | Vvi-Vitvi05g00404\_t003 |  | Atr-ERN12395 |  |  |  |  |  |  |  |  |  |  |
| 1 | Vvi-Vitvi05g00406\_t001 |  | Atr-ERN12392 |  |  |  |  |  |  |  |  |  |  |
| 1 | Vvi-Vitvi05g00407\_t001 |  | \| \| |  |  |  |  |  |  |  |  |  |  |
| 1 | Vvi-Vitvi05g00408\_t001 |  | Atr-ERN12389 |  |  |  |  |  |  |  |  |  |  |
| 1 | Vvi-Vitvi05g00409\_t001 |  | Atr-ERN12388 |  |  |  |  |  |  |  |  |  |  |
| 1 | Vvi-Vitvi05g00410\_t001 |  | Atr-ERN12385 |  |  |  |  |  |  |  |  |  |  |
| 1 | Vvi-Vitvi05g00411\_t001 |  | \| \| |  |  |  |  |  |  |  |  |  |  |
| 1 | Vvi-Vitvi05g00412\_t001 |  | Atr-ERN12381 |  |  |  |  |  |  |  |  |  |  |
| 1 | Vvi-Vitvi05g00413\_t001 |  | Atr-ERN12378 |  |  |  |  |  |  |  |  |  |  |
| 1 | Vvi-Vitvi05g00416\_t001 |  | \| \| |  |  |  |  |  |  |  |  |  |  |
| 1 | Vvi-Vitvi05g00418\_t001 |  | Atr-ERN12374 |  |  |  |  |  |  |  |  |  |  |
| 1 | Vvi-Vitvi05g00419\_t001 |  | Atr-ERN12372 |  |  |  |  |  |  |  |  |  |  |
| 1 | Vvi-Vitvi05g00420\_t001 |  | Atr-ERN12365 |  |  |  |  |  |  |  |  |  |  |
| 1 | Vvi-Vitvi05g01874\_t001 |  | Atr-ERN12363 |  |  |  |  |  |  |  |  |  |  |
| 1 | Vvi-Vitvi05g00421\_t001 |  | Atr-ERN12361 |  |  |  |  |  |  |  |  |  |  |
| 1 | Vvi-Vitvi05g04100\_t001 |  | \| \| |  |  |  |  |  |  |  |  |  |  |
| 1 | Vvi-Vitvi05g00422\_t001 |  | \| \| |  |  |  |  |  |  |  |  |  |  |
| 1 | Vvi-Vitvi05g00423\_t001 |  | Atr-ERN12359 |  |  |  |  |  |  |  |  |  |  |
| 1 | Vvi-Vitvi05g01875\_t001 |  | Atr-ERN12357 |  |  |  |  |  |  |  |  |  |  |
| 1 | Vvi-Vitvi05g00424\_t001 |  | Atr-ERN12356 |  |  |  |  |  |  |  |  |  |  |
| 1 | Vvi-Vitvi05g00425\_t002 |  | Atr-ERN12352 |  |  |  |  |  |  |  |  |  |  |
| 1 | Vvi-Vitvi05g00426\_t001 |  | Atr-ERN12351 |  |  |  |  |  |  |  |  |  |  |
| 1 | Vvi-Vitvi05g00427\_t001 |  | Atr-ERN12350 |  |  |  |  |  |  |  |  |  |  |
| 1 | Vvi-Vitvi05g00429\_t001 |  | Atr-ERN12348 |  |  |  |  |  |  |  |  |  |  |
| 1 | Vvi-Vitvi05g04101\_t001 |  | \| \| |  |  |  |  |  |  |  |  |  |  |
| 1 | Vvi-Vitvi05g00430\_t001 |  | Atr-ERN12344 |  |  |  |  |  |  |  |  |  |  |
| 1 | Vvi-Vitvi05g00431\_t001 |  | \| \| |  |  |  |  |  |  |  |  |  |  |
| 1 | Vvi-Vitvi05g00432\_t001 |  | \| \| |  |  |  |  |  |  |  |  |  |  |
| 1 | Vvi-Vitvi05g00433\_t001 |  | Atr-ERN12334 |  |  |  |  |  |  |  |  |  |  |
| 1 | Vvi-Vitvi05g04102\_t001 |  | \| \| |  |  |  |  |  |  |  |  |  |  |
| 1 | Vvi-Vitvi05g00434\_t001 |  | Atr-ERN12332 |  |  |  |  |  |  |  |  |  |  |
| 1 | Vvi-Vitvi05g00435\_t001 |  | \| \| |  |  |  |  |  |  |  |  |  |  |
| 1 | Vvi-Vitvi05g00436\_t001 |  | \| \| |  |  |  |  |  |  |  |  |  |  |
| 1 | Vvi-Vitvi05g00437\_t001 |  | \| \| |  |  |  |  |  |  |  |  |  |  |
| 1 | Vvi-Vitvi05g00438\_t001 |  | Atr-ERN12325 |  |  |  |  |  |  |  |  |  |  |
| 1 | Vvi-Vitvi05g01877\_t001 |  | \| \| |  |  |  |  |  |  |  |  |  |  |
| 1 | Vvi-Vitvi05g01878\_t002 |  | \| \| |  |  |  |  |  |  |  |  |  |  |
| 1 | Vvi-Vitvi05g00439\_t001 |  | Atr-ERN12311 |  |  |  |  |  |  |  |  |  |  |
| 1 | Vvi-Vitvi05g00440\_t001 |  | \| \| |  |  |  |  |  |  |  |  |  |  |
| 1 | Vvi-Vitvi05g00441\_t001 |  | \| \| |  |  |  |  |  |  |  |  |  |  |
| 1 | Vvi-Vitvi05g00442\_t001 |  | Atr-ERN12306 |  |  |  |  |  |  |  |  |  |  |
| 1 | Vvi-Vitvi05g01879\_t001 |  | Atr-ERN12301 |  |  |  |  |  |  |  |  |  |  |
| 0 | Vvi-Vitvi05g00443\_t001 |  |  |  |  |  |  |  |  |  |  |  |  |
| 0 | Vvi-Vitvi05g00444\_t001 |  |  |  |  |  |  |  |  |  |  |  |  |
| 0 | Vvi-Vitvi05g04103\_t001 |  |  |  |  |  |  |  |  |  |  |  |  |
| 1 | Vvi-Vitvi05g00448\_t001 |  | Atr-ERN12697 |  |  |  |  |  |  |  |  |  |  |
| 1 | Vvi-Vitvi05g00449\_t001 |  | \| \| |  |  |  |  |  |  |  |  |  |  |
| 1 | Vvi-Vitvi05g04104\_t001 |  | \| \| |  |  |  |  |  |  |  |  |  |  |
| 1 | Vvi-Vitvi05g00451\_t001 |  | \| \| |  |  |  |  |  |  |  |  |  |  |
| 1 | Vvi-Vitvi05g00452\_t001 |  | Atr-ERN12683 |  |  |  |  |  |  |  |  |  |  |
| 1 | Vvi-Vitvi05g00453\_t001 |  | Atr-ERN12675 |  |  |  |  |  |  |  |  |  |  |
| 1 | Vvi-Vitvi05g00454\_t001 |  | \| \| |  |  |  |  |  |  |  |  |  |  |
| 1 | Vvi-Vitvi05g00455\_t001 |  | \| \| |  |  |  |  |  |  |  |  |  |  |
| 1 | Vvi-Vitvi05g00456\_t001 |  | \| \| |  |  |  |  |  |  |  |  |  |  |
| 1 | Vvi-Vitvi05g00457\_t001 |  | \| \| |  |  |  |  |  |  |  |  |  |  |
| 1 | Vvi-Vitvi05g00458\_t002 |  | Atr-ERN12670 |  |  |  |  |  |  |  |  |  |  |
| 1 | Vvi-Vitvi05g04105\_t001 |  | \| \| |  |  |  |  |  |  |  |  |  |  |
| 1 | Vvi-Vitvi05g00459\_t001 |  | Atr-ERN12669 |  |  |  |  |  |  |  |  |  |  |
| 1 | Vvi-Vitvi05g00460\_t001 |  | Atr-ERN12668 |  |  |  |  |  |  |  |  |  |  |
| 1 | Vvi-Vitvi05g00461\_t001 |  | Atr-ERN12666 |  |  |  |  |  |  |  |  |  |  |
| 1 | Vvi-Vitvi05g00462\_t008 |  | Atr-ERN12665 |  |  |  |  |  |  |  |  |  |  |
| 1 | Vvi-Vitvi05g00463\_t001 |  | Atr-ERN12662 |  |  |  |  |  |  |  |  |  |  |
| 1 | Vvi-Vitvi05g00464\_t001 |  | Atr-ERN00844 |  |  |  |  |  |  |  |  |  |  |
| 1 | Vvi-Vitvi05g00465\_t001 |  | \| \| |  |  |  |  |  |  |  |  |  |  |
| 1 | Vvi-Vitvi05g00466\_t001 |  | \| \| |  |  |  |  |  |  |  |  |  |  |
| 1 | Vvi-Vitvi05g00467\_t001 |  | Atr-ERN00840 |  |  |  |  |  |  |  |  |  |  |
| 1 | Vvi-Vitvi05g00468\_t001 |  | \| \| |  |  |  |  |  |  |  |  |  |  |
| 1 | Vvi-Vitvi05g04106\_t001 |  | \| \| |  |  |  |  |  |  |  |  |  |  |
| 1 | Vvi-Vitvi05g00469\_t001 |  | Atr-ERN00832 |  |  |  |  |  |  |  |  |  |  |
| 1 | Vvi-Vitvi05g00470\_t001 |  | Atr-ERN00826 |  |  |  |  |  |  |  |  |  |  |
| 1 | Vvi-Vitvi05g04107\_t001 |  | \| \| |  |  |  |  |  |  |  |  |  |  |
| 1 | Vvi-Vitvi05g00471\_t001 |  | \| \| |  |  |  |  |  |  |  |  |  |  |
| 1 | Vvi-Vitvi05g00472\_t001 |  | \| \| |  |  |  |  |  |  |  |  |  |  |
| 1 | Vvi-Vitvi05g00474\_t001 |  | Atr-ERN00816 |  |  |  |  |  |  |  |  |  |  |
| 1 | Vvi-Vitvi05g00475\_t001 |  | Atr-ERN00815 |  |  |  |  |  |  |  |  |  |  |
| 1 | Vvi-Vitvi05g00476\_t001 |  | Atr-ERN00812 |  |  |  |  |  |  |  |  |  |  |
| 1 | Vvi-Vitvi05g01881\_t001 |  | \| \| |  |  |  |  |  |  |  |  |  |  |
| 1 | Vvi-Vitvi05g04108\_t001 |  | \| \| |  |  |  |  |  |  |  |  |  |  |
| 1 | Vvi-Vitvi05g00477\_t001 |  | \| \| |  |  |  |  |  |  |  |  |  |  |
| 1 | Vvi-Vitvi05g00478\_t001 |  | \| \| |  |  |  |  |  |  |  |  |  |  |
| 1 | Vvi-Vitvi05g04109\_t001 |  | \| \| |  |  |  |  |  |  |  |  |  |  |
| 1 | Vvi-Vitvi05g00479\_t001 |  | \| \| |  |  |  |  |  |  |  |  |  |  |
| 1 | Vvi-Vitvi05g00481\_t001 |  | \| \| |  |  |  |  |  |  |  |  |  |  |
| 1 | Vvi-Vitvi05g00482\_t001 |  | \| \| |  |  |  |  |  |  |  |  |  |  |
| 1 | Vvi-Vitvi05g00483\_t001 |  | \| \| |  |  |  |  |  |  |  |  |  |  |
| 1 | Vvi-Vitvi05g00484\_t001 |  | \| \| |  |  |  |  |  |  |  |  |  |  |
| 1 | Vvi-Vitvi05g04110\_t001 |  | \| \| |  |  |  |  |  |  |  |  |  |  |
| 1 | Vvi-Vitvi05g04111\_t001 |  | \| \| |  |  |  |  |  |  |  |  |  |  |
| 1 | Vvi-Vitvi05g00485\_t001 |  | \| \| |  |  |  |  |  |  |  |  |  |  |
| 1 | Vvi-Vitvi05g00486\_t001 |  | Atr-ERN00804 |  |  |  |  |  |  |  |  |  |  |
| 1 | Vvi-Vitvi05g01885\_t001 |  | Atr-ERN00802 |  |  |  |  |  |  |  |  |  |  |
| 0 | Vvi-Vitvi05g00487\_t001 |  |  |  |  |  |  |  |  |  |  |  |  |
| 0 | Vvi-Vitvi05g01886\_t001 |  |  |  |  |  |  |  |  |  |  |  |  |
| 0 | Vvi-Vitvi05g00490\_t001 |  |  |  |  |  |  |  |  |  |  |  |  |
| 0 | Vvi-Vitvi05g04112\_t001 |  |  |  |  |  |  |  |  |  |  |  |  |
| 0 | Vvi-Vitvi05g00492\_t001 |  |  |  |  |  |  |  |  |  |  |  |  |
| 0 | Vvi-Vitvi05g01887\_t001 |  |  |  |  |  |  |  |  |  |  |  |  |
| 0 | Vvi-Vitvi05g04113\_t001 |  |  |  |  |  |  |  |  |  |  |  |  |
| 0 | Vvi-Vitvi05g04114\_t001 |  |  |  |  |  |  |  |  |  |  |  |  |
| 0 | Vvi-Vitvi05g00495\_t001 |  |  |  |  |  |  |  |  |  |  |  |  |
| 0 | Vvi-Vitvi05g04115\_t001 |  |  |  |  |  |  |  |  |  |  |  |  |
| 1 | Vvi-Vitvi05g00496\_t001 |  | Atr-ERM93519 |  |  |  |  |  |  |  |  |  |  |
| 1 | Vvi-Vitvi05g04116\_t001 |  | \| \| |  |  |  |  |  |  |  |  |  |  |
| 1 | Vvi-Vitvi05g00497\_t001 |  | Atr-ERM93515 |  |  |  |  |  |  |  |  |  |  |
| 1 | Vvi-Vitvi05g00498\_t001 |  | \| \| |  |  |  |  |  |  |  |  |  |  |
| 1 | Vvi-Vitvi05g04117\_t001 |  | \| \| |  |  |  |  |  |  |  |  |  |  |
| 1 | Vvi-Vitvi05g04118\_t001 |  | \| \| |  |  |  |  |  |  |  |  |  |  |
| 1 | Vvi-Vitvi05g04119\_t001 |  | \| \| |  |  |  |  |  |  |  |  |  |  |
| 1 | Vvi-Vitvi05g00500\_t001 |  | \| \| |  |  |  |  |  |  |  |  |  |  |
| 1 | Vvi-Vitvi05g00503\_t001 |  | Atr-ERM93512 |  |  |  |  |  |  |  |  |  |  |
| 1 | Vvi-Vitvi05g00504\_t001 |  | \| \| |  |  |  |  |  |  |  |  |  |  |
| 1 | Vvi-Vitvi05g00505\_t001 |  | Atr-ERM93511 |  |  |  |  |  |  |  |  |  |  |
| 1 | Vvi-Vitvi05g00506\_t002 |  | \| \| |  |  |  |  |  |  |  |  |  |  |
| 1 | Vvi-Vitvi05g00508\_t001 |  | \| \| |  |  |  |  |  |  |  |  |  |  |
| 1 | Vvi-Vitvi05g00509\_t001 |  | \| \| |  |  |  |  |  |  |  |  |  |  |
| 1 | Vvi-Vitvi05g00510\_t001 |  | Atr-ERM93506 |  |  |  |  |  |  |  |  |  |  |
| 1 | Vvi-Vitvi05g00512\_t001 |  | \| \| |  |  |  |  |  |  |  |  |  |  |
| 1 | Vvi-Vitvi05g00513\_t001 |  | \| \| |  |  |  |  |  |  |  |  |  |  |
| 1 | Vvi-Vitvi05g01890\_t001 |  | Atr-ERM93497 |  |  |  |  |  |  |  |  |  |  |
| 1 | Vvi-Vitvi05g04120\_t001 |  | Atr-ERM93496 |  |  |  |  |  |  |  |  |  |  |
| 1 | Vvi-Vitvi05g00517\_t001 |  | Atr-ERM93495 |  |  |  |  |  |  |  |  |  |  |
| 1 | Vvi-Vitvi05g01891\_t001 |  | Atr-ERM93491 |  |  |  |  |  |  |  |  |  |  |
| 1 | Vvi-Vitvi05g00518\_t001 |  | \| \| |  |  |  |  |  |  |  |  |  |  |
| 1 | Vvi-Vitvi05g00519\_t001 |  | Atr-ERM93488 |  |  |  |  |  |  |  |  |  |  |
| 1 | Vvi-Vitvi05g04121\_t001 |  | \| \| |  |  |  |  |  |  |  |  |  |  |
| 1 | Vvi-Vitvi05g00520\_t001 |  | Atr-ERM93487 |  |  |  |  |  |  |  |  |  |  |
| 1 | Vvi-Vitvi05g00521\_t001 |  | Atr-ERM93486 |  |  |  |  |  |  |  |  |  |  |
| 1 | Vvi-Vitvi05g04122\_t001 |  | Atr-ERM93485 |  |  |  |  |  |  |  |  |  |  |
| 0 | Vvi-Vitvi05g00523\_t001 |  |  |  |  |  |  |  |  |  |  |  |  |
| 0 | Vvi-Vitvi05g00524\_t001 |  |  |  |  |  |  |  |  |  |  |  |  |
| 0 | Vvi-Vitvi05g04123\_t001 |  |  |  |  |  |  |  |  |  |  |  |  |
| 0 | Vvi-Vitvi05g01892\_t001 |  |  |  |  |  |  |  |  |  |  |  |  |
| 0 | Vvi-Vitvi05g01893\_t001 |  |  |  |  |  |  |  |  |  |  |  |  |
| 0 | Vvi-Vitvi05g04124\_t001 |  |  |  |  |  |  |  |  |  |  |  |  |
| 0 | Vvi-Vitvi05g04125\_t001 |  |  |  |  |  |  |  |  |  |  |  |  |
| 1 | Vvi-Vitvi05g00526\_t001 |  | Atr-ERN01589 |  |  |  |  |  |  |  |  |  |  |
| 1 | Vvi-Vitvi05g00527\_t002 |  | Atr-ERN01588 |  |  |  |  |  |  |  |  |  |  |
| 1 | Vvi-Vitvi05g00528\_t001 |  | Atr-ERN01587 |  |  |  |  |  |  |  |  |  |  |
| 1 | Vvi-Vitvi05g00529\_t001 |  | \| \| |  |  |  |  |  |  |  |  |  |  |
| 1 | Vvi-Vitvi05g01894\_t001 |  | \| \| |  |  |  |  |  |  |  |  |  |  |
| 1 | Vvi-Vitvi05g00530\_t001 |  | Atr-ERN01585 |  |  |  |  |  |  |  |  |  |  |
| 1 | Vvi-Vitvi05g04126\_t001 |  | \| \| |  |  |  |  |  |  |  |  |  |  |
| 1 | Vvi-Vitvi05g04127\_t001 |  | \| \| |  |  |  |  |  |  |  |  |  |  |
| 1 | Vvi-Vitvi05g00532\_t001 |  | \| \| |  |  |  |  |  |  |  |  |  |  |
| 1 | Vvi-Vitvi05g04128\_t001 |  | \| \| |  |  |  |  |  |  |  |  |  |  |
| 1 | Vvi-Vitvi05g00533\_t001 |  | Atr-ERN01575 |  |  |  |  |  |  |  |  |  |  |
| 1 | Vvi-Vitvi05g00534\_t001 |  | Atr-ERN01573 |  |  |  |  |  |  |  |  |  |  |
| 1 | Vvi-Vitvi05g00535\_t001 |  | Atr-ERN01571 |  |  |  |  |  |  |  |  |  |  |
| 1 | Vvi-Vitvi05g04129\_t001 |  | \| \| |  |  |  |  |  |  |  |  |  |  |
| 1 | Vvi-Vitvi05g01895\_t001 |  | \| \| |  |  |  |  |  |  |  |  |  |  |
| 1 | Vvi-Vitvi05g04130\_t001 |  | \| \| |  |  |  |  |  |  |  |  |  |  |
| 1 | Vvi-Vitvi05g01896\_t001 |  | \| \| |  |  |  |  |  |  |  |  |  |  |
| 1 | Vvi-Vitvi05g00537\_t002 |  | Atr-ERN01570 |  |  |  |  |  |  |  |  |  |  |
| 1 | Vvi-Vitvi05g00538\_t001 |  | \| \| |  |  |  |  |  |  |  |  |  |  |
| 1 | Vvi-Vitvi05g00539\_t003 |  | Atr-ERN01568 |  |  |  |  |  |  |  |  |  |  |
| 1 | Vvi-Vitvi05g00540\_t001 |  | Atr-ERN01567 |  |  |  |  |  |  |  |  |  |  |
| 1 | Vvi-Vitvi05g04131\_t001 |  | \| \| |  |  |  |  |  |  |  |  |  |  |
| 1 | Vvi-Vitvi05g00541\_t001 |  | Atr-ERN01561 |  |  |  |  |  |  |  |  |  |  |
| 1 | Vvi-Vitvi05g00542\_t001 |  | Atr-ERN01560 |  |  |  |  |  |  |  |  |  |  |
| 1 | Vvi-Vitvi05g00543\_t001 |  | Atr-ERN01559 |  |  |  |  |  |  |  |  |  |  |
| 1 | Vvi-Vitvi05g00544\_t001 |  | Atr-ERN01556 |  |  |  |  |  |  |  |  |  |  |
| 1 | Vvi-Vitvi05g00545\_t001 |  | Atr-ERN01555 |  |  |  |  |  |  |  |  |  |  |
| 1 | Vvi-Vitvi05g00546\_t001 |  | Atr-ERN01554 |  |  |  |  |  |  |  |  |  |  |
| 1 | Vvi-Vitvi05g04132\_t001 |  | \| \| |  |  |  |  |  |  |  |  |  |  |
| 1 | Vvi-Vitvi05g00548\_t001 |  | \| \| |  |  |  |  |  |  |  |  |  |  |
| 1 | Vvi-Vitvi05g04133\_t001 |  | \| \| |  |  |  |  |  |  |  |  |  |  |
| 1 | Vvi-Vitvi05g00549\_t001 |  | Atr-ERN01544 |  |  |  |  |  |  |  |  |  |  |
| 1 | Vvi-Vitvi05g00551\_t001 |  | Atr-ERN01540 |  |  |  |  |  |  |  |  |  |  |
| 1 | Vvi-Vitvi05g00553\_t001 |  | Atr-ERN01538 |  |  |  |  |  |  |  |  |  |  |
| 1 | Vvi-Vitvi05g00554\_t001 |  | Atr-ERN01530 |  |  |  |  |  |  |  |  |  |  |
| 1 | Vvi-Vitvi05g00555\_t001 |  | Atr-ERN01529 |  |  |  |  |  |  |  |  |  |  |
| 1 | Vvi-Vitvi05g00556\_t001 |  | Atr-ERN01527 |  |  |  |  |  |  |  |  |  |  |
| 1 | Vvi-Vitvi05g00557\_t001 |  | Atr-ERN01524 |  |  |  |  |  |  |  |  |  |  |
| 1 | Vvi-Vitvi05g04134\_t001 |  | \| \| |  |  |  |  |  |  |  |  |  |  |
| 1 | Vvi-Vitvi05g00558\_t002 |  | Atr-ERN01521 |  |  |  |  |  |  |  |  |  |  |
| 1 | Vvi-Vitvi05g00559\_t002 |  | Atr-ERN01517 |  |  |  |  |  |  |  |  |  |  |
| 1 | Vvi-Vitvi05g00560\_t001 |  | \| \| |  |  |  |  |  |  |  |  |  |  |
| 1 | Vvi-Vitvi05g00561\_t001 |  | Atr-ERN01513 |  |  |  |  |  |  |  |  |  |  |
| 1 | Vvi-Vitvi05g04135\_t001 |  | \| \| |  |  |  |  |  |  |  |  |  |  |
| 1 | Vvi-Vitvi05g00562\_t001 |  | Atr-ERN01512 |  |  |  |  |  |  |  |  |  |  |
| 1 | Vvi-Vitvi05g00563\_t001 |  | Atr-ERN01509 |  |  |  |  |  |  |  |  |  |  |
| 1 | Vvi-Vitvi05g00564\_t002 |  | \| \| |  |  |  |  |  |  |  |  |  |  |
| 1 | Vvi-Vitvi05g00565\_t001 |  | Atr-ERN01506 |  |  |  |  |  |  |  |  |  |  |
| 1 | Vvi-Vitvi05g04136\_t001 |  | \| \| |  |  |  |  |  |  |  |  |  |  |
| 1 | Vvi-Vitvi05g00568\_t001 |  | Atr-ERN01504 |  |  |  |  |  |  |  |  |  |  |
| 1 | Vvi-Vitvi05g00569\_t001 |  | Atr-ERN01494 |  |  |  |  |  |  |  |  |  |  |
| 1 | Vvi-Vitvi05g00570\_t001 |  | Atr-ERN01492 |  |  |  |  |  |  |  |  |  |  |
| 1 | Vvi-Vitvi05g00573\_t001 |  | Atr-ERN01491 |  |  |  |  |  |  |  |  |  |  |
| 1 | Vvi-Vitvi05g00574\_t002 |  | Atr-ERN01490 |  |  |  |  |  |  |  |  |  |  |
| 1 | Vvi-Vitvi05g00575\_t001 |  | \| \| |  |  |  |  |  |  |  |  |  |  |
| 1 | Vvi-Vitvi05g00576\_t001 |  | Atr-ERN01489 |  |  |  |  |  |  |  |  |  |  |
| 0 | Vvi-Vitvi05g00577\_t001 |  |  |  |  |  |  |  |  |  |  |  |  |
| 0 | Vvi-Vitvi05g04137\_t001 |  |  |  |  |  |  |  |  |  |  |  |  |
| 0 | Vvi-Vitvi05g01900\_t001 |  |  |  |  |  |  |  |  |  |  |  |  |
| 0 | Vvi-Vitvi05g04138\_t001 |  |  |  |  |  |  |  |  |  |  |  |  |
| 0 | Vvi-Vitvi05g00579\_t001 |  |  |  |  |  |  |  |  |  |  |  |  |
| 0 | Vvi-Vitvi05g00580\_t001 |  |  |  |  |  |  |  |  |  |  |  |  |
| 0 | Vvi-Vitvi05g00582\_t001 |  |  |  |  |  |  |  |  |  |  |  |  |
| 0 | Vvi-Vitvi05g00583\_t001 |  |  |  |  |  |  |  |  |  |  |  |  |
| 0 | Vvi-Vitvi05g04139\_t001 |  |  |  |  |  |  |  |  |  |  |  |  |
| 0 | Vvi-Vitvi05g00584\_t001 |  |  |  |  |  |  |  |  |  |  |  |  |
| 0 | Vvi-Vitvi05g00585\_t001 |  |  |  |  |  |  |  |  |  |  |  |  |
| 0 | Vvi-Vitvi05g04140\_t001 |  |  |  |  |  |  |  |  |  |  |  |  |
| 0 | Vvi-Vitvi05g00588\_t001 |  |  |  |  |  |  |  |  |  |  |  |  |
| 0 | Vvi-Vitvi05g00589\_t001 |  |  |  |  |  |  |  |  |  |  |  |  |
| 0 | Vvi-Vitvi05g04141\_t001 |  |  |  |  |  |  |  |  |  |  |  |  |
| 0 | Vvi-Vitvi05g00590\_t001 |  |  |  |  |  |  |  |  |  |  |  |  |
| 0 | Vvi-Vitvi05g00592\_t001 |  |  |  |  |  |  |  |  |  |  |  |  |
| 0 | Vvi-Vitvi05g04142\_t001 |  |  |  |  |  |  |  |  |  |  |  |  |
| 0 | Vvi-Vitvi05g04143\_t001 |  |  |  |  |  |  |  |  |  |  |  |  |
| 0 | Vvi-Vitvi05g00597\_t001 |  |  |  |  |  |  |  |  |  |  |  |  |
| 0 | Vvi-Vitvi05g00598\_t001 |  |  |  |  |  |  |  |  |  |  |  |  |
| 0 | Vvi-Vitvi05g04144\_t001 |  |  |  |  |  |  |  |  |  |  |  |  |
| 0 | Vvi-Vitvi05g00599\_t001 |  |  |  |  |  |  |  |  |  |  |  |  |
| 0 | Vvi-Vitvi05g00600\_t001 |  |  |  |  |  |  |  |  |  |  |  |  |
| 0 | Vvi-Vitvi05g00601\_t001 |  |  |  |  |  |  |  |  |  |  |  |  |
| 0 | Vvi-Vitvi05g04145\_t001 |  |  |  |  |  |  |  |  |  |  |  |  |
| 0 | Vvi-Vitvi05g04146\_t001 |  |  |  |  |  |  |  |  |  |  |  |  |
| 0 | Vvi-Vitvi05g04147\_t001 |  |  |  |  |  |  |  |  |  |  |  |  |
| 0 | Vvi-Vitvi05g00604\_t002 |  |  |  |  |  |  |  |  |  |  |  |  |
| 1 | Vvi-Vitvi05g00605\_t001 |  | Atr-ERN01481 |  |  |  |  |  |  |  |  |  |  |
| 1 | Vvi-Vitvi05g00606\_t001 |  | \| \| |  |  |  |  |  |  |  |  |  |  |
| 1 | Vvi-Vitvi05g00607\_t001 |  | \| \| |  |  |  |  |  |  |  |  |  |  |
| 1 | Vvi-Vitvi05g04148\_t001 |  | \| \| |  |  |  |  |  |  |  |  |  |  |
| 1 | Vvi-Vitvi05g04149\_t001 |  | \| \| |  |  |  |  |  |  |  |  |  |  |
| 1 | Vvi-Vitvi05g00609\_t001 |  | Atr-ERN01477 |  |  |  |  |  |  |  |  |  |  |
| 1 | Vvi-Vitvi05g00611\_t001 |  | Atr-ERN01476 |  |  |  |  |  |  |  |  |  |  |
| 1 | Vvi-Vitvi05g00612\_t001 |  | Atr-ERN01472 |  |  |  |  |  |  |  |  |  |  |
| 1 | Vvi-Vitvi05g04150\_t001 |  | Atr-ERN01471 |  |  |  |  |  |  |  |  |  |  |
| 1 | Vvi-Vitvi05g00614\_t003 |  | \| \| |  |  |  |  |  |  |  |  |  |  |
| 1 | Vvi-Vitvi05g00615\_t002 |  | \| \| |  |  |  |  |  |  |  |  |  |  |
| 1 | Vvi-Vitvi05g00616\_t001 |  | \| \| |  |  |  |  |  |  |  |  |  |  |
| 1 | Vvi-Vitvi05g01904\_t001 |  | Atr-ERN01468 |  |  |  |  |  |  |  |  |  |  |
| 1 | Vvi-Vitvi05g00617\_t001 |  | Atr-ERN01467 |  |  |  |  |  |  |  |  |  |  |
| 1 | Vvi-Vitvi05g00618\_t001 |  | \| \| |  |  |  |  |  |  |  |  |  |  |
| 1 | Vvi-Vitvi05g00619\_t001 |  | Atr-ERN01461 |  |  |  |  |  |  |  |  |  |  |
| 1 | Vvi-Vitvi05g00620\_t001 |  | Atr-ERN01459 |  |  |  |  |  |  |  |  |  |  |
| 1 | Vvi-Vitvi05g00621\_t001 |  | Atr-ERN01454 |  |  |  |  |  |  |  |  |  |  |
| 1 | Vvi-Vitvi05g00623\_t001 |  | \| \| |  |  |  |  |  |  |  |  |  |  |
| 1 | Vvi-Vitvi05g00624\_t001 |  | Atr-ERN01450 |  |  |  |  |  |  |  |  |  |  |
| 1 | Vvi-Vitvi05g00625\_t001 |  | Atr-ERN01446 |  |  |  |  |  |  |  |  |  |  |
| 1 | Vvi-Vitvi05g04151\_t001 |  | \| \| |  |  |  |  |  |  |  |  |  |  |
| 1 | Vvi-Vitvi05g00626\_t001 |  | Atr-ERN01445 |  |  |  |  |  |  |  |  |  |  |
| 1 | Vvi-Vitvi05g00627\_t001 |  | Atr-ERN01444 |  |  |  |  |  |  |  |  |  |  |
| 1 | Vvi-Vitvi05g00628\_t001 |  | Atr-ERN01439 |  |  |  |  |  |  |  |  |  |  |
| 1 | Vvi-Vitvi05g00630\_t001 |  | Atr-ERN01436 |  |  |  |  |  |  |  |  |  |  |
| 1 | Vvi-Vitvi05g00631\_t001 |  | \| \| |  |  |  |  |  |  |  |  |  |  |
| 1 | Vvi-Vitvi05g04152\_t001 |  | \| \| |  |  |  |  |  |  |  |  |  |  |
| 1 | Vvi-Vitvi05g00632\_t001 |  | Atr-ERN01430 |  |  |  |  |  |  |  |  |  |  |
| 1 | Vvi-Vitvi05g01905\_t001 |  | \| \| |  |  |  |  |  |  |  |  |  |  |
| 1 | Vvi-Vitvi05g00633\_t002 |  | Atr-ERN01427 |  |  |  |  |  |  |  |  |  |  |
| 1 | Vvi-Vitvi05g00634\_t001 |  | Atr-ERN01426 |  |  |  |  |  |  |  |  |  |  |
| 1 | Vvi-Vitvi05g01906\_t001 |  | Atr-ERN01421 |  |  |  |  |  |  |  |  |  |  |
| 1 | Vvi-Vitvi05g04153\_t001 |  | \| \| |  |  |  |  |  |  |  |  |  |  |
| 1 | Vvi-Vitvi05g00637\_t001 |  | \| \| |  |  |  |  |  |  |  |  |  |  |
| 1 | Vvi-Vitvi05g00638\_t001.1.6037826e |  | Atr-ERN01420 |  |  |  |  |  |  |  |  |  |  |
| 1 | Vvi-Vitvi05g04154\_t001 |  | \| \| |  |  |  |  |  |  |  |  |  |  |
| 1 | Vvi-Vitvi05g00639\_t001 |  | Atr-ERN01418 |  |  |  |  |  |  |  |  |  |  |
| 1 | Vvi-Vitvi05g04155\_t001 |  | \| \| |  |  |  |  |  |  |  |  |  |  |
| 1 | Vvi-Vitvi05g00640\_t001 |  | Atr-ERN01416 |  |  |  |  |  |  |  |  |  |  |
| 1 | Vvi-Vitvi05g00641\_t001 |  | \| \| |  |  |  |  |  |  |  |  |  |  |
| 1 | Vvi-Vitvi05g00642\_t001 |  | Atr-ERN01411 |  |  |  |  |  |  |  |  |  |  |
| 1 | Vvi-Vitvi05g00643\_t001 |  | \| \| |  |  |  |  |  |  |  |  |  |  |
| 1 | Vvi-Vitvi05g00644\_t001 |  | \| \| |  |  |  |  |  |  |  |  |  |  |
| 1 | Vvi-Vitvi05g00648\_t001 |  | \| \| |  |  |  |  |  |  |  |  |  |  |
| 1 | Vvi-Vitvi05g00649\_t001 |  | \| \| |  |  |  |  |  |  |  |  |  |  |
| 1 | Vvi-Vitvi05g00650\_t001 |  | Atr-ERN01409 |  |  |  |  |  |  |  |  |  |  |
| 1 | Vvi-Vitvi05g00651\_t001 |  | Atr-ERN01408 |  |  |  |  |  |  |  |  |  |  |
| 1 | Vvi-Vitvi05g00652\_t001 |  | \| \| |  |  |  |  |  |  |  |  |  |  |
| 1 | Vvi-Vitvi05g00653\_t001 |  | \| \| |  |  |  |  |  |  |  |  |  |  |
| 1 | Vvi-Vitvi05g00654\_t001 |  | Atr-ERN01402 |  |  |  |  |  |  |  |  |  |  |
| 1 | Vvi-Vitvi05g00655\_t001 |  | \| \| |  |  |  |  |  |  |  |  |  |  |
| 1 | Vvi-Vitvi05g00656\_t001 |  | \| \| |  |  |  |  |  |  |  |  |  |  |
| 1 | Vvi-Vitvi05g04156\_t001 |  | \| \| |  |  |  |  |  |  |  |  |  |  |
| 1 | Vvi-Vitvi05g04157\_t001 |  | \| \| |  |  |  |  |  |  |  |  |  |  |
| 1 | Vvi-Vitvi05g00659\_t001 |  | \| \| |  |  |  |  |  |  |  |  |  |  |
| 1 | Vvi-Vitvi05g01908\_t001 |  | \| \| |  |  |  |  |  |  |  |  |  |  |
| 1 | Vvi-Vitvi05g04158\_t001 |  | \| \| |  |  |  |  |  |  |  |  |  |  |
| 1 | Vvi-Vitvi05g00661\_t001 |  | Atr-ERN01384 |  |  |  |  |  |  |  |  |  |  |
| 1 | Vvi-Vitvi05g00663\_t001 |  | \| \| |  |  |  |  |  |  |  |  |  |  |
| 1 | Vvi-Vitvi05g04159\_t001 |  | \| \| |  |  |  |  |  |  |  |  |  |  |
| 1 | Vvi-Vitvi05g00664\_t001 |  | \| \| |  |  |  |  |  |  |  |  |  |  |
| 1 | Vvi-Vitvi05g00666\_t001 |  | \| \| |  |  |  |  |  |  |  |  |  |  |
| 1 | Vvi-Vitvi05g04160\_t001 |  | Atr-ERN01371 |  |  |  |  |  |  |  |  |  |  |
| 0 | Vvi-Vitvi05g01909\_t001 |  |  |  |  |  |  |  |  |  |  |  |  |
| 0 | Vvi-Vitvi05g04161\_t001 |  |  |  |  |  |  |  |  |  |  |  |  |
| 0 | Vvi-Vitvi05g04162\_t001 |  |  |  |  |  |  |  |  |  |  |  |  |
| 0 | Vvi-Vitvi05g04163\_t001 |  |  |  |  |  |  |  |  |  |  |  |  |
| 0 | Vvi-Vitvi05g01911\_t001 |  |  |  |  |  |  |  |  |  |  |  |  |
| 0 | Vvi-Vitvi05g00669\_t001 |  |  |  |  |  |  |  |  |  |  |  |  |
| 0 | Vvi-Vitvi05g00670\_t001 |  |  |  |  |  |  |  |  |  |  |  |  |
| 0 | Vvi-Vitvi05g04164\_t001 |  |  |  |  |  |  |  |  |  |  |  |  |
| 0 | Vvi-Vitvi05g00675\_t001 |  |  |  |  |  |  |  |  |  |  |  |  |
| 0 | Vvi-Vitvi05g00676\_t001 |  |  |  |  |  |  |  |  |  |  |  |  |
| 0 | Vvi-Vitvi05g00679\_t001 |  |  |  |  |  |  |  |  |  |  |  |  |
| 0 | Vvi-Vitvi05g00680\_t001 |  |  |  |  |  |  |  |  |  |  |  |  |
| 0 | Vvi-Vitvi05g00681\_t001 |  |  |  |  |  |  |  |  |  |  |  |  |
| 0 | Vvi-Vitvi05g01912\_t001 |  |  |  |  |  |  |  |  |  |  |  |  |
| 1 | Vvi-Vitvi05g01913\_t001 |  | Atr-ERM94937 |  |  |  |  |  |  |  |  |  |  |
| 1 | Vvi-Vitvi05g01914\_t001 |  | \| \| |  |  |  |  |  |  |  |  |  |  |
| 1 | Vvi-Vitvi05g04165\_t001 |  | \| \| |  |  |  |  |  |  |  |  |  |  |
| 1 | Vvi-Vitvi05g01915\_t001 |  | \| \| |  |  |  |  |  |  |  |  |  |  |
| 1 | Vvi-Vitvi05g00684\_t001 |  | Atr-ERM94935 |  |  |  |  |  |  |  |  |  |  |
| 1 | Vvi-Vitvi05g01916\_t001 |  | Atr-ERM94933 |  |  |  |  |  |  |  |  |  |  |
| 1 | Vvi-Vitvi05g04166\_t001 |  | \| \| |  |  |  |  |  |  |  |  |  |  |
| 1 | Vvi-Vitvi05g00685\_t001 |  | \| \| |  |  |  |  |  |  |  |  |  |  |
| 1 | Vvi-Vitvi05g00686\_t001 |  | Atr-ERM94932 |  |  |  |  |  |  |  |  |  |  |
| 1 | Vvi-Vitvi05g01917\_t001 |  | \| \| |  |  |  |  |  |  |  |  |  |  |
| 1 | Vvi-Vitvi05g01918\_t001 |  | Atr-ERM94924 |  |  |  |  |  |  |  |  |  |  |
| 1 | Vvi-Vitvi05g00688\_t001 |  | Atr-ERM94923 |  |  |  |  |  |  |  |  |  |  |
| 1 | Vvi-Vitvi05g04167\_t001 |  | \| \| |  |  |  |  |  |  |  |  |  |  |
| 1 | Vvi-Vitvi05g04168\_t001 |  | \| \| |  |  |  |  |  |  |  |  |  |  |
| 1 | Vvi-Vitvi05g00690\_t001 |  | \| \| |  |  |  |  |  |  |  |  |  |  |
| 1 | Vvi-Vitvi05g04169\_t001 |  | \| \| |  |  |  |  |  |  |  |  |  |  |
| 1 | Vvi-Vitvi05g01920\_t001 |  | \| \| |  |  |  |  |  |  |  |  |  |  |
| 1 | Vvi-Vitvi05g00691\_t001 |  | Atr-ERM94920 |  |  |  |  |  |  |  |  |  |  |
| 1 | Vvi-Vitvi05g00692\_t001 |  | \| \| |  |  |  |  |  |  |  |  |  |  |
| 1 | Vvi-Vitvi05g04170\_t001 |  | \| \| |  |  |  |  |  |  |  |  |  |  |
| 1 | Vvi-Vitvi05g00694\_t001 |  | \| \| |  |  |  |  |  |  |  |  |  |  |
| 1 | Vvi-Vitvi05g04171\_t001 |  | \| \| |  |  |  |  |  |  |  |  |  |  |
| 1 | Vvi-Vitvi05g04172\_t001 |  | \| \| |  |  |  |  |  |  |  |  |  |  |
| 1 | Vvi-Vitvi05g04173\_t001 |  | \| \| |  |  |  |  |  |  |  |  |  |  |
| 1 | Vvi-Vitvi05g04174\_t001 |  | \| \| |  |  |  |  |  |  |  |  |  |  |
| 1 | Vvi-Vitvi05g01922\_t001 |  | \| \| |  |  |  |  |  |  |  |  |  |  |
| 1 | Vvi-Vitvi05g00698\_t001 |  | \| \| |  |  |  |  |  |  |  |  |  |  |
| 1 | Vvi-Vitvi05g01923\_t001 |  | \| \| |  |  |  |  |  |  |  |  |  |  |
| 1 | Vvi-Vitvi05g01924\_t001 |  | \| \| |  |  |  |  |  |  |  |  |  |  |
| 1 | Vvi-Vitvi05g00699\_t001 |  | \| \| |  |  |  |  |  |  |  |  |  |  |
| 1 | Vvi-Vitvi05g04175\_t001 |  | \| \| |  |  |  |  |  |  |  |  |  |  |
| 1 | Vvi-Vitvi05g01927\_t001 |  | \| \| |  |  |  |  |  |  |  |  |  |  |
| 1 | Vvi-Vitvi05g00701\_t001 |  | \| \| |  |  |  |  |  |  |  |  |  |  |
| 1 | Vvi-Vitvi05g00703\_t001 |  | \| \| |  |  |  |  |  |  |  |  |  |  |
| 1 | Vvi-Vitvi05g00705\_t001 |  | \| \| |  |  |  |  |  |  |  |  |  |  |
| 1 | Vvi-Vitvi05g01928\_t001 |  | \| \| |  |  |  |  |  |  |  |  |  |  |
| 1 | Vvi-Vitvi05g01930\_t004 |  | \| \| |  |  |  |  |  |  |  |  |  |  |
| 1 | Vvi-Vitvi05g00706\_t001 |  | \| \| |  |  |  |  |  |  |  |  |  |  |
| 1 | Vvi-Vitvi05g00707\_t001 |  | \| \| |  |  |  |  |  |  |  |  |  |  |
| 1 | Vvi-Vitvi05g00709\_t001 |  | \| \| |  |  |  |  |  |  |  |  |  |  |
| 1 | Vvi-Vitvi05g00710\_t001 |  | Atr-ERM94917 |  |  |  |  |  |  |  |  |  |  |
| 1 | Vvi-Vitvi05g00711\_t001 |  | \| \| |  |  |  |  |  |  |  |  |  |  |
| 1 | Vvi-Vitvi05g00712\_t001 |  | \| \| |  |  |  |  |  |  |  |  |  |  |
| 1 | Vvi-Vitvi05g00713\_t001 |  | Atr-ERM94915 |  |  |  |  |  |  |  |  |  |  |
| 1 | Vvi-Vitvi05g04176\_t001 |  | \| \| |  |  |  |  |  |  |  |  |  |  |
| 1 | Vvi-Vitvi05g01722\_t001 |  | Atr-ERM94898 |  |  |  |  |  |  |  |  |  |  |
| 1 | Vvi-Vitvi05g01723\_t001 |  | \| \| |  |  |  |  |  |  |  |  |  |  |
| 1 | Vvi-Vitvi05g00715\_t001 |  | Atr-ERM94896 |  |  |  |  |  |  |  |  |  |  |
| 0 | Vvi-Vitvi05g01931\_t001 |  |  |  |  |  |  |  |  |  |  |  |  |
| 0 | Vvi-Vitvi05g01933\_t001 |  |  |  |  |  |  |  |  |  |  |  |  |
| 0 | Vvi-Vitvi05g00718\_t001 |  |  |  |  |  |  |  |  |  |  |  |  |
| 0 | Vvi-Vitvi05g04177\_t001 |  |  |  |  |  |  |  |  |  |  |  |  |
| 0 | Vvi-Vitvi05g01939\_t001 |  |  |  |  |  |  |  |  |  |  |  |  |
| 0 | Vvi-Vitvi05g04178\_t001 |  |  |  |  |  |  |  |  |  |  |  |  |
| 0 | Vvi-Vitvi05g04179\_t001 |  |  |  |  |  |  |  |  |  |  |  |  |
| 0 | Vvi-Vitvi05g04180\_t001 |  |  |  |  |  |  |  |  |  |  |  |  |
| 0 | Vvi-Vitvi05g04181\_t001 |  |  |  |  |  |  |  |  |  |  |  |  |
| 0 | Vvi-Vitvi05g01940\_t001 |  |  |  |  |  |  |  |  |  |  |  |  |
| 0 | Vvi-Vitvi05g01941\_t001 |  |  |  |  |  |  |  |  |  |  |  |  |
| 0 | Vvi-Vitvi05g01942\_t001 |  |  |  |  |  |  |  |  |  |  |  |  |
| 0 | Vvi-Vitvi05g04182\_t001 |  |  |  |  |  |  |  |  |  |  |  |  |
| 0 | Vvi-Vitvi05g01943\_t001 |  |  |  |  |  |  |  |  |  |  |  |  |
| 0 | Vvi-Vitvi05g01944\_t001 |  |  |  |  |  |  |  |  |  |  |  |  |
| 0 | Vvi-Vitvi05g01945\_t001 |  |  |  |  |  |  |  |  |  |  |  |  |
| 0 | Vvi-Vitvi05g00726\_t001 |  |  |  |  |  |  |  |  |  |  |  |  |
| 0 | Vvi-Vitvi05g01946\_t001 |  |  |  |  |  |  |  |  |  |  |  |  |
| 0 | Vvi-Vitvi05g01947\_t001 |  |  |  |  |  |  |  |  |  |  |  |  |
| 0 | Vvi-Vitvi05g01948\_t001 |  |  |  |  |  |  |  |  |  |  |  |  |
| 0 | Vvi-Vitvi05g01949\_t001 |  |  |  |  |  |  |  |  |  |  |  |  |
| 0 | Vvi-Vitvi05g01950\_t001 |  |  |  |  |  |  |  |  |  |  |  |  |
| 0 | Vvi-Vitvi05g01951\_t001 |  |  |  |  |  |  |  |  |  |  |  |  |
| 0 | Vvi-Vitvi05g04183\_t001 |  |  |  |  |  |  |  |  |  |  |  |  |
| 0 | Vvi-Vitvi05g01952\_t001 |  |  |  |  |  |  |  |  |  |  |  |  |
| 0 | Vvi-Vitvi05g01953\_t001 |  |  |  |  |  |  |  |  |  |  |  |  |
| 0 | Vvi-Vitvi05g04184\_t001 |  |  |  |  |  |  |  |  |  |  |  |  |
| 0 | Vvi-Vitvi05g00731\_t002 |  |  |  |  |  |  |  |  |  |  |  |  |
| 0 | Vvi-Vitvi05g00733\_t001 |  |  |  |  |  |  |  |  |  |  |  |  |
| 0 | Vvi-Vitvi05g00734\_t001 |  |  |  |  |  |  |  |  |  |  |  |  |
| 0 | Vvi-Vitvi05g04185\_t001 |  |  |  |  |  |  |  |  |  |  |  |  |
| 0 | Vvi-Vitvi05g01956\_t001 |  |  |  |  |  |  |  |  |  |  |  |  |
| 0 | Vvi-Vitvi05g00736\_t001 |  |  |  |  |  |  |  |  |  |  |  |  |
| 0 | Vvi-Vitvi05g00737\_t002 |  |  |  |  |  |  |  |  |  |  |  |  |
| 0 | Vvi-Vitvi05g00739\_t001 |  |  |  |  |  |  |  |  |  |  |  |  |
| 0 | Vvi-Vitvi05g00740\_t001 |  |  |  |  |  |  |  |  |  |  |  |  |
| 0 | Vvi-Vitvi05g00742\_t001 |  |  |  |  |  |  |  |  |  |  |  |  |
| 0 | Vvi-Vitvi05g01732\_t001 |  |  |  |  |  |  |  |  |  |  |  |  |
| 0 | Vvi-Vitvi05g01733\_t001 |  |  |  |  |  |  |  |  |  |  |  |  |
| 1 | Vvi-Vitvi05g00746\_t001 |  | Atr-ERN18743 |  |  |  |  |  |  |  |  |  |  |
| 1 | Vvi-Vitvi05g01958\_t001 |  | \| \| |  |  |  |  |  |  |  |  |  |  |
| 1 | Vvi-Vitvi05g04186\_t001 |  | \| \| |  |  |  |  |  |  |  |  |  |  |
| 1 | Vvi-Vitvi05g00752\_t002 |  | Atr-ERN18755 |  |  |  |  |  |  |  |  |  |  |
| 1 | Vvi-Vitvi05g00753\_t001 |  | \| \| |  |  |  |  |  |  |  |  |  |  |
| 1 | Vvi-Vitvi05g04187\_t001 |  | \| \| |  |  |  |  |  |  |  |  |  |  |
| 1 | Vvi-Vitvi05g00755\_t002 |  | Atr-ERN18763 |  |  |  |  |  |  |  |  |  |  |
| 1 | Vvi-Vitvi05g00757\_t001 |  | Atr-ERN18766 |  |  |  |  |  |  |  |  |  |  |
| 1 | Vvi-Vitvi05g04188\_t001 |  | \| \| |  |  |  |  |  |  |  |  |  |  |
| 1 | Vvi-Vitvi05g04189\_t001 |  | \| \| |  |  |  |  |  |  |  |  |  |  |
| 1 | Vvi-Vitvi05g00759\_t001 |  | Atr-ERN18783 |  |  |  |  |  |  |  |  |  |  |
| 1 | Vvi-Vitvi05g00760\_t001 |  | \| \| |  |  |  |  |  |  |  |  |  |  |
| 1 | Vvi-Vitvi05g00761\_t001 |  | \| \| |  |  |  |  |  |  |  |  |  |  |
| 1 | Vvi-Vitvi05g00762\_t001 |  | \| \| |  |  |  |  |  |  |  |  |  |  |
| 1 | Vvi-Vitvi05g04190\_t001 |  | \| \| |  |  |  |  |  |  |  |  |  |  |
| 1 | Vvi-Vitvi05g00764\_t001 |  | Atr-ERN18795 |  |  |  |  |  |  |  |  |  |  |
| 1 | Vvi-Vitvi05g00765\_t001 |  | \| \| |  |  |  |  |  |  |  |  |  |  |
| 1 | Vvi-Vitvi05g00767\_t001 |  | Atr-ERN18798 |  |  |  |  |  |  |  |  |  |  |
| 1 | Vvi-Vitvi05g04191\_t001 |  | \| \| |  |  |  |  |  |  |  |  |  |  |
| 1 | Vvi-Vitvi05g04192\_t001 |  | \| \| |  |  |  |  |  |  |  |  |  |  |
| 1 | Vvi-Vitvi05g04193\_t001 |  | \| \| |  |  |  |  |  |  |  |  |  |  |
| 1 | Vvi-Vitvi05g04194\_t001 |  | \| \| |  |  |  |  |  |  |  |  |  |  |
| 1 | Vvi-Vitvi05g04195\_t001 |  | \| \| |  |  |  |  |  |  |  |  |  |  |
| 1 | Vvi-Vitvi05g00769\_t001 |  | Atr-ERN18800 |  |  |  |  |  |  |  |  |  |  |
| 1 | Vvi-Vitvi05g00770\_t001 |  | \| \| |  |  |  |  |  |  |  |  |  |  |
| 1 | Vvi-Vitvi05g00771\_t001 |  | Atr-ERN18801 |  |  |  |  |  |  |  |  |  |  |
| 1 | Vvi-Vitvi05g00772\_t001 |  | Atr-ERN18802 |  |  |  |  |  |  |  |  |  |  |
| 1 | Vvi-Vitvi05g00774\_t001 |  | Atr-ERN18808 |  |  |  |  |  |  |  |  |  |  |
| 1 | Vvi-Vitvi05g00775\_t001 |  | Atr-ERN18809 |  |  |  |  |  |  |  |  |  |  |
| 1 | Vvi-Vitvi05g00778\_t001 |  | Atr-ERN18811 |  |  |  |  |  |  |  |  |  |  |
| 1 | Vvi-Vitvi05g00779\_t001 |  | Atr-ERN18814 |  |  |  |  |  |  |  |  |  |  |
| 1 | Vvi-Vitvi05g01965\_t001 |  | Atr-ERN18815 |  |  |  |  |  |  |  |  |  |  |
| 1 | Vvi-Vitvi05g00780\_t001 |  | Atr-ERN18822 |  |  |  |  |  |  |  |  |  |  |
| 1 | Vvi-Vitvi05g00781\_t001 |  | \| \| |  |  |  |  |  |  |  |  |  |  |
| 1 | Vvi-Vitvi05g00782\_t001 |  | \| \| |  |  |  |  |  |  |  |  |  |  |
| 1 | Vvi-Vitvi05g00783\_t001 |  | Atr-ERN18823 |  |  |  |  |  |  |  |  |  |  |
| 1 | Vvi-Vitvi05g00784\_t001 |  | Atr-ERN18825 |  |  |  |  |  |  |  |  |  |  |
| 1 | Vvi-Vitvi05g00785\_t001 |  | Atr-ERN18829 |  |  |  |  |  |  |  |  |  |  |
| 0 | Vvi-Vitvi05g04196\_t001 |  |  |  |  |  |  |  |  |  |  |  |  |
| 1 | Vvi-Vitvi05g00787\_t001 |  | Atr-ERN18903 |  |  |  |  |  |  |  |  |  |  |
| 1 | Vvi-Vitvi05g00788\_t001 |  | \| \| |  |  |  |  |  |  |  |  |  |  |
| 1 | Vvi-Vitvi05g04197\_t001 |  | \| \| |  |  |  |  |  |  |  |  |  |  |
| 1 | Vvi-Vitvi05g00790\_t002 |  | Atr-ERN18911 |  |  |  |  |  |  |  |  |  |  |
| 1 | Vvi-Vitvi05g01725\_t001 |  | \| \| |  |  |  |  |  |  |  |  |  |  |
| 1 | Vvi-Vitvi05g00794\_t002 |  | \| \| |  |  |  |  |  |  |  |  |  |  |
| 1 | Vvi-Vitvi05g00795\_t001 |  | Atr-ERN18913 |  |  |  |  |  |  |  |  |  |  |
| 1 | Vvi-Vitvi05g00796\_t001 |  | Atr-ERN18915 |  |  |  |  |  |  |  |  |  |  |
| 1 | Vvi-Vitvi05g04198\_t001 |  | \| \| |  |  |  |  |  |  |  |  |  |  |
| 1 | Vvi-Vitvi05g00799\_t001 |  | Atr-ERN18921 |  |  |  |  |  |  |  |  |  |  |
| 1 | Vvi-Vitvi05g00800\_t001 |  | Atr-ERN18922 |  |  |  |  |  |  |  |  |  |  |
| 1 | Vvi-Vitvi05g00802\_t001 |  | Atr-ERN18925 |  |  |  |  |  |  |  |  |  |  |
| 1 | Vvi-Vitvi05g00803\_t001 |  | \| \| |  |  |  |  |  |  |  |  |  |  |
| 1 | Vvi-Vitvi05g04199\_t001 |  | \| \| |  |  |  |  |  |  |  |  |  |  |
| 1 | Vvi-Vitvi05g04200\_t001 |  | \| \| |  |  |  |  |  |  |  |  |  |  |
| 1 | Vvi-Vitvi05g04201\_t001 |  | \| \| |  |  |  |  |  |  |  |  |  |  |
| 1 | Vvi-Vitvi05g01972\_t001 |  | \| \| |  |  |  |  |  |  |  |  |  |  |
| 1 | Vvi-Vitvi05g04202\_t001 |  | \| \| |  |  |  |  |  |  |  |  |  |  |
| 1 | Vvi-Vitvi05g04203\_t001 |  | \| \| |  |  |  |  |  |  |  |  |  |  |
| 1 | Vvi-Vitvi05g04204\_t001 |  | \| \| |  |  |  |  |  |  |  |  |  |  |
| 1 | Vvi-Vitvi05g04205\_t001 |  | Atr-ERN18933 |  |  |  |  |  |  |  |  |  |  |
| 1 | Vvi-Vitvi05g04206\_t001 |  | \| \| |  |  |  |  |  |  |  |  |  |  |
| 1 | Vvi-Vitvi05g04207\_t001 |  | \| \| |  |  |  |  |  |  |  |  |  |  |
| 1 | Vvi-Vitvi05g00810\_t001 |  | \| \| |  |  |  |  |  |  |  |  |  |  |
| 1 | Vvi-Vitvi05g00811\_t001 |  | \| \| |  |  |  |  |  |  |  |  |  |  |
| 1 | Vvi-Vitvi05g00812\_t002 |  | \| \| |  |  |  |  |  |  |  |  |  |  |
| 1 | Vvi-Vitvi05g04208\_t001 |  | \| \| |  |  |  |  |  |  |  |  |  |  |
| 1 | Vvi-Vitvi05g00814\_t001 |  | \| \| |  |  |  |  |  |  |  |  |  |  |
| 1 | Vvi-Vitvi05g00815\_t001 |  | Atr-ERN18938 |  |  |  |  |  |  |  |  |  |  |
| 1 | Vvi-Vitvi05g04209\_t001 |  | \| \| |  |  |  |  |  |  |  |  |  |  |
| 1 | Vvi-Vitvi05g01981\_t001 |  | \| \| |  |  |  |  |  |  |  |  |  |  |
| 1 | Vvi-Vitvi05g00816\_t001 |  | Atr-ERN18953 |  |  |  |  |  |  |  |  |  |  |
| 1 | Vvi-Vitvi05g00817\_t001 |  | \| \| |  |  |  |  |  |  |  |  |  |  |
| 1 | Vvi-Vitvi05g00818\_t001 |  | Atr-ERN18956 |  |  |  |  |  |  |  |  |  |  |
| 1 | Vvi-Vitvi05g00819\_t001 |  | \| \| |  |  |  |  |  |  |  |  |  |  |
| 1 | Vvi-Vitvi05g04210\_t001 |  | Atr-ERN18964 |  |  |  |  |  |  |  |  |  |  |
| 2 | Vvi-Vitvi05g00821\_t001 |  | Atr-ERN18966 |  | Atr-ERN15944 |  |  |  |  |  |  |  |  |
| 1 | Vvi-Vitvi05g00822\_t001 |  |  |  | Atr-ERN15943 |  |  |  |  |  |  |  |  |
| 1 | Vvi-Vitvi05g04211\_t001 |  |  |  | \| \| |  |  |  |  |  |  |  |  |
| 1 | Vvi-Vitvi05g00823\_t001 |  |  |  | Atr-ERN15941 |  |  |  |  |  |  |  |  |
| 1 | Vvi-Vitvi05g04212\_t001 |  |  |  | \| \| |  |  |  |  |  |  |  |  |
| 1 | Vvi-Vitvi05g00825\_t001 |  |  |  | \| \| |  |  |  |  |  |  |  |  |
| 1 | Vvi-Vitvi05g00826\_t001 |  |  |  | \| \| |  |  |  |  |  |  |  |  |
| 1 | Vvi-Vitvi05g00828\_t001 |  |  |  | \| \| |  |  |  |  |  |  |  |  |
| 1 | Vvi-Vitvi05g00829\_t001 |  |  |  | \| \| |  |  |  |  |  |  |  |  |
| 1 | Vvi-Vitvi05g00830\_t001 |  |  |  | Atr-ERN15936 |  |  |  |  |  |  |  |  |
| 1 | Vvi-Vitvi05g00831\_t001 |  |  |  | \| \| |  |  |  |  |  |  |  |  |
| 1 | Vvi-Vitvi05g00832\_t001 |  |  |  | \| \| |  |  |  |  |  |  |  |  |
| 1 | Vvi-Vitvi05g00834\_t001 |  |  |  | \| \| |  |  |  |  |  |  |  |  |
| 1 | Vvi-Vitvi05g00835\_t002 |  |  |  | \| \| |  |  |  |  |  |  |  |  |
| 1 | Vvi-Vitvi05g04213\_t001 |  |  |  | \| \| |  |  |  |  |  |  |  |  |
| 1 | Vvi-Vitvi05g00836\_t002 |  |  |  | Atr-ERN15930 |  |  |  |  |  |  |  |  |
| 1 | Vvi-Vitvi05g04214\_t001 |  |  |  | \| \| |  |  |  |  |  |  |  |  |
| 1 | Vvi-Vitvi05g00837\_t001 |  |  |  | \| \| |  |  |  |  |  |  |  |  |
| 1 | Vvi-Vitvi05g00838\_t001 |  |  |  | \| \| |  |  |  |  |  |  |  |  |
| 1 | Vvi-Vitvi05g00839\_t001 |  |  |  | \| \| |  |  |  |  |  |  |  |  |
| 1 | Vvi-Vitvi05g04215\_t001 |  |  |  | \| \| |  |  |  |  |  |  |  |  |
| 1 | Vvi-Vitvi05g00841\_t001 |  |  |  | \| \| |  |  |  |  |  |  |  |  |
| 1 | Vvi-Vitvi05g04216\_t001 |  |  |  | \| \| |  |  |  |  |  |  |  |  |
| 1 | Vvi-Vitvi05g04217\_t001 |  |  |  | \| \| |  |  |  |  |  |  |  |  |
| 1 | Vvi-Vitvi05g00842\_t001 |  |  |  | \| \| |  |  |  |  |  |  |  |  |
| 1 | Vvi-Vitvi05g00845\_t001 |  |  |  | \| \| |  |  |  |  |  |  |  |  |
| 1 | Vvi-Vitvi05g04218\_t001 |  |  |  | \| \| |  |  |  |  |  |  |  |  |
| 1 | Vvi-Vitvi05g00847\_t001 |  |  |  | \| \| |  |  |  |  |  |  |  |  |
| 1 | Vvi-Vitvi05g00848\_t001 |  |  |  | \| \| |  |  |  |  |  |  |  |  |
| 1 | Vvi-Vitvi05g00849\_t001 |  |  |  | \| \| |  |  |  |  |  |  |  |  |
| 1 | Vvi-Vitvi05g01986\_t001 |  |  |  | \| \| |  |  |  |  |  |  |  |  |
| 1 | Vvi-Vitvi05g01987\_t001 |  |  |  | \| \| |  |  |  |  |  |  |  |  |
| 1 | Vvi-Vitvi05g01988\_t001 |  |  |  | \| \| |  |  |  |  |  |  |  |  |
| 1 | Vvi-Vitvi05g01989\_t001 |  |  |  | \| \| |  |  |  |  |  |  |  |  |
| 1 | Vvi-Vitvi05g00851\_t001 |  |  |  | \| \| |  |  |  |  |  |  |  |  |
| 1 | Vvi-Vitvi05g00852\_t001 |  |  |  | \| \| |  |  |  |  |  |  |  |  |
| 1 | Vvi-Vitvi05g01990\_t001 |  |  |  | \| \| |  |  |  |  |  |  |  |  |
| 1 | Vvi-Vitvi05g01991\_t001 |  |  |  | \| \| |  |  |  |  |  |  |  |  |
| 1 | Vvi-Vitvi05g04219\_t001 |  |  |  | \| \| |  |  |  |  |  |  |  |  |
| 1 | Vvi-Vitvi05g04220\_t001 |  |  |  | Atr-ERN15916 |  |  |  |  |  |  |  |  |
| 1 | Vvi-Vitvi05g01993\_t001 |  |  |  | \| \| |  |  |  |  |  |  |  |  |
| 1 | Vvi-Vitvi05g00855\_t001 |  |  |  | \| \| |  |  |  |  |  |  |  |  |
| 1 | Vvi-Vitvi05g00857\_t001 |  |  |  | \| \| |  |  |  |  |  |  |  |  |
| 1 | Vvi-Vitvi05g00858\_t001 |  |  |  | \| \| |  |  |  |  |  |  |  |  |
| 1 | Vvi-Vitvi05g00861\_t001 |  |  |  | \| \| |  |  |  |  |  |  |  |  |
| 1 | Vvi-Vitvi05g04221\_t001 |  |  |  | \| \| |  |  |  |  |  |  |  |  |
| 1 | Vvi-Vitvi05g04222\_t001 |  |  |  | \| \| |  |  |  |  |  |  |  |  |
| 1 | Vvi-Vitvi05g00862\_t001 |  |  |  | \| \| |  |  |  |  |  |  |  |  |
| 1 | Vvi-Vitvi05g00863\_t001 |  |  |  | \| \| |  |  |  |  |  |  |  |  |
| 1 | Vvi-Vitvi05g00864\_t001 |  |  |  | Atr-ERN15893 |  |  |  |  |  |  |  |  |
| 0 | Vvi-Vitvi05g04223\_t001 |  |  |  |  |  |  |  |  |  |  |  |  |
| 0 | Vvi-Vitvi05g00867\_t001 |  |  |  |  |  |  |  |  |  |  |  |  |
| 0 | Vvi-Vitvi05g00871\_t001 |  |  |  |  |  |  |  |  |  |  |  |  |
| 0 | Vvi-Vitvi05g00872\_t001 |  |  |  |  |  |  |  |  |  |  |  |  |
| 0 | Vvi-Vitvi05g00873\_t001 |  |  |  |  |  |  |  |  |  |  |  |  |
| 0 | Vvi-Vitvi05g00874\_t001 |  |  |  |  |  |  |  |  |  |  |  |  |
| 0 | Vvi-Vitvi05g00875\_t001 |  |  |  |  |  |  |  |  |  |  |  |  |
| 0 | Vvi-Vitvi05g01994\_t001 |  |  |  |  |  |  |  |  |  |  |  |  |
| 0 | Vvi-Vitvi05g00878\_t002 |  |  |  |  |  |  |  |  |  |  |  |  |
| 0 | Vvi-Vitvi05g04224\_t001 |  |  |  |  |  |  |  |  |  |  |  |  |
| 0 | Vvi-Vitvi05g04225\_t001 |  |  |  |  |  |  |  |  |  |  |  |  |
| 0 | Vvi-Vitvi05g04226\_t001 |  |  |  |  |  |  |  |  |  |  |  |  |
| 0 | Vvi-Vitvi05g00881\_t001 |  |  |  |  |  |  |  |  |  |  |  |  |
| 0 | Vvi-Vitvi05g00882\_t001 |  |  |  |  |  |  |  |  |  |  |  |  |
| 0 | Vvi-Vitvi05g00883\_t001 |  |  |  |  |  |  |  |  |  |  |  |  |
| 0 | Vvi-Vitvi05g01996\_t001 |  |  |  |  |  |  |  |  |  |  |  |  |
| 0 | Vvi-Vitvi05g00884\_t001 |  |  |  |  |  |  |  |  |  |  |  |  |
| 0 | Vvi-Vitvi05g00885\_t001 |  |  |  |  |  |  |  |  |  |  |  |  |
| 0 | Vvi-Vitvi05g04227\_t001 |  |  |  |  |  |  |  |  |  |  |  |  |
| 0 | Vvi-Vitvi05g00886\_t001 |  |  |  |  |  |  |  |  |  |  |  |  |
| 0 | Vvi-Vitvi05g04228\_t001 |  |  |  |  |  |  |  |  |  |  |  |  |
| 0 | Vvi-Vitvi05g04229\_t001 |  |  |  |  |  |  |  |  |  |  |  |  |
| 0 | Vvi-Vitvi05g00888\_t001 |  |  |  |  |  |  |  |  |  |  |  |  |
| 0 | Vvi-Vitvi05g01998\_t001 |  |  |  |  |  |  |  |  |  |  |  |  |
| 0 | Vvi-Vitvi05g00891\_t001 |  |  |  |  |  |  |  |  |  |  |  |  |
| 0 | Vvi-Vitvi05g01999\_t001 |  |  |  |  |  |  |  |  |  |  |  |  |
| 0 | Vvi-Vitvi05g04230\_t001 |  |  |  |  |  |  |  |  |  |  |  |  |
| 0 | Vvi-Vitvi05g04231\_t001 |  |  |  |  |  |  |  |  |  |  |  |  |
| 0 | Vvi-Vitvi05g00902\_t001 |  |  |  |  |  |  |  |  |  |  |  |  |
| 0 | Vvi-Vitvi05g00903\_t001 |  |  |  |  |  |  |  |  |  |  |  |  |
| 0 | Vvi-Vitvi05g00905\_t001 |  |  |  |  |  |  |  |  |  |  |  |  |
| 0 | Vvi-Vitvi05g04232\_t001 |  |  |  |  |  |  |  |  |  |  |  |  |
| 0 | Vvi-Vitvi05g02001\_t001 |  |  |  |  |  |  |  |  |  |  |  |  |
| 0 | Vvi-Vitvi05g04233\_t001 |  |  |  |  |  |  |  |  |  |  |  |  |
| 0 | Vvi-Vitvi05g00913\_t001 |  |  |  |  |  |  |  |  |  |  |  |  |
| 0 | Vvi-Vitvi05g04234\_t001 |  |  |  |  |  |  |  |  |  |  |  |  |
| 0 | Vvi-Vitvi05g00910\_t001 |  |  |  |  |  |  |  |  |  |  |  |  |
| 0 | Vvi-Vitvi05g04235\_t001 |  |  |  |  |  |  |  |  |  |  |  |  |
| 0 | Vvi-Vitvi05g04236\_t001 |  |  |  |  |  |  |  |  |  |  |  |  |
| 0 | Vvi-Vitvi05g04237\_t001 |  |  |  |  |  |  |  |  |  |  |  |  |
| 0 | Vvi-Vitvi05g02006\_t001 |  |  |  |  |  |  |  |  |  |  |  |  |
| 0 | Vvi-Vitvi05g04238\_t001 |  |  |  |  |  |  |  |  |  |  |  |  |
| 0 | Vvi-Vitvi05g00918\_t001 |  |  |  |  |  |  |  |  |  |  |  |  |
| 0 | Vvi-Vitvi05g00920\_t001 |  |  |  |  |  |  |  |  |  |  |  |  |
| 0 | Vvi-Vitvi05g00923\_t001 |  |  |  |  |  |  |  |  |  |  |  |  |
| 0 | Vvi-Vitvi05g00924\_t001 |  |  |  |  |  |  |  |  |  |  |  |  |
| 0 | Vvi-Vitvi05g00925\_t001 |  |  |  |  |  |  |  |  |  |  |  |  |
| 0 | Vvi-Vitvi05g00927\_t001 |  |  |  |  |  |  |  |  |  |  |  |  |
| 0 | Vvi-Vitvi05g00928\_t001 |  |  |  |  |  |  |  |  |  |  |  |  |
| 0 | Vvi-Vitvi05g00929\_t001 |  |  |  |  |  |  |  |  |  |  |  |  |
| 0 | Vvi-Vitvi05g02009\_t001 |  |  |  |  |  |  |  |  |  |  |  |  |
| 0 | Vvi-Vitvi05g00930\_t001 |  |  |  |  |  |  |  |  |  |  |  |  |
| 0 | Vvi-Vitvi05g00932\_t001 |  |  |  |  |  |  |  |  |  |  |  |  |
| 0 | Vvi-Vitvi05g00934\_t001 |  |  |  |  |  |  |  |  |  |  |  |  |
| 0 | Vvi-Vitvi05g04239\_t001 |  |  |  |  |  |  |  |  |  |  |  |  |
| 0 | Vvi-Vitvi05g00936\_t001 |  |  |  |  |  |  |  |  |  |  |  |  |
| 0 | Vvi-Vitvi05g00938\_t002 |  |  |  |  |  |  |  |  |  |  |  |  |
| 0 | Vvi-Vitvi05g04240\_t001 |  |  |  |  |  |  |  |  |  |  |  |  |
| 0 | Vvi-Vitvi05g00939\_t001 |  |  |  |  |  |  |  |  |  |  |  |  |
| 0 | Vvi-Vitvi05g00941\_t001 |  |  |  |  |  |  |  |  |  |  |  |  |
| 0 | Vvi-Vitvi05g00943\_t001 |  |  |  |  |  |  |  |  |  |  |  |  |
| 0 | Vvi-Vitvi05g00944\_t001 |  |  |  |  |  |  |  |  |  |  |  |  |
| 0 | Vvi-Vitvi05g04241\_t001 |  |  |  |  |  |  |  |  |  |  |  |  |
| 0 | Vvi-Vitvi05g02012\_t001 |  |  |  |  |  |  |  |  |  |  |  |  |
| 0 | Vvi-Vitvi05g02013\_t001 |  |  |  |  |  |  |  |  |  |  |  |  |
| 0 | Vvi-Vitvi05g00948\_t001 |  |  |  |  |  |  |  |  |  |  |  |  |
| 0 | Vvi-Vitvi05g00952\_t001 |  |  |  |  |  |  |  |  |  |  |  |  |
| 0 | Vvi-Vitvi05g00953\_t001 |  |  |  |  |  |  |  |  |  |  |  |  |
| 0 | Vvi-Vitvi05g04242\_t001 |  |  |  |  |  |  |  |  |  |  |  |  |
| 0 | Vvi-Vitvi05g00959\_t001 |  |  |  |  |  |  |  |  |  |  |  |  |
| 0 | Vvi-Vitvi05g00960\_t001 |  |  |  |  |  |  |  |  |  |  |  |  |
| 0 | Vvi-Vitvi05g04243\_t001 |  |  |  |  |  |  |  |  |  |  |  |  |
| 0 | Vvi-Vitvi05g00961\_t001 |  |  |  |  |  |  |  |  |  |  |  |  |
| 0 | Vvi-Vitvi05g00963\_t001 |  |  |  |  |  |  |  |  |  |  |  |  |
| 0 | Vvi-Vitvi05g02017\_t001 |  |  |  |  |  |  |  |  |  |  |  |  |
| 0 | Vvi-Vitvi05g02018\_t001 |  |  |  |  |  |  |  |  |  |  |  |  |
| 0 | Vvi-Vitvi05g02019\_t001 |  |  |  |  |  |  |  |  |  |  |  |  |
| 0 | Vvi-Vitvi05g04244\_t001 |  |  |  |  |  |  |  |  |  |  |  |  |
| 0 | Vvi-Vitvi05g00966\_t001 |  |  |  |  |  |  |  |  |  |  |  |  |
| 0 | Vvi-Vitvi05g02021\_t002 |  |  |  |  |  |  |  |  |  |  |  |  |
| 0 | Vvi-Vitvi05g04245\_t001 |  |  |  |  |  |  |  |  |  |  |  |  |
| 0 | Vvi-Vitvi05g04246\_t001 |  |  |  |  |  |  |  |  |  |  |  |  |
| 0 | Vvi-Vitvi05g04247\_t002 |  |  |  |  |  |  |  |  |  |  |  |  |
| 0 | Vvi-Vitvi05g04248\_t001 |  |  |  |  |  |  |  |  |  |  |  |  |
| 0 | Vvi-Vitvi05g04249\_t001 |  |  |  |  |  |  |  |  |  |  |  |  |
| 0 | Vvi-Vitvi05g04250\_t001 |  |  |  |  |  |  |  |  |  |  |  |  |
| 0 | Vvi-Vitvi05g04251\_t001 |  |  |  |  |  |  |  |  |  |  |  |  |
| 0 | Vvi-Vitvi05g04252\_t001 |  |  |  |  |  |  |  |  |  |  |  |  |
| 0 | Vvi-Vitvi05g00972\_t001 |  |  |  |  |  |  |  |  |  |  |  |  |
| 0 | Vvi-Vitvi05g04253\_t001 |  |  |  |  |  |  |  |  |  |  |  |  |
| 0 | Vvi-Vitvi05g00973\_t001 |  |  |  |  |  |  |  |  |  |  |  |  |
| 0 | Vvi-Vitvi05g00975\_t001 |  |  |  |  |  |  |  |  |  |  |  |  |
| 0 | Vvi-Vitvi05g00976\_t001 |  |  |  |  |  |  |  |  |  |  |  |  |
| 0 | Vvi-Vitvi05g02025\_t001 |  |  |  |  |  |  |  |  |  |  |  |  |
| 0 | Vvi-Vitvi05g00977\_t001 |  |  |  |  |  |  |  |  |  |  |  |  |
| 0 | Vvi-Vitvi05g00979\_t001 |  |  |  |  |  |  |  |  |  |  |  |  |
| 0 | Vvi-Vitvi05g00986\_t001 |  |  |  |  |  |  |  |  |  |  |  |  |
| 0 | Vvi-Vitvi05g02028\_t001 |  |  |  |  |  |  |  |  |  |  |  |  |
| 0 | Vvi-Vitvi05g00990\_t001 |  |  |  |  |  |  |  |  |  |  |  |  |
| 0 | Vvi-Vitvi05g00991\_t001 |  |  |  |  |  |  |  |  |  |  |  |  |
| 0 | Vvi-Vitvi05g04254\_t001 |  |  |  |  |  |  |  |  |  |  |  |  |
| 0 | Vvi-Vitvi05g00994\_t001 |  |  |  |  |  |  |  |  |  |  |  |  |
| 0 | Vvi-Vitvi05g04255\_t001 |  |  |  |  |  |  |  |  |  |  |  |  |
| 0 | Vvi-Vitvi05g04256\_t001 |  |  |  |  |  |  |  |  |  |  |  |  |
| 0 | Vvi-Vitvi05g04257\_t001 |  |  |  |  |  |  |  |  |  |  |  |  |
| 0 | Vvi-Vitvi05g04258\_t001 |  |  |  |  |  |  |  |  |  |  |  |  |
| 0 | Vvi-Vitvi05g04259\_t001 |  |  |  |  |  |  |  |  |  |  |  |  |
| 0 | Vvi-Vitvi05g04260\_t001 |  |  |  |  |  |  |  |  |  |  |  |  |
| 0 | Vvi-Vitvi05g04261\_t001 |  |  |  |  |  |  |  |  |  |  |  |  |
| 0 | Vvi-Vitvi05g01005\_t001 |  |  |  |  |  |  |  |  |  |  |  |  |
| 0 | Vvi-Vitvi05g04262\_t001 |  |  |  |  |  |  |  |  |  |  |  |  |
| 0 | Vvi-Vitvi05g04263\_t001 |  |  |  |  |  |  |  |  |  |  |  |  |
| 0 | Vvi-Vitvi05g04264\_t001 |  |  |  |  |  |  |  |  |  |  |  |  |
| 0 | Vvi-Vitvi05g04265\_t001 |  |  |  |  |  |  |  |  |  |  |  |  |
| 0 | Vvi-Vitvi05g01018\_t001 |  |  |  |  |  |  |  |  |  |  |  |  |
| 0 | Vvi-Vitvi05g01031\_t001 |  |  |  |  |  |  |  |  |  |  |  |  |
| 0 | Vvi-Vitvi05g01032\_t001 |  |  |  |  |  |  |  |  |  |  |  |  |
| 0 | Vvi-Vitvi05g04266\_t001 |  |  |  |  |  |  |  |  |  |  |  |  |
| 0 | Vvi-Vitvi05g04267\_t001 |  |  |  |  |  |  |  |  |  |  |  |  |
| 0 | Vvi-Vitvi05g01036\_t001 |  |  |  |  |  |  |  |  |  |  |  |  |
| 0 | Vvi-Vitvi05g04268\_t001 |  |  |  |  |  |  |  |  |  |  |  |  |
| 0 | Vvi-Vitvi05g01038\_t001 |  |  |  |  |  |  |  |  |  |  |  |  |
| 0 | Vvi-Vitvi05g04269\_t001 |  |  |  |  |  |  |  |  |  |  |  |  |
| 0 | Vvi-Vitvi05g02038\_t001 |  |  |  |  |  |  |  |  |  |  |  |  |
| 0 | Vvi-Vitvi05g01041\_t001 |  |  |  |  |  |  |  |  |  |  |  |  |
| 0 | Vvi-Vitvi05g01042\_t001 |  |  |  |  |  |  |  |  |  |  |  |  |
| 0 | Vvi-Vitvi05g04270\_t001 |  |  |  |  |  |  |  |  |  |  |  |  |
| 0 | Vvi-Vitvi05g01044\_t001 |  |  |  |  |  |  |  |  |  |  |  |  |
| 0 | Vvi-Vitvi05g01046\_t001 |  |  |  |  |  |  |  |  |  |  |  |  |
| 0 | Vvi-Vitvi05g01047\_t001 |  |  |  |  |  |  |  |  |  |  |  |  |
| 0 | Vvi-Vitvi05g01048\_t001 |  |  |  |  |  |  |  |  |  |  |  |  |
| 0 | Vvi-Vitvi05g04271\_t001 |  |  |  |  |  |  |  |  |  |  |  |  |
| 0 | Vvi-Vitvi05g04272\_t001 |  |  |  |  |  |  |  |  |  |  |  |  |
| 0 | Vvi-Vitvi05g04273\_t001 |  |  |  |  |  |  |  |  |  |  |  |  |
| 0 | Vvi-Vitvi05g04274\_t001 |  |  |  |  |  |  |  |  |  |  |  |  |
| 0 | Vvi-Vitvi05g01054\_t002 |  |  |  |  |  |  |  |  |  |  |  |  |
| 0 | Vvi-Vitvi05g01055\_t001 |  |  |  |  |  |  |  |  |  |  |  |  |
| 1 | Vvi-Vitvi05g01057\_t001 |  | Atr-ERN01814 |  |  |  |  |  |  |  |  |  |  |
| 1 | Vvi-Vitvi05g01059\_t001 |  | \| \| |  |  |  |  |  |  |  |  |  |  |
| 1 | Vvi-Vitvi05g01061\_t001 |  | \| \| |  |  |  |  |  |  |  |  |  |  |
| 1 | Vvi-Vitvi05g01063\_t001 |  | Atr-ERN01815 |  |  |  |  |  |  |  |  |  |  |
| 1 | Vvi-Vitvi05g04275\_t001 |  | \| \| |  |  |  |  |  |  |  |  |  |  |
| 1 | Vvi-Vitvi05g04276\_t001 |  | \| \| |  |  |  |  |  |  |  |  |  |  |
| 1 | Vvi-Vitvi05g01066\_t002 |  | Atr-ERN01820 |  |  |  |  |  |  |  |  |  |  |
| 1 | Vvi-Vitvi05g01068\_t001 |  | Atr-ERN01821 |  |  |  |  |  |  |  |  |  |  |
| 1 | Vvi-Vitvi05g01069\_t001 |  | Atr-ERN01825 |  |  |  |  |  |  |  |  |  |  |
| 1 | Vvi-Vitvi05g04277\_t001 |  | \| \| |  |  |  |  |  |  |  |  |  |  |
| 1 | Vvi-Vitvi05g04278\_t001 |  | \| \| |  |  |  |  |  |  |  |  |  |  |
| 1 | Vvi-Vitvi05g04279\_t001 |  | \| \| |  |  |  |  |  |  |  |  |  |  |
| 1 | Vvi-Vitvi05g02044\_t001 |  | Atr-ERN01827 |  |  |  |  |  |  |  |  |  |  |
| 0 | Vvi-Vitvi05g01072\_t001 |  |  |  |  |  |  |  |  |  |  |  |  |
| 0 | Vvi-Vitvi05g01073\_t001 |  |  |  |  |  |  |  |  |  |  |  |  |
| 0 | Vvi-Vitvi05g04280\_t001 |  |  |  |  |  |  |  |  |  |  |  |  |
| 0 | Vvi-Vitvi05g01075\_t001 |  |  |  |  |  |  |  |  |  |  |  |  |
| 0 | Vvi-Vitvi05g01076\_t001 |  |  |  |  |  |  |  |  |  |  |  |  |
| 0 | Vvi-Vitvi05g04281\_t001 |  |  |  |  |  |  |  |  |  |  |  |  |
| 0 | Vvi-Vitvi05g04282\_t001 |  |  |  |  |  |  |  |  |  |  |  |  |
| 0 | Vvi-Vitvi05g01080\_t001 |  |  |  |  |  |  |  |  |  |  |  |  |
| 0 | Vvi-Vitvi05g01081\_t001 |  |  |  |  |  |  |  |  |  |  |  |  |
| 0 | Vvi-Vitvi05g01082\_t001 |  |  |  |  |  |  |  |  |  |  |  |  |
| 0 | Vvi-Vitvi05g01087\_t001 |  |  |  |  |  |  |  |  |  |  |  |  |
| 0 | Vvi-Vitvi05g01088\_t001 |  |  |  |  |  |  |  |  |  |  |  |  |
| 0 | Vvi-Vitvi05g04283\_t001 |  |  |  |  |  |  |  |  |  |  |  |  |
| 1 | Vvi-Vitvi05g01091\_t001 |  | Atr-ERN10101 |  |  |  |  |  |  |  |  |  |  |
| 1 | Vvi-Vitvi05g01094\_t001 |  | Atr-ERN10099 |  |  |  |  |  |  |  |  |  |  |
| 1 | Vvi-Vitvi05g01095\_t001 |  | \| \| |  |  |  |  |  |  |  |  |  |  |
| 1 | Vvi-Vitvi05g01096\_t001 |  | \| \| |  |  |  |  |  |  |  |  |  |  |
| 1 | Vvi-Vitvi05g01097\_t001 |  | \| \| |  |  |  |  |  |  |  |  |  |  |
| 1 | Vvi-Vitvi05g02048\_t001 |  | \| \| |  |  |  |  |  |  |  |  |  |  |
| 1 | Vvi-Vitvi05g04284\_t001 |  | \| \| |  |  |  |  |  |  |  |  |  |  |
| 1 | Vvi-Vitvi05g04285\_t001 |  | \| \| |  |  |  |  |  |  |  |  |  |  |
| 1 | Vvi-Vitvi05g02049\_t001 |  | \| \| |  |  |  |  |  |  |  |  |  |  |
| 1 | Vvi-Vitvi05g01103\_t001 |  | \| \| |  |  |  |  |  |  |  |  |  |  |
| 1 | Vvi-Vitvi05g01104\_t001 |  | \| \| |  |  |  |  |  |  |  |  |  |  |
| 1 | Vvi-Vitvi05g01106\_t001 |  | \| \| |  |  |  |  |  |  |  |  |  |  |
| 1 | Vvi-Vitvi05g01108\_t001 |  | Atr-ERN10075 |  |  |  |  |  |  |  |  |  |  |
| 1 | Vvi-Vitvi05g01112\_t001 |  | Atr-ERN10073 |  |  |  |  |  |  |  |  |  |  |
| 1 | Vvi-Vitvi05g01113\_t001 |  | \| \| |  |  |  |  |  |  |  |  |  |  |
| 1 | Vvi-Vitvi05g01114\_t001 |  | \| \| |  |  |  |  |  |  |  |  |  |  |
| 1 | Vvi-Vitvi05g04286\_t001 |  | \| \| |  |  |  |  |  |  |  |  |  |  |
| 1 | Vvi-Vitvi05g01115\_t001 |  | Atr-ERN10071 |  |  |  |  |  |  |  |  |  |  |
| 1 | Vvi-Vitvi05g01116\_t001 |  | \| \| |  |  |  |  |  |  |  |  |  |  |
| 1 | Vvi-Vitvi05g04287\_t001 |  | \| \| |  |  |  |  |  |  |  |  |  |  |
| 1 | Vvi-Vitvi05g04288\_t001 |  | \| \| |  |  |  |  |  |  |  |  |  |  |
| 1 | Vvi-Vitvi05g01117\_t001 |  | \| \| |  |  |  |  |  |  |  |  |  |  |
| 1 | Vvi-Vitvi05g01118\_t001 |  | \| \| |  |  |  |  |  |  |  |  |  |  |
| 1 | Vvi-Vitvi05g04289\_t001 |  | \| \| |  |  |  |  |  |  |  |  |  |  |
| 1 | Vvi-Vitvi05g02051\_t001 |  | \| \| |  |  |  |  |  |  |  |  |  |  |
| 1 | Vvi-Vitvi05g02052\_t001 |  | \| \| |  |  |  |  |  |  |  |  |  |  |
| 1 | Vvi-Vitvi05g04290\_t001 |  | \| \| |  |  |  |  |  |  |  |  |  |  |
| 1 | Vvi-Vitvi05g04291\_t001 |  | \| \| |  |  |  |  |  |  |  |  |  |  |
| 1 | Vvi-Vitvi05g01120\_t001 |  | \| \| |  |  |  |  |  |  |  |  |  |  |
| 1 | Vvi-Vitvi05g01121\_t001 |  | \| \| |  |  |  |  |  |  |  |  |  |  |
| 1 | Vvi-Vitvi05g04292\_t001 |  | \| \| |  |  |  |  |  |  |  |  |  |  |
| 1 | Vvi-Vitvi05g04293\_t001 |  | \| \| |  |  |  |  |  |  |  |  |  |  |
| 1 | Vvi-Vitvi05g01124\_t001 |  | Atr-ERN10062 |  |  |  |  |  |  |  |  |  |  |
| 1 | Vvi-Vitvi05g01125\_t001 |  | \| \| |  |  |  |  |  |  |  |  |  |  |
| 1 | Vvi-Vitvi05g01126\_t001 |  | \| \| |  |  |  |  |  |  |  |  |  |  |
| 1 | Vvi-Vitvi05g01128\_t001 |  | Atr-ERN10060 |  |  |  |  |  |  |  |  |  |  |
| 1 | Vvi-Vitvi05g01129\_t001 |  | Atr-ERN10053 |  |  |  |  |  |  |  |  |  |  |
| 0 | Vvi-Vitvi05g02056\_t001 |  |  |  |  |  |  |  |  |  |  |  |  |
| 0 | Vvi-Vitvi05g01130\_t002 |  |  |  |  |  |  |  |  |  |  |  |  |
| 0 | Vvi-Vitvi05g01135\_t001 |  |  |  |  |  |  |  |  |  |  |  |  |
| 0 | Vvi-Vitvi05g04294\_t001 |  |  |  |  |  |  |  |  |  |  |  |  |
| 0 | Vvi-Vitvi05g01136\_t001 |  |  |  |  |  |  |  |  |  |  |  |  |
| 0 | Vvi-Vitvi05g04295\_t001 |  |  |  |  |  |  |  |  |  |  |  |  |
| 0 | Vvi-Vitvi05g01137\_t001 |  |  |  |  |  |  |  |  |  |  |  |  |
| 0 | Vvi-Vitvi05g01138\_t001 |  |  |  |  |  |  |  |  |  |  |  |  |
| 0 | Vvi-Vitvi05g04296\_t001 |  |  |  |  |  |  |  |  |  |  |  |  |
| 0 | Vvi-Vitvi05g04297\_t001 |  |  |  |  |  |  |  |  |  |  |  |  |
| 0 | Vvi-Vitvi05g04298\_t001 |  |  |  |  |  |  |  |  |  |  |  |  |
| 0 | Vvi-Vitvi05g04299\_t001 |  |  |  |  |  |  |  |  |  |  |  |  |
| 0 | Vvi-Vitvi05g04300\_t001 |  |  |  |  |  |  |  |  |  |  |  |  |
| 0 | Vvi-Vitvi05g04301\_t001 |  |  |  |  |  |  |  |  |  |  |  |  |
| 0 | Vvi-Vitvi05g04302\_t001 |  |  |  |  |  |  |  |  |  |  |  |  |
| 0 | Vvi-Vitvi05g01145\_t001 |  |  |  |  |  |  |  |  |  |  |  |  |
| 0 | Vvi-Vitvi05g04303\_t001 |  |  |  |  |  |  |  |  |  |  |  |  |
| 0 | Vvi-Vitvi05g04304\_t001 |  |  |  |  |  |  |  |  |  |  |  |  |
| 0 | Vvi-Vitvi05g04305\_t001 |  |  |  |  |  |  |  |  |  |  |  |  |
| 0 | Vvi-Vitvi05g04306\_t001 |  |  |  |  |  |  |  |  |  |  |  |  |
| 0 | Vvi-Vitvi05g04307\_t001 |  |  |  |  |  |  |  |  |  |  |  |  |
| 0 | Vvi-Vitvi05g04308\_t001 |  |  |  |  |  |  |  |  |  |  |  |  |
| 0 | Vvi-Vitvi05g04309\_t001 |  |  |  |  |  |  |  |  |  |  |  |  |
| 0 | Vvi-Vitvi05g04310\_t001 |  |  |  |  |  |  |  |  |  |  |  |  |
| 0 | Vvi-Vitvi05g01153\_t001 |  |  |  |  |  |  |  |  |  |  |  |  |
| 0 | Vvi-Vitvi05g04311\_t001 |  |  |  |  |  |  |  |  |  |  |  |  |
| 0 | Vvi-Vitvi05g02059\_t001 |  |  |  |  |  |  |  |  |  |  |  |  |
| 0 | Vvi-Vitvi05g01154\_t001 |  |  |  |  |  |  |  |  |  |  |  |  |
| 0 | Vvi-Vitvi05g01159\_t001 |  |  |  |  |  |  |  |  |  |  |  |  |
| 0 | Vvi-Vitvi05g04312\_t001 |  |  |  |  |  |  |  |  |  |  |  |  |
| 0 | Vvi-Vitvi05g04313\_t001 |  |  |  |  |  |  |  |  |  |  |  |  |
| 0 | Vvi-Vitvi05g01164\_t001 |  |  |  |  |  |  |  |  |  |  |  |  |
| 0 | Vvi-Vitvi05g04314\_t001 |  |  |  |  |  |  |  |  |  |  |  |  |
| 0 | Vvi-Vitvi05g04315\_t001 |  |  |  |  |  |  |  |  |  |  |  |  |
| 0 | Vvi-Vitvi05g04316\_t001 |  |  |  |  |  |  |  |  |  |  |  |  |
| 0 | Vvi-Vitvi05g02074\_t001 |  |  |  |  |  |  |  |  |  |  |  |  |
| 0 | Vvi-Vitvi05g04317\_t001 |  |  |  |  |  |  |  |  |  |  |  |  |
| 0 | Vvi-Vitvi05g01178\_t001 |  |  |  |  |  |  |  |  |  |  |  |  |
| 0 | Vvi-Vitvi05g01179\_t001 |  |  |  |  |  |  |  |  |  |  |  |  |
| 0 | Vvi-Vitvi05g04318\_t001 |  |  |  |  |  |  |  |  |  |  |  |  |
| 0 | Vvi-Vitvi05g01181\_t001 |  |  |  |  |  |  |  |  |  |  |  |  |
| 0 | Vvi-Vitvi05g04319\_t001 |  |  |  |  |  |  |  |  |  |  |  |  |
| 0 | Vvi-Vitvi05g04320\_t001 |  |  |  |  |  |  |  |  |  |  |  |  |
| 0 | Vvi-Vitvi05g04321\_t001 |  |  |  |  |  |  |  |  |  |  |  |  |
| 0 | Vvi-Vitvi05g04322\_t001 |  |  |  |  |  |  |  |  |  |  |  |  |
| 0 | Vvi-Vitvi05g01184\_t001 |  |  |  |  |  |  |  |  |  |  |  |  |
| 0 | Vvi-Vitvi05g01185\_t004 |  |  |  |  |  |  |  |  |  |  |  |  |
| 0 | Vvi-Vitvi05g01186\_t001 |  |  |  |  |  |  |  |  |  |  |  |  |
| 0 | Vvi-Vitvi05g04323\_t001 |  |  |  |  |  |  |  |  |  |  |  |  |
| 0 | Vvi-Vitvi05g01189\_t001 |  |  |  |  |  |  |  |  |  |  |  |  |
| 0 | Vvi-Vitvi05g04324\_t001 |  |  |  |  |  |  |  |  |  |  |  |  |
| 0 | Vvi-Vitvi05g04325\_t001 |  |  |  |  |  |  |  |  |  |  |  |  |
| 0 | Vvi-Vitvi05g01192\_t001 |  |  |  |  |  |  |  |  |  |  |  |  |
| 0 | Vvi-Vitvi05g02077\_t001 |  |  |  |  |  |  |  |  |  |  |  |  |
| 0 | Vvi-Vitvi05g01193\_t003 |  |  |  |  |  |  |  |  |  |  |  |  |
| 0 | Vvi-Vitvi05g01195\_t001 |  |  |  |  |  |  |  |  |  |  |  |  |
| 0 | Vvi-Vitvi05g01197\_t001 |  |  |  |  |  |  |  |  |  |  |  |  |
| 0 | Vvi-Vitvi05g01198\_t001 |  |  |  |  |  |  |  |  |  |  |  |  |
| 0 | Vvi-Vitvi05g01200\_t001 |  |  |  |  |  |  |  |  |  |  |  |  |
| 0 | Vvi-Vitvi05g01201\_t001 |  |  |  |  |  |  |  |  |  |  |  |  |
| 0 | Vvi-Vitvi05g01202\_t001 |  |  |  |  |  |  |  |  |  |  |  |  |
| 0 | Vvi-Vitvi05g04326\_t001 |  |  |  |  |  |  |  |  |  |  |  |  |
| 0 | Vvi-Vitvi05g01209\_t001 |  |  |  |  |  |  |  |  |  |  |  |  |
| 0 | Vvi-Vitvi05g02078\_t001 |  |  |  |  |  |  |  |  |  |  |  |  |
| 0 | Vvi-Vitvi05g04327\_t001 |  |  |  |  |  |  |  |  |  |  |  |  |
| 0 | Vvi-Vitvi05g01213\_t001 |  |  |  |  |  |  |  |  |  |  |  |  |
| 0 | Vvi-Vitvi05g02079\_t001 |  |  |  |  |  |  |  |  |  |  |  |  |
| 0 | Vvi-Vitvi05g01214\_t001 |  |  |  |  |  |  |  |  |  |  |  |  |
| 0 | Vvi-Vitvi05g01216\_t001 |  |  |  |  |  |  |  |  |  |  |  |  |
| 0 | Vvi-Vitvi05g01219\_t001 |  |  |  |  |  |  |  |  |  |  |  |  |
| 0 | Vvi-Vitvi05g01221\_t002 |  |  |  |  |  |  |  |  |  |  |  |  |
| 0 | Vvi-Vitvi05g04328\_t001 |  |  |  |  |  |  |  |  |  |  |  |  |
| 0 | Vvi-Vitvi05g04329\_t001 |  |  |  |  |  |  |  |  |  |  |  |  |
| 0 | Vvi-Vitvi05g01223\_t001 |  |  |  |  |  |  |  |  |  |  |  |  |
| 0 | Vvi-Vitvi05g02082\_t001 |  |  |  |  |  |  |  |  |  |  |  |  |
| 0 | Vvi-Vitvi05g04330\_t001 |  |  |  |  |  |  |  |  |  |  |  |  |
| 0 | Vvi-Vitvi05g04331\_t001 |  |  |  |  |  |  |  |  |  |  |  |  |
| 0 | Vvi-Vitvi05g04332\_t001 |  |  |  |  |  |  |  |  |  |  |  |  |
| 0 | Vvi-Vitvi05g04333\_t001 |  |  |  |  |  |  |  |  |  |  |  |  |
| 0 | Vvi-Vitvi05g02085\_t004 |  |  |  |  |  |  |  |  |  |  |  |  |
| 0 | Vvi-Vitvi05g04334\_t001 |  |  |  |  |  |  |  |  |  |  |  |  |
| 0 | Vvi-Vitvi05g04335\_t001 |  |  |  |  |  |  |  |  |  |  |  |  |
| 0 | Vvi-Vitvi05g01228\_t001 |  |  |  |  |  |  |  |  |  |  |  |  |
| 0 | Vvi-Vitvi05g02088\_t002 |  |  |  |  |  |  |  |  |  |  |  |  |
| 0 | Vvi-Vitvi05g02089\_t001 |  |  |  |  |  |  |  |  |  |  |  |  |
| 0 | Vvi-Vitvi05g01229\_t001 |  |  |  |  |  |  |  |  |  |  |  |  |
| 0 | Vvi-Vitvi05g04336\_t001 |  |  |  |  |  |  |  |  |  |  |  |  |
| 0 | Vvi-Vitvi05g04337\_t001 |  |  |  |  |  |  |  |  |  |  |  |  |
| 0 | Vvi-Vitvi05g02090\_t001 |  |  |  |  |  |  |  |  |  |  |  |  |
| 0 | Vvi-Vitvi05g01231\_t001 |  |  |  |  |  |  |  |  |  |  |  |  |
| 0 | Vvi-Vitvi05g04338\_t001 |  |  |  |  |  |  |  |  |  |  |  |  |
| 0 | Vvi-Vitvi05g04339\_t001 |  |  |  |  |  |  |  |  |  |  |  |  |
| 0 | Vvi-Vitvi05g04340\_t001 |  |  |  |  |  |  |  |  |  |  |  |  |
| 0 | Vvi-Vitvi05g04341\_t001 |  |  |  |  |  |  |  |  |  |  |  |  |
| 0 | Vvi-Vitvi05g04342\_t001 |  |  |  |  |  |  |  |  |  |  |  |  |
| 0 | Vvi-Vitvi05g04343\_t001 |  |  |  |  |  |  |  |  |  |  |  |  |
| 0 | Vvi-Vitvi05g04344\_t001 |  |  |  |  |  |  |  |  |  |  |  |  |
| 0 | Vvi-Vitvi05g04345\_t001 |  |  |  |  |  |  |  |  |  |  |  |  |
| 0 | Vvi-Vitvi05g04346\_t001 |  |  |  |  |  |  |  |  |  |  |  |  |
| 0 | Vvi-Vitvi05g04347\_t001 |  |  |  |  |  |  |  |  |  |  |  |  |
| 0 | Vvi-Vitvi05g04348\_t001 |  |  |  |  |  |  |  |  |  |  |  |  |
| 0 | Vvi-Vitvi05g02096\_t001 |  |  |  |  |  |  |  |  |  |  |  |  |
| 0 | Vvi-Vitvi05g04349\_t001 |  |  |  |  |  |  |  |  |  |  |  |  |
| 0 | Vvi-Vitvi05g04350\_t001 |  |  |  |  |  |  |  |  |  |  |  |  |
| 0 | Vvi-Vitvi05g04351\_t001 |  |  |  |  |  |  |  |  |  |  |  |  |
| 0 | Vvi-Vitvi05g01242\_t001 |  |  |  |  |  |  |  |  |  |  |  |  |
| 0 | Vvi-Vitvi05g02097\_t001 |  |  |  |  |  |  |  |  |  |  |  |  |
| 0 | Vvi-Vitvi05g02099\_t003 |  |  |  |  |  |  |  |  |  |  |  |  |
| 0 | Vvi-Vitvi05g02100\_t001 |  |  |  |  |  |  |  |  |  |  |  |  |
| 0 | Vvi-Vitvi05g04352\_t001 |  |  |  |  |  |  |  |  |  |  |  |  |
| 0 | Vvi-Vitvi05g01247\_t001 |  |  |  |  |  |  |  |  |  |  |  |  |
| 0 | Vvi-Vitvi05g01248\_t001 |  |  |  |  |  |  |  |  |  |  |  |  |
| 0 | Vvi-Vitvi05g01250\_t001 |  |  |  |  |  |  |  |  |  |  |  |  |
| 0 | Vvi-Vitvi05g01252\_t001 |  |  |  |  |  |  |  |  |  |  |  |  |
| 0 | Vvi-Vitvi05g01254\_t001 |  |  |  |  |  |  |  |  |  |  |  |  |
| 0 | Vvi-Vitvi05g01255\_t001 |  |  |  |  |  |  |  |  |  |  |  |  |
| 0 | Vvi-Vitvi05g01256\_t001 |  |  |  |  |  |  |  |  |  |  |  |  |
| 0 | Vvi-Vitvi05g01257\_t001 |  |  |  |  |  |  |  |  |  |  |  |  |
| 0 | Vvi-Vitvi05g01258\_t002 |  |  |  |  |  |  |  |  |  |  |  |  |
| 0 | Vvi-Vitvi05g04353\_t001 |  |  |  |  |  |  |  |  |  |  |  |  |
| 0 | Vvi-Vitvi05g04354\_t001 |  |  |  |  |  |  |  |  |  |  |  |  |
| 0 | Vvi-Vitvi05g01263\_t001 |  |  |  |  |  |  |  |  |  |  |  |  |
| 0 | Vvi-Vitvi05g01264\_t001 |  |  |  |  |  |  |  |  |  |  |  |  |
| 0 | Vvi-Vitvi05g01265\_t001 |  |  |  |  |  |  |  |  |  |  |  |  |
| 0 | Vvi-Vitvi05g01266\_t001 |  |  |  |  |  |  |  |  |  |  |  |  |
| 0 | Vvi-Vitvi05g01267\_t001 |  |  |  |  |  |  |  |  |  |  |  |  |
| 0 | Vvi-Vitvi05g01269\_t001 |  |  |  |  |  |  |  |  |  |  |  |  |
| 0 | Vvi-Vitvi05g04355\_t001 |  |  |  |  |  |  |  |  |  |  |  |  |
| 0 | Vvi-Vitvi05g04356\_t001 |  |  |  |  |  |  |  |  |  |  |  |  |
| 0 | Vvi-Vitvi05g04357\_t001 |  |  |  |  |  |  |  |  |  |  |  |  |
| 0 | Vvi-Vitvi05g04358\_t001 |  |  |  |  |  |  |  |  |  |  |  |  |
| 0 | Vvi-Vitvi05g02103\_t001 |  |  |  |  |  |  |  |  |  |  |  |  |
| 0 | Vvi-Vitvi05g04360\_t001 |  |  |  |  |  |  |  |  |  |  |  |  |
| 0 | Vvi-Vitvi05g02104\_t001 |  |  |  |  |  |  |  |  |  |  |  |  |
| 0 | Vvi-Vitvi05g01273\_t001 |  |  |  |  |  |  |  |  |  |  |  |  |
| 0 | Vvi-Vitvi05g01274\_t001 |  |  |  |  |  |  |  |  |  |  |  |  |
| 0 | Vvi-Vitvi05g01276\_t001 |  |  |  |  |  |  |  |  |  |  |  |  |
| 0 | Vvi-Vitvi05g01278\_t001 |  |  |  |  |  |  |  |  |  |  |  |  |
| 0 | Vvi-Vitvi05g01279\_t001 |  |  |  |  |  |  |  |  |  |  |  |  |
| 0 | Vvi-Vitvi05g02108\_t001 |  |  |  |  |  |  |  |  |  |  |  |  |
| 0 | Vvi-Vitvi05g01285\_t001 |  |  |  |  |  |  |  |  |  |  |  |  |
| 0 | Vvi-Vitvi05g04361\_t001 |  |  |  |  |  |  |  |  |  |  |  |  |
| 0 | Vvi-Vitvi05g04362\_t001 |  |  |  |  |  |  |  |  |  |  |  |  |
| 0 | Vvi-Vitvi05g01288\_t001 |  |  |  |  |  |  |  |  |  |  |  |  |
| 0 | Vvi-Vitvi05g01289\_t001 |  |  |  |  |  |  |  |  |  |  |  |  |
| 0 | Vvi-Vitvi05g02111\_t001 |  |  |  |  |  |  |  |  |  |  |  |  |
| 0 | Vvi-Vitvi05g02112\_t001 |  |  |  |  |  |  |  |  |  |  |  |  |
| 0 | Vvi-Vitvi05g01291\_t001 |  |  |  |  |  |  |  |  |  |  |  |  |
| 0 | Vvi-Vitvi05g04363\_t001 |  |  |  |  |  |  |  |  |  |  |  |  |
| 0 | Vvi-Vitvi05g01294\_t001 |  |  |  |  |  |  |  |  |  |  |  |  |
| 0 | Vvi-Vitvi05g04364\_t001 |  |  |  |  |  |  |  |  |  |  |  |  |
| 0 | Vvi-Vitvi05g04365\_t001 |  |  |  |  |  |  |  |  |  |  |  |  |
| 0 | Vvi-Vitvi05g01299\_t001 |  |  |  |  |  |  |  |  |  |  |  |  |
| 0 | Vvi-Vitvi05g01300\_t001 |  |  |  |  |  |  |  |  |  |  |  |  |
| 0 | Vvi-Vitvi05g01301\_t001 |  |  |  |  |  |  |  |  |  |  |  |  |
| 0 | Vvi-Vitvi05g01304\_t001 |  |  |  |  |  |  |  |  |  |  |  |  |
| 0 | Vvi-Vitvi05g02116\_t001 |  |  |  |  |  |  |  |  |  |  |  |  |
| 0 | Vvi-Vitvi05g01306\_t001 |  |  |  |  |  |  |  |  |  |  |  |  |
| 0 | Vvi-Vitvi05g01307\_t001 |  |  |  |  |  |  |  |  |  |  |  |  |
| 0 | Vvi-Vitvi05g01308\_t002 |  |  |  |  |  |  |  |  |  |  |  |  |
| 0 | Vvi-Vitvi05g01309\_t001 |  |  |  |  |  |  |  |  |  |  |  |  |
| 0 | Vvi-Vitvi05g01310\_t001 |  |  |  |  |  |  |  |  |  |  |  |  |
| 0 | Vvi-Vitvi05g01313\_t001 |  |  |  |  |  |  |  |  |  |  |  |  |
| 0 | Vvi-Vitvi05g01314\_t001 |  |  |  |  |  |  |  |  |  |  |  |  |
| 0 | Vvi-Vitvi05g01317\_t001 |  |  |  |  |  |  |  |  |  |  |  |  |
| 0 | Vvi-Vitvi05g01318\_t001 |  |  |  |  |  |  |  |  |  |  |  |  |
| 0 | Vvi-Vitvi05g01319\_t001 |  |  |  |  |  |  |  |  |  |  |  |  |
| 0 | Vvi-Vitvi05g01320\_t001 |  |  |  |  |  |  |  |  |  |  |  |  |
| 0 | Vvi-Vitvi05g04366\_t001 |  |  |  |  |  |  |  |  |  |  |  |  |
| 0 | Vvi-Vitvi05g01323\_t001 |  |  |  |  |  |  |  |  |  |  |  |  |
| 0 | Vvi-Vitvi05g01324\_t002 |  |  |  |  |  |  |  |  |  |  |  |  |
| 0 | Vvi-Vitvi05g01325\_t001 |  |  |  |  |  |  |  |  |  |  |  |  |
| 0 | Vvi-Vitvi05g04367\_t001 |  |  |  |  |  |  |  |  |  |  |  |  |
| 0 | Vvi-Vitvi05g01327\_t002 |  |  |  |  |  |  |  |  |  |  |  |  |
| 0 | Vvi-Vitvi05g01328\_t001 |  |  |  |  |  |  |  |  |  |  |  |  |
| 0 | Vvi-Vitvi05g01330\_t001 |  |  |  |  |  |  |  |  |  |  |  |  |
| 0 | Vvi-Vitvi05g04368\_t001 |  |  |  |  |  |  |  |  |  |  |  |  |
| 0 | Vvi-Vitvi05g01334\_t001 |  |  |  |  |  |  |  |  |  |  |  |  |
| 0 | Vvi-Vitvi05g04369\_t001 |  |  |  |  |  |  |  |  |  |  |  |  |
| 0 | Vvi-Vitvi05g01336\_t001 |  |  |  |  |  |  |  |  |  |  |  |  |
| 0 | Vvi-Vitvi05g01337\_t001 |  |  |  |  |  |  |  |  |  |  |  |  |
| 0 | Vvi-Vitvi05g04370\_t001 |  |  |  |  |  |  |  |  |  |  |  |  |
| 0 | Vvi-Vitvi05g01338\_t001 |  |  |  |  |  |  |  |  |  |  |  |  |
| 0 | Vvi-Vitvi05g01339\_t001 |  |  |  |  |  |  |  |  |  |  |  |  |
| 0 | Vvi-Vitvi05g01340\_t001 |  |  |  |  |  |  |  |  |  |  |  |  |
| 0 | Vvi-Vitvi05g01341\_t001 |  |  |  |  |  |  |  |  |  |  |  |  |
| 0 | Vvi-Vitvi05g01342\_t004 |  |  |  |  |  |  |  |  |  |  |  |  |
| 0 | Vvi-Vitvi05g01343\_t001 |  |  |  |  |  |  |  |  |  |  |  |  |
| 0 | Vvi-Vitvi05g01344\_t001 |  |  |  |  |  |  |  |  |  |  |  |  |
| 0 | Vvi-Vitvi05g02120\_t001 |  |  |  |  |  |  |  |  |  |  |  |  |
| 0 | Vvi-Vitvi05g01345\_t001 |  |  |  |  |  |  |  |  |  |  |  |  |
| 0 | Vvi-Vitvi05g04371\_t001 |  |  |  |  |  |  |  |  |  |  |  |  |
| 0 | Vvi-Vitvi05g01347\_t001 |  |  |  |  |  |  |  |  |  |  |  |  |
| 0 | Vvi-Vitvi05g01348\_t001 |  |  |  |  |  |  |  |  |  |  |  |  |
| 0 | Vvi-Vitvi05g04372\_t001 |  |  |  |  |  |  |  |  |  |  |  |  |
| 0 | Vvi-Vitvi05g01350\_t001 |  |  |  |  |  |  |  |  |  |  |  |  |
| 0 | Vvi-Vitvi05g01351\_t001 |  |  |  |  |  |  |  |  |  |  |  |  |
| 0 | Vvi-Vitvi05g01352\_t001 |  |  |  |  |  |  |  |  |  |  |  |  |
| 0 | Vvi-Vitvi05g02121\_t001 |  |  |  |  |  |  |  |  |  |  |  |  |
| 0 | Vvi-Vitvi05g01353\_t001 |  |  |  |  |  |  |  |  |  |  |  |  |
| 0 | Vvi-Vitvi05g01355\_t001 |  |  |  |  |  |  |  |  |  |  |  |  |
| 0 | Vvi-Vitvi05g01356\_t001 |  |  |  |  |  |  |  |  |  |  |  |  |
| 0 | Vvi-Vitvi05g01357\_t001 |  |  |  |  |  |  |  |  |  |  |  |  |
| 0 | Vvi-Vitvi05g04373\_t001 |  |  |  |  |  |  |  |  |  |  |  |  |
| 0 | Vvi-Vitvi05g01358\_t001 |  |  |  |  |  |  |  |  |  |  |  |  |
| 0 | Vvi-Vitvi05g04374\_t001 |  |  |  |  |  |  |  |  |  |  |  |  |
| 0 | Vvi-Vitvi05g01359\_t001 |  |  |  |  |  |  |  |  |  |  |  |  |
| 0 | Vvi-Vitvi05g04375\_t001 |  |  |  |  |  |  |  |  |  |  |  |  |
| 0 | Vvi-Vitvi05g01360\_t001 |  |  |  |  |  |  |  |  |  |  |  |  |
| 0 | Vvi-Vitvi05g01361\_t001 |  |  |  |  |  |  |  |  |  |  |  |  |
| 0 | Vvi-Vitvi05g02122\_t001 |  |  |  |  |  |  |  |  |  |  |  |  |
| 0 | Vvi-Vitvi05g02123\_t001 |  |  |  |  |  |  |  |  |  |  |  |  |
| 0 | Vvi-Vitvi05g01362\_t001 |  |  |  |  |  |  |  |  |  |  |  |  |
| 0 | Vvi-Vitvi05g04376\_t001 |  |  |  |  |  |  |  |  |  |  |  |  |
| 0 | Vvi-Vitvi05g01363\_t001 |  |  |  |  |  |  |  |  |  |  |  |  |
| 0 | Vvi-Vitvi05g01364\_t001 |  |  |  |  |  |  |  |  |  |  |  |  |
| 0 | Vvi-Vitvi05g01365\_t001 |  |  |  |  |  |  |  |  |  |  |  |  |
| 0 | Vvi-Vitvi05g01366\_t001 |  |  |  |  |  |  |  |  |  |  |  |  |
| 0 | Vvi-Vitvi05g01367\_t001 |  |  |  |  |  |  |  |  |  |  |  |  |
| 0 | Vvi-Vitvi05g02124\_t001 |  |  |  |  |  |  |  |  |  |  |  |  |
| 0 | Vvi-Vitvi05g01369\_t001 |  |  |  |  |  |  |  |  |  |  |  |  |
| 0 | Vvi-Vitvi05g01370\_t001 |  |  |  |  |  |  |  |  |  |  |  |  |
| 0 | Vvi-Vitvi05g02125\_t001 |  |  |  |  |  |  |  |  |  |  |  |  |
| 0 | Vvi-Vitvi05g04377\_t001 |  |  |  |  |  |  |  |  |  |  |  |  |
| 0 | Vvi-Vitvi05g02126\_t001 |  |  |  |  |  |  |  |  |  |  |  |  |
| 0 | Vvi-Vitvi05g02127\_t001 |  |  |  |  |  |  |  |  |  |  |  |  |
| 0 | Vvi-Vitvi05g04378\_t001 |  |  |  |  |  |  |  |  |  |  |  |  |
| 0 | Vvi-Vitvi05g04379\_t001 |  |  |  |  |  |  |  |  |  |  |  |  |
| 0 | Vvi-Vitvi05g01372\_t001 |  |  |  |  |  |  |  |  |  |  |  |  |
| 0 | Vvi-Vitvi05g04380\_t001 |  |  |  |  |  |  |  |  |  |  |  |  |
| 0 | Vvi-Vitvi05g02128\_t001 |  |  |  |  |  |  |  |  |  |  |  |  |
| 0 | Vvi-Vitvi05g01375\_t001 |  |  |  |  |  |  |  |  |  |  |  |  |
| 0 | Vvi-Vitvi05g01376\_t001 |  |  |  |  |  |  |  |  |  |  |  |  |
| 0 | Vvi-Vitvi05g04381\_t001 |  |  |  |  |  |  |  |  |  |  |  |  |
| 0 | Vvi-Vitvi05g04382\_t001 |  |  |  |  |  |  |  |  |  |  |  |  |
| 0 | Vvi-Vitvi05g01378\_t001 |  |  |  |  |  |  |  |  |  |  |  |  |
| 0 | Vvi-Vitvi05g01379\_t001 |  |  |  |  |  |  |  |  |  |  |  |  |
| 0 | Vvi-Vitvi05g04383\_t001 |  |  |  |  |  |  |  |  |  |  |  |  |
| 0 | Vvi-Vitvi05g01380\_t001 |  |  |  |  |  |  |  |  |  |  |  |  |
| 0 | Vvi-Vitvi05g01381\_t002 |  |  |  |  |  |  |  |  |  |  |  |  |
| 0 | Vvi-Vitvi05g01382\_t001 |  |  |  |  |  |  |  |  |  |  |  |  |
| 0 | Vvi-Vitvi05g04384\_t001 |  |  |  |  |  |  |  |  |  |  |  |  |
| 0 | Vvi-Vitvi05g02131\_t001 |  |  |  |  |  |  |  |  |  |  |  |  |
| 0 | Vvi-Vitvi05g01383\_t001 |  |  |  |  |  |  |  |  |  |  |  |  |
| 1 | Vvi-Vitvi05g04385\_t001 |  | Atr-ERN20093 |  |  |  |  |  |  |  |  |  |  |
| 1 | Vvi-Vitvi05g04386\_t001 |  | \| \| |  |  |  |  |  |  |  |  |  |  |
| 1 | Vvi-Vitvi05g01388\_t001 |  | \| \| |  |  |  |  |  |  |  |  |  |  |
| 1 | Vvi-Vitvi05g01389\_t001 |  | \| \| |  |  |  |  |  |  |  |  |  |  |
| 1 | Vvi-Vitvi05g01390\_t001 |  | \| \| |  |  |  |  |  |  |  |  |  |  |
| 1 | Vvi-Vitvi05g04387\_t001 |  | \| \| |  |  |  |  |  |  |  |  |  |  |
| 1 | Vvi-Vitvi05g04388\_t001 |  | \| \| |  |  |  |  |  |  |  |  |  |  |
| 1 | Vvi-Vitvi05g04389\_t001 |  | \| \| |  |  |  |  |  |  |  |  |  |  |
| 1 | Vvi-Vitvi05g01392\_t001 |  | \| \| |  |  |  |  |  |  |  |  |  |  |
| 1 | Vvi-Vitvi05g01393\_t002 |  | \| \| |  |  |  |  |  |  |  |  |  |  |
| 1 | Vvi-Vitvi05g02135\_t001 |  | \| \| |  |  |  |  |  |  |  |  |  |  |
| 1 | Vvi-Vitvi05g02136\_t001 |  | \| \| |  |  |  |  |  |  |  |  |  |  |
| 1 | Vvi-Vitvi05g01395\_t001 |  | Atr-ERN20099 |  |  |  |  |  |  |  |  |  |  |
| 1 | Vvi-Vitvi05g01396\_t001 |  | Atr-ERN20100 |  |  |  |  |  |  |  |  |  |  |
| 1 | Vvi-Vitvi05g04390\_t001 |  | \| \| |  |  |  |  |  |  |  |  |  |  |
| 1 | Vvi-Vitvi05g01399\_t001 |  | \| \| |  |  |  |  |  |  |  |  |  |  |
| 1 | Vvi-Vitvi05g01400\_t001 |  | \| \| |  |  |  |  |  |  |  |  |  |  |
| 2 | Vvi-Vitvi05g01401\_t001 |  | \| \| |  | Atr-ERN20110 |  |  |  |  |  |  |  |  |
| 2 | Vvi-Vitvi05g04391\_t001 |  | \| \| |  | \| \| |  |  |  |  |  |  |  |  |
| 2 | Vvi-Vitvi05g01404\_t001 |  | \| \| |  | \| \| |  |  |  |  |  |  |  |  |
| 2 | Vvi-Vitvi05g01405\_t001 |  | \| \| |  | Atr-ERN20109 |  |  |  |  |  |  |  |  |
| 2 | Vvi-Vitvi05g04392\_t001 |  | \| \| |  | \| \| |  |  |  |  |  |  |  |  |
| 2 | Vvi-Vitvi05g01408\_t001 |  | Atr-ERN20105 |  | Atr-ERN20105 |  |  |  |  |  |  |  |  |
| 2 | Vvi-Vitvi05g01409\_t003 |  | \| \| |  | Atr-ERN20104 |  |  |  |  |  |  |  |  |
| 2 | Vvi-Vitvi05g01410\_t001 |  | \| \| |  | \| \| |  |  |  |  |  |  |  |  |
| 2 | Vvi-Vitvi05g01411\_t001 |  | \| \| |  | \| \| |  |  |  |  |  |  |  |  |
| 2 | Vvi-Vitvi05g01412\_t001 |  | \| \| |  | \| \| |  |  |  |  |  |  |  |  |
| 2 | Vvi-Vitvi05g04393\_t001 |  | \| \| |  | \| \| |  |  |  |  |  |  |  |  |
| 2 | Vvi-Vitvi05g01413\_t001 |  | \| \| |  | Atr-ERN20102 |  |  |  |  |  |  |  |  |
| 2 | Vvi-Vitvi05g04394\_t001 |  | \| \| |  | \| \| |  |  |  |  |  |  |  |  |
| 2 | Vvi-Vitvi05g04395\_t001 |  | \| \| |  | \| \| |  |  |  |  |  |  |  |  |
| 2 | Vvi-Vitvi05g01417\_t001 |  | \| \| |  | \| \| |  |  |  |  |  |  |  |  |
| 2 | Vvi-Vitvi05g01419\_t001 |  | \| \| |  | \| \| |  |  |  |  |  |  |  |  |
| 2 | Vvi-Vitvi05g02138\_t004 |  | \| \| |  | Atr-ERN20101 |  |  |  |  |  |  |  |  |
| 1 | Vvi-Vitvi05g01420\_t001 |  | \| \| |  |  |  |  |  |  |  |  |  |  |
| 1 | Vvi-Vitvi05g01421\_t001 |  | Atr-ERN20111 |  |  |  |  |  |  |  |  |  |  |
| 1 | Vvi-Vitvi05g02139\_t001 |  | Atr-ERN20113 |  |  |  |  |  |  |  |  |  |  |
| 1 | Vvi-Vitvi05g04396\_t001 |  | \| \| |  |  |  |  |  |  |  |  |  |  |
| 1 | Vvi-Vitvi05g01424\_t001 |  | Atr-ERN20115 |  |  |  |  |  |  |  |  |  |  |
| 0 | Vvi-Vitvi05g02141\_t001 |  |  |  |  |  |  |  |  |  |  |  |  |
| 0 | Vvi-Vitvi05g04397\_t001 |  |  |  |  |  |  |  |  |  |  |  |  |
| 0 | Vvi-Vitvi05g04398\_t001 |  |  |  |  |  |  |  |  |  |  |  |  |
| 0 | Vvi-Vitvi05g04399\_t001 |  |  |  |  |  |  |  |  |  |  |  |  |
| 0 | Vvi-Vitvi05g04400\_t001 |  |  |  |  |  |  |  |  |  |  |  |  |
| 0 | Vvi-Vitvi05g04401\_t001 |  |  |  |  |  |  |  |  |  |  |  |  |
| 0 | Vvi-Vitvi05g04402\_t001 |  |  |  |  |  |  |  |  |  |  |  |  |
| 0 | Vvi-Vitvi05g04403\_t001 |  |  |  |  |  |  |  |  |  |  |  |  |
| 0 | Vvi-Vitvi05g02145\_t001 |  |  |  |  |  |  |  |  |  |  |  |  |
| 0 | Vvi-Vitvi05g02146\_t001 |  |  |  |  |  |  |  |  |  |  |  |  |
| 0 | Vvi-Vitvi05g04404\_t001 |  |  |  |  |  |  |  |  |  |  |  |  |
| 0 | Vvi-Vitvi05g02148\_t001 |  |  |  |  |  |  |  |  |  |  |  |  |
| 0 | Vvi-Vitvi05g01435\_t001 |  |  |  |  |  |  |  |  |  |  |  |  |
| 0 | Vvi-Vitvi05g04405\_t001 |  |  |  |  |  |  |  |  |  |  |  |  |
| 0 | Vvi-Vitvi05g04406\_t001 |  |  |  |  |  |  |  |  |  |  |  |  |
| 0 | Vvi-Vitvi05g04407\_t001 |  |  |  |  |  |  |  |  |  |  |  |  |
| 0 | Vvi-Vitvi05g04408\_t002 |  |  |  |  |  |  |  |  |  |  |  |  |
| 0 | Vvi-Vitvi05g04409\_t001 |  |  |  |  |  |  |  |  |  |  |  |  |
| 0 | Vvi-Vitvi05g04410\_t001 |  |  |  |  |  |  |  |  |  |  |  |  |
| 0 | Vvi-Vitvi05g04411\_t001 |  |  |  |  |  |  |  |  |  |  |  |  |
| 0 | Vvi-Vitvi05g04412\_t001 |  |  |  |  |  |  |  |  |  |  |  |  |
| 0 | Vvi-Vitvi05g04413\_t001 |  |  |  |  |  |  |  |  |  |  |  |  |
| 0 | Vvi-Vitvi05g04414\_t001 |  |  |  |  |  |  |  |  |  |  |  |  |
| 0 | Vvi-Vitvi05g01437\_t001 |  |  |  |  |  |  |  |  |  |  |  |  |
| 0 | Vvi-Vitvi05g04415\_t001 |  |  |  |  |  |  |  |  |  |  |  |  |
| 0 | Vvi-Vitvi05g04416\_t001 |  |  |  |  |  |  |  |  |  |  |  |  |
| 0 | Vvi-Vitvi05g04417\_t001 |  |  |  |  |  |  |  |  |  |  |  |  |
| 0 | Vvi-Vitvi05g04418\_t001 |  |  |  |  |  |  |  |  |  |  |  |  |
| 0 | Vvi-Vitvi05g04419\_t001 |  |  |  |  |  |  |  |  |  |  |  |  |
| 0 | Vvi-Vitvi05g04420\_t001 |  |  |  |  |  |  |  |  |  |  |  |  |
| 0 | Vvi-Vitvi05g04421\_t001 |  |  |  |  |  |  |  |  |  |  |  |  |
| 0 | Vvi-Vitvi05g02153\_t001 |  |  |  |  |  |  |  |  |  |  |  |  |
| 0 | Vvi-Vitvi05g04422\_t001 |  |  |  |  |  |  |  |  |  |  |  |  |
| 0 | Vvi-Vitvi05g02156\_t001 |  |  |  |  |  |  |  |  |  |  |  |  |
| 0 | Vvi-Vitvi05g02157\_t001 |  |  |  |  |  |  |  |  |  |  |  |  |
| 0 | Vvi-Vitvi05g04423\_t001 |  |  |  |  |  |  |  |  |  |  |  |  |
| 0 | Vvi-Vitvi05g04424\_t001 |  |  |  |  |  |  |  |  |  |  |  |  |
| 0 | Vvi-Vitvi05g04425\_t001 |  |  |  |  |  |  |  |  |  |  |  |  |
| 0 | Vvi-Vitvi05g04426\_t001 |  |  |  |  |  |  |  |  |  |  |  |  |
| 0 | Vvi-Vitvi05g04427\_t001 |  |  |  |  |  |  |  |  |  |  |  |  |
| 0 | Vvi-Vitvi05g04428\_t001 |  |  |  |  |  |  |  |  |  |  |  |  |
| 0 | Vvi-Vitvi05g04429\_t001 |  |  |  |  |  |  |  |  |  |  |  |  |
| 0 | Vvi-Vitvi05g01447\_t001 |  |  |  |  |  |  |  |  |  |  |  |  |
| 0 | Vvi-Vitvi05g04430\_t001 |  |  |  |  |  |  |  |  |  |  |  |  |
| 0 | Vvi-Vitvi05g04431\_t001 |  |  |  |  |  |  |  |  |  |  |  |  |
| 0 | Vvi-Vitvi05g04432\_t001 |  |  |  |  |  |  |  |  |  |  |  |  |
| 0 | Vvi-Vitvi05g01450\_t001 |  |  |  |  |  |  |  |  |  |  |  |  |
| 0 | Vvi-Vitvi05g04433\_t001 |  |  |  |  |  |  |  |  |  |  |  |  |
| 0 | Vvi-Vitvi05g01452\_t001 |  |  |  |  |  |  |  |  |  |  |  |  |
| 0 | Vvi-Vitvi05g01453\_t001 |  |  |  |  |  |  |  |  |  |  |  |  |
| 0 | Vvi-Vitvi05g01454\_t001 |  |  |  |  |  |  |  |  |  |  |  |  |
| 0 | Vvi-Vitvi05g04434\_t001 |  |  |  |  |  |  |  |  |  |  |  |  |
| 0 | Vvi-Vitvi05g04435\_t001 |  |  |  |  |  |  |  |  |  |  |  |  |
| 0 | Vvi-Vitvi05g01456\_t001 |  |  |  |  |  |  |  |  |  |  |  |  |
| 0 | Vvi-Vitvi05g04436\_t001 |  |  |  |  |  |  |  |  |  |  |  |  |
| 0 | Vvi-Vitvi05g02166\_t001 |  |  |  |  |  |  |  |  |  |  |  |  |
| 0 | Vvi-Vitvi05g01459\_t001 |  |  |  |  |  |  |  |  |  |  |  |  |
| 0 | Vvi-Vitvi05g02167\_t001 |  |  |  |  |  |  |  |  |  |  |  |  |
| 0 | Vvi-Vitvi05g02168\_t001 |  |  |  |  |  |  |  |  |  |  |  |  |
| 0 | Vvi-Vitvi05g02170\_t001 |  |  |  |  |  |  |  |  |  |  |  |  |
| 0 | Vvi-Vitvi05g01464\_t001 |  |  |  |  |  |  |  |  |  |  |  |  |
| 1 | Vvi-Vitvi05g01465\_t001 |  | Atr-ERN20118 |  |  |  |  |  |  |  |  |  |  |
| 1 | Vvi-Vitvi05g01466\_t001 |  | \| \| |  |  |  |  |  |  |  |  |  |  |
| 1 | Vvi-Vitvi05g01467\_t001 |  | Atr-ERN20119 |  |  |  |  |  |  |  |  |  |  |
| 1 | Vvi-Vitvi05g01468\_t002 |  | \| \| |  |  |  |  |  |  |  |  |  |  |
| 1 | Vvi-Vitvi05g01469\_t001 |  | Atr-ERN20122 |  |  |  |  |  |  |  |  |  |  |
| 1 | Vvi-Vitvi05g04437\_t001 |  | \| \| |  |  |  |  |  |  |  |  |  |  |
| 1 | Vvi-Vitvi05g01470\_t001 |  | Atr-ERN20126 |  |  |  |  |  |  |  |  |  |  |
| 1 | Vvi-Vitvi05g01471\_t001 |  | Atr-ERN20133 |  |  |  |  |  |  |  |  |  |  |
| 1 | Vvi-Vitvi05g04438\_t001 |  | \| \| |  |  |  |  |  |  |  |  |  |  |
| 1 | Vvi-Vitvi05g01475\_t001 |  | Atr-ERN20136 |  |  |  |  |  |  |  |  |  |  |
| 1 | Vvi-Vitvi05g01477\_t001 |  | \| \| |  |  |  |  |  |  |  |  |  |  |
| 1 | Vvi-Vitvi05g01478\_t001 |  | \| \| |  |  |  |  |  |  |  |  |  |  |
| 1 | Vvi-Vitvi05g01479\_t001 |  | \| \| |  |  |  |  |  |  |  |  |  |  |
| 1 | Vvi-Vitvi05g01480\_t001 |  | Atr-ERN20142 |  |  |  |  |  |  |  |  |  |  |
| 1 | Vvi-Vitvi05g01481\_t001 |  | \| \| |  |  |  |  |  |  |  |  |  |  |
| 1 | Vvi-Vitvi05g01482\_t001 |  | Atr-ERN20148 |  |  |  |  |  |  |  |  |  |  |
| 1 | Vvi-Vitvi05g01484\_t001 |  | \| \| |  |  |  |  |  |  |  |  |  |  |
| 1 | Vvi-Vitvi05g01485\_t001 |  | \| \| |  |  |  |  |  |  |  |  |  |  |
| 1 | Vvi-Vitvi05g01486\_t001 |  | Atr-ERN20150 |  |  |  |  |  |  |  |  |  |  |
| 1 | Vvi-Vitvi05g04439\_t001 |  | \| \| |  |  |  |  |  |  |  |  |  |  |
| 1 | Vvi-Vitvi05g01488\_t001 |  | Atr-ERN20151 |  |  |  |  |  |  |  |  |  |  |
| 1 | Vvi-Vitvi05g04440\_t001 |  | \| \| |  |  |  |  |  |  |  |  |  |  |
| 1 | Vvi-Vitvi05g01489\_t001 |  | \| \| |  |  |  |  |  |  |  |  |  |  |
| 1 | Vvi-Vitvi05g01490\_t001 |  | \| \| |  |  |  |  |  |  |  |  |  |  |
| 1 | Vvi-Vitvi05g01492\_t001 |  | \| \| |  |  |  |  |  |  |  |  |  |  |
| 1 | Vvi-Vitvi05g01493\_t001 |  | \| \| |  |  |  |  |  |  |  |  |  |  |
| 1 | Vvi-Vitvi05g04441\_t001 |  | \| \| |  |  |  |  |  |  |  |  |  |  |
| 1 | Vvi-Vitvi05g01494\_t001 |  | \| \| |  |  |  |  |  |  |  |  |  |  |
| 1 | Vvi-Vitvi05g01495\_t001 |  | \| \| |  |  |  |  |  |  |  |  |  |  |
| 1 | Vvi-Vitvi05g01497\_t001 |  | \| \| |  |  |  |  |  |  |  |  |  |  |
| 1 | Vvi-Vitvi05g01498\_t002 |  | \| \| |  |  |  |  |  |  |  |  |  |  |
| 1 | Vvi-Vitvi05g02178\_t001 |  | \| \| |  |  |  |  |  |  |  |  |  |  |
| 1 | Vvi-Vitvi05g01499\_t001 |  | \| \| |  |  |  |  |  |  |  |  |  |  |
| 1 | Vvi-Vitvi05g04442\_t001 |  | \| \| |  |  |  |  |  |  |  |  |  |  |
| 1 | Vvi-Vitvi05g01500\_t001 |  | \| \| |  |  |  |  |  |  |  |  |  |  |
| 1 | Vvi-Vitvi05g04443\_t001 |  | \| \| |  |  |  |  |  |  |  |  |  |  |
| 1 | Vvi-Vitvi05g01501\_t001 |  | \| \| |  |  |  |  |  |  |  |  |  |  |
| 1 | Vvi-Vitvi05g04444\_t001 |  | \| \| |  |  |  |  |  |  |  |  |  |  |
| 1 | Vvi-Vitvi05g04445\_t001 |  | Atr-ERN20175 |  |  |  |  |  |  |  |  |  |  |
| 0 | Vvi-Vitvi05g01502\_t001 |  |  |  |  |  |  |  |  |  |  |  |  |
| 0 | Vvi-Vitvi05g04446\_t001 |  |  |  |  |  |  |  |  |  |  |  |  |
| 0 | Vvi-Vitvi05g04447\_t001 |  |  |  |  |  |  |  |  |  |  |  |  |
| 0 | Vvi-Vitvi05g04448\_t001 |  |  |  |  |  |  |  |  |  |  |  |  |
| 0 | Vvi-Vitvi05g04449\_t001 |  |  |  |  |  |  |  |  |  |  |  |  |
| 0 | Vvi-Vitvi05g02180\_t001 |  |  |  |  |  |  |  |  |  |  |  |  |
| 0 | Vvi-Vitvi05g04450\_t001 |  |  |  |  |  |  |  |  |  |  |  |  |
| 0 | Vvi-Vitvi05g04451\_t001 |  |  |  |  |  |  |  |  |  |  |  |  |
| 0 | Vvi-Vitvi05g04452\_t001 |  |  |  |  |  |  |  |  |  |  |  |  |
| 0 | Vvi-Vitvi05g02182\_t001 |  |  |  |  |  |  |  |  |  |  |  |  |
| 0 | Vvi-Vitvi05g04453\_t001 |  |  |  |  |  |  |  |  |  |  |  |  |
| 0 | Vvi-Vitvi05g01506\_t001 |  |  |  |  |  |  |  |  |  |  |  |  |
| 0 | Vvi-Vitvi05g01507\_t001 |  |  |  |  |  |  |  |  |  |  |  |  |
| 0 | Vvi-Vitvi05g01508\_t001 |  |  |  |  |  |  |  |  |  |  |  |  |
| 0 | Vvi-Vitvi05g04454\_t001 |  |  |  |  |  |  |  |  |  |  |  |  |
| 0 | Vvi-Vitvi05g02184\_t001 |  |  |  |  |  |  |  |  |  |  |  |  |
| 0 | Vvi-Vitvi05g02185\_t001 |  |  |  |  |  |  |  |  |  |  |  |  |
| 0 | Vvi-Vitvi05g04455\_t001 |  |  |  |  |  |  |  |  |  |  |  |  |
| 0 | Vvi-Vitvi05g02187\_t001 |  |  |  |  |  |  |  |  |  |  |  |  |
| 0 | Vvi-Vitvi05g04456\_t001 |  |  |  |  |  |  |  |  |  |  |  |  |
| 0 | Vvi-Vitvi05g04457\_t001 |  |  |  |  |  |  |  |  |  |  |  |  |
| 0 | Vvi-Vitvi05g01510\_t001 |  |  |  |  |  |  |  |  |  |  |  |  |
| 0 | Vvi-Vitvi05g01511\_t001 |  |  |  |  |  |  |  |  |  |  |  |  |
| 0 | Vvi-Vitvi05g02190\_t001 |  |  |  |  |  |  |  |  |  |  |  |  |
| 0 | Vvi-Vitvi05g04458\_t001 |  |  |  |  |  |  |  |  |  |  |  |  |
| 0 | Vvi-Vitvi05g02192\_t001 |  |  |  |  |  |  |  |  |  |  |  |  |
| 0 | Vvi-Vitvi05g01514\_t001 |  |  |  |  |  |  |  |  |  |  |  |  |
| 0 | Vvi-Vitvi05g01516\_t001 |  |  |  |  |  |  |  |  |  |  |  |  |
| 0 | Vvi-Vitvi05g01517\_t001 |  |  |  |  |  |  |  |  |  |  |  |  |
| 0 | Vvi-Vitvi05g01518\_t001 |  |  |  |  |  |  |  |  |  |  |  |  |
| 0 | Vvi-Vitvi05g04459\_t001 |  |  |  |  |  |  |  |  |  |  |  |  |
| 0 | Vvi-Vitvi05g02193\_t001 |  |  |  |  |  |  |  |  |  |  |  |  |
| 0 | Vvi-Vitvi05g01519\_t001 |  |  |  |  |  |  |  |  |  |  |  |  |
| 0 | Vvi-Vitvi05g04460\_t001 |  |  |  |  |  |  |  |  |  |  |  |  |
| 0 | Vvi-Vitvi05g01520\_t001 |  |  |  |  |  |  |  |  |  |  |  |  |
| 0 | Vvi-Vitvi05g04461\_t001 |  |  |  |  |  |  |  |  |  |  |  |  |
| 0 | Vvi-Vitvi05g02194\_t001 |  |  |  |  |  |  |  |  |  |  |  |  |
| 0 | Vvi-Vitvi05g04462\_t001 |  |  |  |  |  |  |  |  |  |  |  |  |
| 0 | Vvi-Vitvi05g04463\_t001 |  |  |  |  |  |  |  |  |  |  |  |  |
| 0 | Vvi-Vitvi05g04464\_t001 |  |  |  |  |  |  |  |  |  |  |  |  |
| 0 | Vvi-Vitvi05g04465\_t001 |  |  |  |  |  |  |  |  |  |  |  |  |
| 0 | Vvi-Vitvi05g02195\_t001 |  |  |  |  |  |  |  |  |  |  |  |  |
| 0 | Vvi-Vitvi05g04466\_t001 |  |  |  |  |  |  |  |  |  |  |  |  |
| 0 | Vvi-Vitvi05g04467\_t001 |  |  |  |  |  |  |  |  |  |  |  |  |
| 0 | Vvi-Vitvi05g04468\_t001 |  |  |  |  |  |  |  |  |  |  |  |  |
| 0 | Vvi-Vitvi05g04469\_t001 |  |  |  |  |  |  |  |  |  |  |  |  |
| 0 | Vvi-Vitvi05g04470\_t001 |  |  |  |  |  |  |  |  |  |  |  |  |
| 0 | Vvi-Vitvi05g02197\_t001 |  |  |  |  |  |  |  |  |  |  |  |  |
| 0 | Vvi-Vitvi05g04471\_t001 |  |  |  |  |  |  |  |  |  |  |  |  |
| 0 | Vvi-Vitvi05g02198\_t001 |  |  |  |  |  |  |  |  |  |  |  |  |
| 0 | Vvi-Vitvi05g04472\_t001 |  |  |  |  |  |  |  |  |  |  |  |  |
| 0 | Vvi-Vitvi05g04473\_t001 |  |  |  |  |  |  |  |  |  |  |  |  |
| 0 | Vvi-Vitvi05g02200\_t001 |  |  |  |  |  |  |  |  |  |  |  |  |
| 0 | Vvi-Vitvi05g04474\_t001 |  |  |  |  |  |  |  |  |  |  |  |  |
| 0 | Vvi-Vitvi05g02201\_t001 |  |  |  |  |  |  |  |  |  |  |  |  |
| 0 | Vvi-Vitvi05g04475\_t001 |  |  |  |  |  |  |  |  |  |  |  |  |
| 0 | Vvi-Vitvi05g04476\_t001 |  |  |  |  |  |  |  |  |  |  |  |  |
| 0 | Vvi-Vitvi05g04477\_t001 |  |  |  |  |  |  |  |  |  |  |  |  |
| 0 | Vvi-Vitvi05g04478\_t001 |  |  |  |  |  |  |  |  |  |  |  |  |
| 0 | Vvi-Vitvi05g01522\_t001 |  |  |  |  |  |  |  |  |  |  |  |  |
| 0 | Vvi-Vitvi05g04479\_t001 |  |  |  |  |  |  |  |  |  |  |  |  |
| 0 | Vvi-Vitvi05g04480\_t001 |  |  |  |  |  |  |  |  |  |  |  |  |
| 0 | Vvi-Vitvi05g02203\_t001 |  |  |  |  |  |  |  |  |  |  |  |  |
| 0 | Vvi-Vitvi05g04481\_t001 |  |  |  |  |  |  |  |  |  |  |  |  |
| 0 | Vvi-Vitvi05g04482\_t001 |  |  |  |  |  |  |  |  |  |  |  |  |
| 0 | Vvi-Vitvi05g04483\_t001 |  |  |  |  |  |  |  |  |  |  |  |  |
| 0 | Vvi-Vitvi05g02204\_t001 |  |  |  |  |  |  |  |  |  |  |  |  |
| 0 | Vvi-Vitvi05g01524\_t001 |  |  |  |  |  |  |  |  |  |  |  |  |
| 0 | Vvi-Vitvi05g02205\_t001 |  |  |  |  |  |  |  |  |  |  |  |  |
| 0 | Vvi-Vitvi05g02206\_t001 |  |  |  |  |  |  |  |  |  |  |  |  |
| 0 | Vvi-Vitvi05g02212\_t001 |  |  |  |  |  |  |  |  |  |  |  |  |
| 0 | Vvi-Vitvi05g04484\_t001 |  |  |  |  |  |  |  |  |  |  |  |  |
| 0 | Vvi-Vitvi05g02297\_t001 |  |  |  |  |  |  |  |  |  |  |  |  |
| 0 | Vvi-Vitvi05g02208\_t001 |  |  |  |  |  |  |  |  |  |  |  |  |
| 0 | Vvi-Vitvi05g04485\_t001 |  |  |  |  |  |  |  |  |  |  |  |  |
| 0 | Vvi-Vitvi05g04486\_t001 |  |  |  |  |  |  |  |  |  |  |  |  |
| 0 | Vvi-Vitvi05g01528\_t001 |  |  |  |  |  |  |  |  |  |  |  |  |
| 0 | Vvi-Vitvi05g04487\_t001 |  |  |  |  |  |  |  |  |  |  |  |  |
| 0 | Vvi-Vitvi05g02210\_t001 |  |  |  |  |  |  |  |  |  |  |  |  |
| 0 | Vvi-Vitvi05g04488\_t001 |  |  |  |  |  |  |  |  |  |  |  |  |
| 0 | Vvi-Vitvi05g02213\_t001 |  |  |  |  |  |  |  |  |  |  |  |  |
| 0 | Vvi-Vitvi05g04489\_t001 |  |  |  |  |  |  |  |  |  |  |  |  |
| 0 | Vvi-Vitvi05g04490\_t001 |  |  |  |  |  |  |  |  |  |  |  |  |
| 0 | Vvi-Vitvi05g04491\_t001 |  |  |  |  |  |  |  |  |  |  |  |  |
| 0 | Vvi-Vitvi05g01530\_t001 |  |  |  |  |  |  |  |  |  |  |  |  |
| 0 | Vvi-Vitvi05g04492\_t001 |  |  |  |  |  |  |  |  |  |  |  |  |
| 0 | Vvi-Vitvi05g04493\_t001 |  |  |  |  |  |  |  |  |  |  |  |  |
| 0 | Vvi-Vitvi05g04494\_t001 |  |  |  |  |  |  |  |  |  |  |  |  |
| 0 | Vvi-Vitvi05g04495\_t001 |  |  |  |  |  |  |  |  |  |  |  |  |
| 0 | Vvi-Vitvi05g04496\_t001 |  |  |  |  |  |  |  |  |  |  |  |  |
| 0 | Vvi-Vitvi05g04497\_t001 |  |  |  |  |  |  |  |  |  |  |  |  |
| 0 | Vvi-Vitvi05g04498\_t001 |  |  |  |  |  |  |  |  |  |  |  |  |
| 0 | Vvi-Vitvi05g04499\_t001 |  |  |  |  |  |  |  |  |  |  |  |  |
| 0 | Vvi-Vitvi05g02215\_t001 |  |  |  |  |  |  |  |  |  |  |  |  |
| 0 | Vvi-Vitvi05g04500\_t001 |  |  |  |  |  |  |  |  |  |  |  |  |
| 0 | Vvi-Vitvi05g02217\_t001 |  |  |  |  |  |  |  |  |  |  |  |  |
| 0 | Vvi-Vitvi05g04501\_t001 |  |  |  |  |  |  |  |  |  |  |  |  |
| 0 | Vvi-Vitvi05g04502\_t001 |  |  |  |  |  |  |  |  |  |  |  |  |
| 0 | Vvi-Vitvi05g02219\_t001 |  |  |  |  |  |  |  |  |  |  |  |  |
| 0 | Vvi-Vitvi05g01533\_t001 |  |  |  |  |  |  |  |  |  |  |  |  |
| 0 | Vvi-Vitvi05g02221\_t001 |  |  |  |  |  |  |  |  |  |  |  |  |
| 0 | Vvi-Vitvi05g02222\_t001 |  |  |  |  |  |  |  |  |  |  |  |  |
| 0 | Vvi-Vitvi05g02223\_t001 |  |  |  |  |  |  |  |  |  |  |  |  |
| 0 | Vvi-Vitvi05g02224\_t001 |  |  |  |  |  |  |  |  |  |  |  |  |
| 0 | Vvi-Vitvi05g04503\_t001 |  |  |  |  |  |  |  |  |  |  |  |  |
| 0 | Vvi-Vitvi05g04504\_t001 |  |  |  |  |  |  |  |  |  |  |  |  |
| 0 | Vvi-Vitvi05g02228\_t001 |  |  |  |  |  |  |  |  |  |  |  |  |
| 0 | Vvi-Vitvi05g04505\_t001 |  |  |  |  |  |  |  |  |  |  |  |  |
| 0 | Vvi-Vitvi05g04506\_t001 |  |  |  |  |  |  |  |  |  |  |  |  |
| 0 | Vvi-Vitvi05g02232\_t001 |  |  |  |  |  |  |  |  |  |  |  |  |
| 1 | Vvi-Vitvi05g01536\_t001 |  | Atr-ERN20328 |  |  |  |  |  |  |  |  |  |  |
| 1 | Vvi-Vitvi05g04507\_t001 |  | \| \| |  |  |  |  |  |  |  |  |  |  |
| 1 | Vvi-Vitvi05g01537\_t001 |  | Atr-ERN20326 |  |  |  |  |  |  |  |  |  |  |
| 1 | Vvi-Vitvi05g04508\_t001 |  | \| \| |  |  |  |  |  |  |  |  |  |  |
| 1 | Vvi-Vitvi05g02233\_t001 |  | \| \| |  |  |  |  |  |  |  |  |  |  |
| 1 | Vvi-Vitvi05g02234\_t001 |  | \| \| |  |  |  |  |  |  |  |  |  |  |
| 1 | Vvi-Vitvi05g01540\_t003 |  | Atr-ERN20323 |  |  |  |  |  |  |  |  |  |  |
| 1 | Vvi-Vitvi05g01542\_t001 |  | Atr-ERN20322 |  |  |  |  |  |  |  |  |  |  |
| 1 | Vvi-Vitvi05g01543\_t001 |  | Atr-ERN20321 |  |  |  |  |  |  |  |  |  |  |
| 1 | Vvi-Vitvi05g01544\_t001 |  | Atr-ERN20320 |  |  |  |  |  |  |  |  |  |  |
| 1 | Vvi-Vitvi05g04509\_t001 |  | \| \| |  |  |  |  |  |  |  |  |  |  |
| 1 | Vvi-Vitvi05g01545\_t001 |  | Atr-ERN20319 |  |  |  |  |  |  |  |  |  |  |
| 1 | Vvi-Vitvi05g01546\_t001 |  | Atr-ERN20318 |  |  |  |  |  |  |  |  |  |  |
| 1 | Vvi-Vitvi05g01547\_t001 |  | \| \| |  |  |  |  |  |  |  |  |  |  |
| 1 | Vvi-Vitvi05g01548\_t001 |  | \| \| |  |  |  |  |  |  |  |  |  |  |
| 1 | Vvi-Vitvi05g04510\_t002 |  | \| \| |  |  |  |  |  |  |  |  |  |  |
| 1 | Vvi-Vitvi05g01550\_t001 |  | \| \| |  |  |  |  |  |  |  |  |  |  |
| 2 | Vvi-Vitvi05g01551\_t001 |  | \| \| |  | Atr-ERN20306 |  |  |  |  |  |  |  |  |
| 2 | Vvi-Vitvi05g04511\_t001 |  | \| \| |  | \| \| |  |  |  |  |  |  |  |  |
| 2 | Vvi-Vitvi05g02235\_t001 |  | \| \| |  | \| \| |  |  |  |  |  |  |  |  |
| 2 | Vvi-Vitvi05g02236\_t001 |  | \| \| |  | \| \| |  |  |  |  |  |  |  |  |
| 2 | Vvi-Vitvi05g01553\_t001 |  | \| \| |  | Atr-ERN20310 |  |  |  |  |  |  |  |  |
| 2 | Vvi-Vitvi05g01554\_t001 |  | \| \| |  | Atr-ERN20313 |  |  |  |  |  |  |  |  |
| 2 | Vvi-Vitvi05g02237\_t001 |  | \| \| |  | \| \| |  |  |  |  |  |  |  |  |
| 2 | Vvi-Vitvi05g01555\_t001 |  | Atr-ERN20308 |  | \| \| |  |  |  |  |  |  |  |  |
| 2 | Vvi-Vitvi05g01556\_t001 |  | Atr-ERN20307 |  | \| \| |  |  |  |  |  |  |  |  |
| 2 | Vvi-Vitvi05g04512\_t001 |  | Atr-ERN20306 |  | \| \| |  |  |  |  |  |  |  |  |
| 1 | Vvi-Vitvi05g01558\_t001 |  |  |  | \| \| |  |  |  |  |  |  |  |  |
| 1 | Vvi-Vitvi05g04513\_t001 |  |  |  | \| \| |  |  |  |  |  |  |  |  |
| 1 | Vvi-Vitvi05g04514\_t001 |  |  |  | \| \| |  |  |  |  |  |  |  |  |
| 1 | Vvi-Vitvi05g01560\_t001 |  |  |  | \| \| |  |  |  |  |  |  |  |  |
| 1 | Vvi-Vitvi05g04515\_t001 |  |  |  | \| \| |  |  |  |  |  |  |  |  |
| 1 | Vvi-Vitvi05g04516\_t001 |  |  |  | Atr-ERN20336 |  |  |  |  |  |  |  |  |
| 1 | Vvi-Vitvi05g01563\_t001 |  |  |  | \| \| |  |  |  |  |  |  |  |  |
| 1 | Vvi-Vitvi05g01564\_t001 |  |  |  | Atr-ERN20343 |  |  |  |  |  |  |  |  |
| 1 | Vvi-Vitvi05g04517\_t001 |  |  |  | Atr-ERN20344 |  |  |  |  |  |  |  |  |
| 1 | Vvi-Vitvi05g02239\_t001 |  |  |  | Atr-ERN20345 |  |  |  |  |  |  |  |  |
| 1 | Vvi-Vitvi05g01566\_t001 |  |  |  | Atr-ERN20346 |  |  |  |  |  |  |  |  |
| 1 | Vvi-Vitvi05g04518\_t001 |  |  |  | \| \| |  |  |  |  |  |  |  |  |
| 1 | Vvi-Vitvi05g04519\_t001 |  |  |  | \| \| |  |  |  |  |  |  |  |  |
| 1 | Vvi-Vitvi05g04520\_t001 |  |  |  | \| \| |  |  |  |  |  |  |  |  |
| 1 | Vvi-Vitvi05g04521\_t001 |  |  |  | \| \| |  |  |  |  |  |  |  |  |
| 1 | Vvi-Vitvi05g01568\_t001 |  |  |  | \| \| |  |  |  |  |  |  |  |  |
| 1 | Vvi-Vitvi05g04522\_t001 |  |  |  | \| \| |  |  |  |  |  |  |  |  |
| 1 | Vvi-Vitvi05g01569\_t001 |  |  |  | \| \| |  |  |  |  |  |  |  |  |
| 1 | Vvi-Vitvi05g04523\_t001 |  |  |  | \| \| |  |  |  |  |  |  |  |  |
| 1 | Vvi-Vitvi05g04524\_t001 |  |  |  | \| \| |  |  |  |  |  |  |  |  |
| 1 | Vvi-Vitvi05g04525\_t001 |  |  |  | \| \| |  |  |  |  |  |  |  |  |
| 1 | Vvi-Vitvi05g01572\_t001 |  |  |  | Atr-ERN20348 |  |  |  |  |  |  |  |  |
| 1 | Vvi-Vitvi05g04526\_t001 |  |  |  | \| \| |  |  |  |  |  |  |  |  |
| 1 | Vvi-Vitvi05g02241\_t001 |  |  |  | \| \| |  |  |  |  |  |  |  |  |
| 1 | Vvi-Vitvi05g04527\_t001 |  |  |  | \| \| |  |  |  |  |  |  |  |  |
| 1 | Vvi-Vitvi05g01573\_t001 |  |  |  | \| \| |  |  |  |  |  |  |  |  |
| 1 | Vvi-Vitvi05g01574\_t001 |  |  |  | \| \| |  |  |  |  |  |  |  |  |
| 1 | Vvi-Vitvi05g02244\_t001 |  |  |  | \| \| |  |  |  |  |  |  |  |  |
| 1 | Vvi-Vitvi05g01575\_t001 |  |  |  | \| \| |  |  |  |  |  |  |  |  |
| 1 | Vvi-Vitvi05g04528\_t001 |  |  |  | \| \| |  |  |  |  |  |  |  |  |
| 1 | Vvi-Vitvi05g01577\_t001 |  |  |  | \| \| |  |  |  |  |  |  |  |  |
| 1 | Vvi-Vitvi05g02245\_t001 |  |  |  | \| \| |  |  |  |  |  |  |  |  |
| 1 | Vvi-Vitvi05g04529\_t001 |  |  |  | \| \| |  |  |  |  |  |  |  |  |
| 1 | Vvi-Vitvi05g01578\_t001 |  |  |  | \| \| |  |  |  |  |  |  |  |  |
| 1 | Vvi-Vitvi05g01579\_t001 |  |  |  | \| \| |  |  |  |  |  |  |  |  |
| 1 | Vvi-Vitvi05g02248\_t001 |  |  |  | \| \| |  |  |  |  |  |  |  |  |
| 1 | Vvi-Vitvi05g01580\_t001 |  |  |  | \| \| |  |  |  |  |  |  |  |  |
| 1 | Vvi-Vitvi05g01581\_t001 |  |  |  | \| \| |  |  |  |  |  |  |  |  |
| 1 | Vvi-Vitvi05g04530\_t001 |  |  |  | \| \| |  |  |  |  |  |  |  |  |
| 1 | Vvi-Vitvi05g04531\_t001 |  |  |  | \| \| |  |  |  |  |  |  |  |  |
| 1 | Vvi-Vitvi05g04532\_t001 |  |  |  | \| \| |  |  |  |  |  |  |  |  |
| 1 | Vvi-Vitvi05g04533\_t001 |  |  |  | \| \| |  |  |  |  |  |  |  |  |
| 1 | Vvi-Vitvi05g04534\_t001 |  |  |  | \| \| |  |  |  |  |  |  |  |  |
| 1 | Vvi-Vitvi05g02249\_t001 |  |  |  | Atr-ERN20350 |  |  |  |  |  |  |  |  |
| 1 | Vvi-Vitvi05g01583\_t001 |  |  |  | \| \| |  |  |  |  |  |  |  |  |
| 1 | Vvi-Vitvi05g04535\_t001 |  |  |  | \| \| |  |  |  |  |  |  |  |  |
| 1 | Vvi-Vitvi05g02251\_t001 |  |  |  | \| \| |  |  |  |  |  |  |  |  |
| 1 | Vvi-Vitvi05g01584\_t001 |  |  |  | Atr-ERN20352 |  |  |  |  |  |  |  |  |
| 1 | Vvi-Vitvi05g02252\_t003 |  |  |  | \| \| |  |  |  |  |  |  |  |  |
| 1 | Vvi-Vitvi05g01585\_t001 |  |  |  | Atr-ERN20354 |  |  |  |  |  |  |  |  |
| 1 | Vvi-Vitvi05g01587\_t001 |  |  |  | \| \| |  |  |  |  |  |  |  |  |
| 1 | Vvi-Vitvi05g01588\_t001 |  |  |  | \| \| |  |  |  |  |  |  |  |  |
| 1 | Vvi-Vitvi05g01589\_t001 |  |  |  | \| \| |  |  |  |  |  |  |  |  |
| 1 | Vvi-Vitvi05g01590\_t001 |  |  |  | \| \| |  |  |  |  |  |  |  |  |
| 1 | Vvi-Vitvi05g01592\_t001 |  |  |  | Atr-ERN20357 |  |  |  |  |  |  |  |  |
| 1 | Vvi-Vitvi05g01593\_t002 |  |  |  | \| \| |  |  |  |  |  |  |  |  |
| 1 | Vvi-Vitvi05g01594\_t002 |  |  |  | \| \| |  |  |  |  |  |  |  |  |
| 1 | Vvi-Vitvi05g01595\_t003 |  |  |  | \| \| |  |  |  |  |  |  |  |  |
| 1 | Vvi-Vitvi05g01596\_t001 |  |  |  | Atr-ERN20358 |  |  |  |  |  |  |  |  |
| 1 | Vvi-Vitvi05g01597\_t001 |  |  |  | \| \| |  |  |  |  |  |  |  |  |
| 1 | Vvi-Vitvi05g01599\_t001 |  |  |  | Atr-ERN20359 |  |  |  |  |  |  |  |  |
| 0 | Vvi-Vitvi05g01600\_t001 |  |  |  |  |  |  |  |  |  |  |  |  |
| 0 | Vvi-Vitvi05g01601\_t001 |  |  |  |  |  |  |  |  |  |  |  |  |
| 0 | Vvi-Vitvi05g01602\_t001 |  |  |  |  |  |  |  |  |  |  |  |  |
| 0 | Vvi-Vitvi05g01603\_t001 |  |  |  |  |  |  |  |  |  |  |  |  |
| 0 | Vvi-Vitvi05g04536\_t001 |  |  |  |  |  |  |  |  |  |  |  |  |
| 0 | Vvi-Vitvi05g02253\_t001 |  |  |  |  |  |  |  |  |  |  |  |  |
| 0 | Vvi-Vitvi05g04537\_t001 |  |  |  |  |  |  |  |  |  |  |  |  |
| 0 | Vvi-Vitvi05g04538\_t001 |  |  |  |  |  |  |  |  |  |  |  |  |
| 0 | Vvi-Vitvi05g01605\_t001 |  |  |  |  |  |  |  |  |  |  |  |  |
| 0 | Vvi-Vitvi05g01607\_t001 |  |  |  |  |  |  |  |  |  |  |  |  |
| 0 | Vvi-Vitvi05g01609\_t001 |  |  |  |  |  |  |  |  |  |  |  |  |
| 0 | Vvi-Vitvi05g02257\_t001 |  |  |  |  |  |  |  |  |  |  |  |  |
| 0 | Vvi-Vitvi05g04539\_t001 |  |  |  |  |  |  |  |  |  |  |  |  |
| 0 | Vvi-Vitvi05g04540\_t001 |  |  |  |  |  |  |  |  |  |  |  |  |
| 0 | Vvi-Vitvi05g01610\_t001 |  |  |  |  |  |  |  |  |  |  |  |  |
| 0 | Vvi-Vitvi05g04541\_t001 |  |  |  |  |  |  |  |  |  |  |  |  |
| 0 | Vvi-Vitvi05g01611\_t001 |  |  |  |  |  |  |  |  |  |  |  |  |
| 0 | Vvi-Vitvi05g01612\_t001 |  |  |  |  |  |  |  |  |  |  |  |  |
| 0 | Vvi-Vitvi05g04542\_t001 |  |  |  |  |  |  |  |  |  |  |  |  |
| 0 | Vvi-Vitvi05g01616\_t001 |  |  |  |  |  |  |  |  |  |  |  |  |
| 0 | Vvi-Vitvi05g04543\_t001 |  |  |  |  |  |  |  |  |  |  |  |  |
| 0 | Vvi-Vitvi05g04544\_t001 |  |  |  |  |  |  |  |  |  |  |  |  |
| 0 | Vvi-Vitvi05g04545\_t001 |  |  |  |  |  |  |  |  |  |  |  |  |
| 0 | Vvi-Vitvi05g04546\_t001 |  |  |  |  |  |  |  |  |  |  |  |  |
| 0 | Vvi-Vitvi05g04547\_t001 |  |  |  |  |  |  |  |  |  |  |  |  |
| 0 | Vvi-Vitvi05g04548\_t001 |  |  |  |  |  |  |  |  |  |  |  |  |
| 0 | Vvi-Vitvi05g04549\_t001 |  |  |  |  |  |  |  |  |  |  |  |  |
| 0 | Vvi-Vitvi05g01618\_t001 |  |  |  |  |  |  |  |  |  |  |  |  |
| 0 | Vvi-Vitvi05g01619\_t001 |  |  |  |  |  |  |  |  |  |  |  |  |
| 0 | Vvi-Vitvi05g01620\_t001 |  |  |  |  |  |  |  |  |  |  |  |  |
| 0 | Vvi-Vitvi05g01621\_t001 |  |  |  |  |  |  |  |  |  |  |  |  |
| 1 | Vvi-Vitvi05g01622\_t001 |  | Atr-ERN20250 |  |  |  |  |  |  |  |  |  |  |
| 1 | Vvi-Vitvi05g01623\_t001 |  | Atr-ERN20251 |  |  |  |  |  |  |  |  |  |  |
| 1 | Vvi-Vitvi05g01624\_t001 |  | Atr-ERN20252 |  |  |  |  |  |  |  |  |  |  |
| 1 | Vvi-Vitvi05g01626\_t001 |  | Atr-ERN20255 |  |  |  |  |  |  |  |  |  |  |
| 1 | Vvi-Vitvi05g01627\_t001 |  | Atr-ERN20256 |  |  |  |  |  |  |  |  |  |  |
| 1 | Vvi-Vitvi05g01629\_t001 |  | Atr-ERN20257 |  |  |  |  |  |  |  |  |  |  |
| 1 | Vvi-Vitvi05g01630\_t001 |  | \| \| |  |  |  |  |  |  |  |  |  |  |
| 1 | Vvi-Vitvi05g01631\_t001 |  | \| \| |  |  |  |  |  |  |  |  |  |  |
| 1 | Vvi-Vitvi05g01632\_t001 |  | Atr-ERN20259 |  |  |  |  |  |  |  |  |  |  |
| 1 | Vvi-Vitvi05g01633\_t001 |  | \| \| |  |  |  |  |  |  |  |  |  |  |
| 1 | Vvi-Vitvi05g02262\_t001 |  | Atr-ERN20275 |  |  |  |  |  |  |  |  |  |  |
| 1 | Vvi-Vitvi05g01634\_t001 |  | \| \| |  |  |  |  |  |  |  |  |  |  |
| 1 | Vvi-Vitvi05g01636\_t001 |  | Atr-ERN20280 |  |  |  |  |  |  |  |  |  |  |
| 1 | Vvi-Vitvi05g01638\_t001 |  | Atr-ERN20287 |  |  |  |  |  |  |  |  |  |  |
| 1 | Vvi-Vitvi05g01639\_t001 |  | Atr-ERN20288 |  |  |  |  |  |  |  |  |  |  |
| 1 | Vvi-Vitvi05g01640\_t001 |  | \| \| |  |  |  |  |  |  |  |  |  |  |
| 1 | Vvi-Vitvi05g01641\_t001 |  | Atr-ERN20291 |  |  |  |  |  |  |  |  |  |  |
| 1 | Vvi-Vitvi05g04550\_t001 |  | \| \| |  |  |  |  |  |  |  |  |  |  |
| 1 | Vvi-Vitvi05g01642\_t001 |  | Atr-ERN20293 |  |  |  |  |  |  |  |  |  |  |
| 1 | Vvi-Vitvi05g04551\_t001 |  | \| \| |  |  |  |  |  |  |  |  |  |  |
| 1 | Vvi-Vitvi05g01643\_t001 |  | \| \| |  |  |  |  |  |  |  |  |  |  |
| 1 | Vvi-Vitvi05g04552\_t001 |  | \| \| |  |  |  |  |  |  |  |  |  |  |
| 1 | Vvi-Vitvi05g01647\_t001 |  | \| \| |  |  |  |  |  |  |  |  |  |  |
| 1 | Vvi-Vitvi05g01648\_t001 |  | Atr-ERN20295 |  |  |  |  |  |  |  |  |  |  |
| 1 | Vvi-Vitvi05g04553\_t001 |  | Atr-ERN20298 |  |  |  |  |  |  |  |  |  |  |
| 1 | Vvi-Vitvi05g01649\_t001 |  | \| \| |  |  |  |  |  |  |  |  |  |  |
| 1 | Vvi-Vitvi05g04554\_t001 |  | Atr-ERN20300 |  |  |  |  |  |  |  |  |  |  |
| 1 | Vvi-Vitvi05g01650\_t001 |  | \| \| |  |  |  |  |  |  |  |  |  |  |
| 1 | Vvi-Vitvi05g04555\_t001 |  | \| \| |  |  |  |  |  |  |  |  |  |  |
| 1 | Vvi-Vitvi05g02266\_t001 |  | Atr-ERN20304 |  |  |  |  |  |  |  |  |  |  |
| 0 | Vvi-Vitvi05g04556\_t001 |  |  |  |  |  |  |  |  |  |  |  |  |
| 0 | Vvi-Vitvi05g01652\_t001 |  |  |  |  |  |  |  |  |  |  |  |  |
| 0 | Vvi-Vitvi05g04557\_t001 |  |  |  |  |  |  |  |  |  |  |  |  |
| 0 | Vvi-Vitvi05g04558\_t001 |  |  |  |  |  |  |  |  |  |  |  |  |
| 0 | Vvi-Vitvi05g04559\_t001 |  |  |  |  |  |  |  |  |  |  |  |  |
| 0 | Vvi-Vitvi05g04560\_t001 |  |  |  |  |  |  |  |  |  |  |  |  |
| 0 | Vvi-Vitvi05g04561\_t001 |  |  |  |  |  |  |  |  |  |  |  |  |
| 0 | Vvi-Vitvi05g01654\_t001 |  |  |  |  |  |  |  |  |  |  |  |  |
| 1 | Vvi-Vitvi05g01655\_t001 |  | Atr-ERN20231 |  |  |  |  |  |  |  |  |  |  |
| 1 | Vvi-Vitvi05g04562\_t001 |  | \| \| |  |  |  |  |  |  |  |  |  |  |
| 1 | Vvi-Vitvi05g01656\_t001 |  | Atr-ERN20227 |  |  |  |  |  |  |  |  |  |  |
| 1 | Vvi-Vitvi05g04563\_t001 |  | \| \| |  |  |  |  |  |  |  |  |  |  |
| 1 | Vvi-Vitvi05g01658\_t001 |  | \| \| |  |  |  |  |  |  |  |  |  |  |
| 1 | Vvi-Vitvi05g04564\_t001 |  | \| \| |  |  |  |  |  |  |  |  |  |  |
| 1 | Vvi-Vitvi05g01659\_t001 |  | \| \| |  |  |  |  |  |  |  |  |  |  |
| 1 | Vvi-Vitvi05g01660\_t001 |  | Atr-ERN20225 |  |  |  |  |  |  |  |  |  |  |
| 1 | Vvi-Vitvi05g01661\_t001 |  | \| \| |  |  |  |  |  |  |  |  |  |  |
| 1 | Vvi-Vitvi05g02267\_t001 |  | \| \| |  |  |  |  |  |  |  |  |  |  |
| 1 | Vvi-Vitvi05g01662\_t001 |  | \| \| |  |  |  |  |  |  |  |  |  |  |
| 1 | Vvi-Vitvi05g01663\_t001 |  | \| \| |  |  |  |  |  |  |  |  |  |  |
| 1 | Vvi-Vitvi05g02268\_t001 |  | \| \| |  |  |  |  |  |  |  |  |  |  |
| 1 | Vvi-Vitvi05g04565\_t001 |  | Atr-ERN20218 |  |  |  |  |  |  |  |  |  |  |
| 1 | Vvi-Vitvi05g01665\_t001 |  | \| \| |  |  |  |  |  |  |  |  |  |  |
| 1 | Vvi-Vitvi05g01666\_t001 |  | \| \| |  |  |  |  |  |  |  |  |  |  |
| 1 | Vvi-Vitvi05g04566\_t001 |  | \| \| |  |  |  |  |  |  |  |  |  |  |
| 1 | Vvi-Vitvi05g02269\_t001 |  | \| \| |  |  |  |  |  |  |  |  |  |  |
| 1 | Vvi-Vitvi05g04567\_t001 |  | \| \| |  |  |  |  |  |  |  |  |  |  |
| 1 | Vvi-Vitvi05g04568\_t001 |  | \| \| |  |  |  |  |  |  |  |  |  |  |
| 1 | Vvi-Vitvi05g04569\_t001 |  | \| \| |  |  |  |  |  |  |  |  |  |  |
| 1 | Vvi-Vitvi05g01670\_t001 |  | \| \| |  |  |  |  |  |  |  |  |  |  |
| 1 | Vvi-Vitvi05g04570\_t001 |  | \| \| |  |  |  |  |  |  |  |  |  |  |
| 1 | Vvi-Vitvi05g04571\_t001 |  | \| \| |  |  |  |  |  |  |  |  |  |  |
| 1 | Vvi-Vitvi05g01672\_t001 |  | Atr-ERN20216 |  |  |  |  |  |  |  |  |  |  |
| 1 | Vvi-Vitvi05g01673\_t003 |  | \| \| |  |  |  |  |  |  |  |  |  |  |
| 1 | Vvi-Vitvi05g01674\_t001 |  | \| \| |  |  |  |  |  |  |  |  |  |  |
| 1 | Vvi-Vitvi05g01675\_t001 |  | \| \| |  |  |  |  |  |  |  |  |  |  |
| 1 | Vvi-Vitvi05g01676\_t001 |  | \| \| |  |  |  |  |  |  |  |  |  |  |
| 1 | Vvi-Vitvi05g02271\_t001 |  | \| \| |  |  |  |  |  |  |  |  |  |  |
| 1 | Vvi-Vitvi05g01677\_t001 |  | \| \| |  |  |  |  |  |  |  |  |  |  |
| 1 | Vvi-Vitvi05g01678\_t001 |  | \| \| |  |  |  |  |  |  |  |  |  |  |
| 1 | Vvi-Vitvi05g04572\_t001 |  | \| \| |  |  |  |  |  |  |  |  |  |  |
| 1 | Vvi-Vitvi05g01679\_t001 |  | Atr-ERN20214 |  |  |  |  |  |  |  |  |  |  |
| 1 | Vvi-Vitvi05g01681\_t001 |  | \| \| |  |  |  |  |  |  |  |  |  |  |
| 1 | Vvi-Vitvi05g01682\_t001 |  | \| \| |  |  |  |  |  |  |  |  |  |  |
| 1 | Vvi-Vitvi05g01683\_t001 |  | \| \| |  |  |  |  |  |  |  |  |  |  |
| 1 | Vvi-Vitvi05g02272\_t001 |  | Atr-ERN20208 |  |  |  |  |  |  |  |  |  |  |
| 1 | Vvi-Vitvi05g02273\_t001 |  | \| \| |  |  |  |  |  |  |  |  |  |  |
| 1 | Vvi-Vitvi05g04573\_t001 |  | \| \| |  |  |  |  |  |  |  |  |  |  |
| 1 | Vvi-Vitvi05g01684\_t001 |  | \| \| |  |  |  |  |  |  |  |  |  |  |
| 1 | Vvi-Vitvi05g02275\_t002 |  | \| \| |  |  |  |  |  |  |  |  |  |  |
| 1 | Vvi-Vitvi05g04574\_t001 |  | \| \| |  |  |  |  |  |  |  |  |  |  |
| 1 | Vvi-Vitvi05g01685\_t001 |  | Atr-ERN20204 |  |  |  |  |  |  |  |  |  |  |
| 1 | Vvi-Vitvi05g02276\_t001 |  | Atr-ERN20202 |  |  |  |  |  |  |  |  |  |  |
| 1 | Vvi-Vitvi05g01686\_t001 |  | Atr-ERN20195 |  |  |  |  |  |  |  |  |  |  |
| 1 | Vvi-Vitvi05g01687\_t001 |  | Atr-ERN20190 |  |  |  |  |  |  |  |  |  |  |
| 1 | Vvi-Vitvi05g01688\_t001 |  | Atr-ERN20189 |  |  |  |  |  |  |  |  |  |  |
| 1 | Vvi-Vitvi05g04575\_t001 |  | Atr-ERN20186 |  |  |  |  |  |  |  |  |  |  |
| 1 | Vvi-Vitvi05g02278\_t001 |  | Atr-ERN20184 |  |  |  |  |  |  |  |  |  |  |
| 1 | Vvi-Vitvi05g01689\_t001 |  | Atr-ERN20180 |  |  |  |  |  |  |  |  |  |  |
| 1 | Vvi-Vitvi05g01690\_t001 |  | Atr-ERN20178 |  |  |  |  |  |  |  |  |  |  |
| 1 | Vvi-Vitvi05g01691\_t001 |  | Atr-ERN20176 |  |  |  |  |  |  |  |  |  |  |
| 1 | Vvi-Vitvi05g01692\_t001 |  | Atr-ERN20173 |  |  |  |  |  |  |  |  |  |  |
| 1 | Vvi-Vitvi05g01693\_t001 |  | Atr-ERN20172 |  |  |  |  |  |  |  |  |  |  |
| 1 | Vvi-Vitvi05g01694\_t001 |  | Atr-ERN20171 |  |  |  |  |  |  |  |  |  |  |
| 1 | Vvi-Vitvi05g01696\_t001 |  | Atr-ERN20167 |  |  |  |  |  |  |  |  |  |  |
| 1 | Vvi-Vitvi05g01697\_t001 |  | Atr-ERN20164 |  |  |  |  |  |  |  |  |  |  |
| 1 | Vvi-Vitvi05g02279\_t001 |  | \| \| |  |  |  |  |  |  |  |  |  |  |
| 1 | Vvi-Vitvi05g04576\_t001 |  | Atr-ERN20163 |  |  |  |  |  |  |  |  |  |  |
| 1 | Vvi-Vitvi05g01700\_t001 |  | Atr-ERN20161 |  |  |  |  |  |  |  |  |  |  |
| 1 | Vvi-Vitvi05g01701\_t001 |  | Atr-ERN20158 |  |  |  |  |  |  |  |  |  |  |
| 1 | Vvi-Vitvi05g01702\_t001 |  | Atr-ERN20156 |  |  |  |  |  |  |  |  |  |  |
| 0 | Vvi-Vitvi05g01703\_t001 |  |  |  |  |  |  |  |  |  |  |  |  |
| 0 | Vvi-Vitvi05g01704\_t001 |  |  |  |  |  |  |  |  |  |  |  |  |
| 0 | Vvi-Vitvi05g04577\_t001 |  |  |  |  |  |  |  |  |  |  |  |  |
| 0 | Vvi-Vitvi05g04578\_t001 |  |  |  |  |  |  |  |  |  |  |  |  |
| 0 | Vvi-Vitvi05g04579\_t001 |  |  |  |  |  |  |  |  |  |  |  |  |
| 0 | Vvi-Vitvi05g01711\_t001 |  |  |  |  |  |  |  |  |  |  |  |  |
| 0 | Vvi-Vitvi05g01712\_t001 |  |  |  |  |  |  |  |  |  |  |  |  |
| 0 | Vvi-Vitvi05g01714\_t001 |  |  |  |  |  |  |  |  |  |  |  |  |
| 0 | Vvi-Vitvi05g04580\_t001 |  |  |  |  |  |  |  |  |  |  |  |  |
| 0 | Vvi-Vitvi05g04581\_t001 |  |  |  |  |  |  |  |  |  |  |  |  |
| 0 | Vvi-Vitvi05g01715\_t001 |  |  |  |  |  |  |  |  |  |  |  |  |
| 0 | Vvi-Vitvi05g04582\_t001 |  |  |  |  |  |  |  |  |  |  |  |  |
| 0 | Vvi-Vitvi05g01716\_t001 |  |  |  |  |  |  |  |  |  |  |  |  |
| 0 | Vvi-Vitvi05g02281\_t001 |  |  |  |  |  |  |  |  |  |  |  |  |
| 0 | Vvi-Vitvi05g04583\_t001 |  |  |  |  |  |  |  |  |  |  |  |  |
| 0 | Vvi-Vitvi05g04584\_t001 |  |  |  |  |  |  |  |  |  |  |  |  |
| 0 | Vvi-Vitvi05g04585\_t001 |  |  |  |  |  |  |  |  |  |  |  |  |
| 0 | Vvi-Vitvi05g04586\_t001 |  |  |  |  |  |  |  |  |  |  |  |  |
| 0 | Vvi-Vitvi05g04587\_t001 |  |  |  |  |  |  |  |  |  |  |  |  |
| 0 | Vvi-Vitvi05g04588\_t001 |  |  |  |  |  |  |  |  |  |  |  |  |
| 0 | Vvi-Vitvi05g04589\_t001 |  |  |  |  |  |  |  |  |  |  |  |  |
